# Bipolar complex q-rung orthopair fuzzy aggregation operators for enhanced decision-making in uncertain environments

**DOI:** 10.1038/s41598-025-32730-3

**Published:** 2025-12-31

**Authors:** Ibtesam Alshammari

**Affiliations:** Department of Mathematics, College of Sciences, University of Hafr Al Batin, Hafr Al Batin, Saudi Arabia

**Keywords:** Bipolar complex q-rung orthopair fuzzy sets, Aggregation methods, Uncertainty modeling, Decision support systems, Sustainable decision-making, Engineering, Mathematics and computing

## Abstract

Effective decision-making in uncertain and complex environments requires managing multidimensional, conflicting, and partially contradictory information. Existing fuzzy extensions—such as bipolar, q-rung orthopair, and complex fuzzy sets—address only parts of this uncertainty: bipolar sets handle positive and negative evaluations, q-rung orthopair sets allow flexible weighting of membership and nonmembership, and complex fuzzy sets capture phase-dependent or oscillatory information. However, none of these frameworks alone can simultaneously manage all these aspects. To overcome these challenges, this study introduces a bipolar complex q-rung orthopair fuzzy set (BCq-ROFS), which integrates bipolarity, complex membership structures, and q-rung orthopair fuzzy logic into a unified framework. Two aggregation mechanisms—BCq-ROF weighted averaging (BCq-ROFWA) and BCq-ROF weighted geometric (BCq-ROFWG) operators—are developed to effectively combine bipolar and complex fuzzy data across multiple attributes while maintaining a manageable computational cost. The framework applies to a multi-attribute decision-making problem in sustainable livestock farming, a domain characterized by conflicting objectives, resource limitations, and environmental–economic trade-offs. Results reveal that BCq-ROFS-based operators provide more stable, interpretable, and discriminative rankings than traditional fuzzy approaches. Comparative and sensitivity analyses confirm the robustness and scalability of the method, demonstrating improvements in decision accuracy and practical relevance.

## Introduction

The fuzzy set (FS) theory, introduced by Zadeh^1^, transformed classical set theory by incorporating the notion of partial membership, thereby extending beyond the binary logic of inclusion and exclusion. In this framework, each element possesses a membership degree in the range [0, 1], enabling more precise representation of uncertainty and vagueness in domains such as control systems, artificial intelligence, and decision-making. To overcome the inability of classical fuzzy sets to represent hesitation, Atanassov^2^ proposed intuitionistic fuzzy sets (IFSs), which introduce a non-membership function $\mathscr {N}$ alongside the membership function $\mathscr {M}$, constrained by $\mathscr {M} + \mathscr {N} \le 1$, with the hesitation degree expressed as $1 - (\mathscr {M} + \mathscr {N})$. Building on this framework, Yager^3^ extended IFSs to Pythagorean fuzzy sets (PFSs), defined by $\mathscr {M}^2 + \mathscr {N}^2 \le 1$, allowing a wider range of membership and non-membership values and thus capturing expert judgment more effectively. Recognizing the growing complexity of decision-making problems, Yager^4^ further generalized this concept through q-rung orthopair fuzzy sets (q-ROFSs), where $\mathscr {M}^q + \mathscr {N}^q \le 1$, introducing the parameter *q* to enhance flexibility and adaptability in modeling uncertainty. Subsequently, Senapati and Yager^5^ developed Fermatean fuzzy sets (FFSs), a specific form of q-ROFSs with $q=3$, which further broadens the expressive capacity for handling indeterminate information. Complex set theory extends the classical notion of sets by allowing elements to be represented in the complex number domain, thereby incorporating both magnitude and phase information. Utilizing the properties of complex numbers, these sets enable more realistic modeling of systems influenced by both amplitude and phase variations. Ramot et al.^6^ pioneered complex fuzzy sets (CFSs), where each element’s membership value is expressed as a complex function $\mathscr {M} e^{i\mathscr {Q}}$, with $\mathscr {M}$ denoting the magnitude and $\mathscr {Q}$ representing the phase term that captures periodic or time-dependent uncertainty. To enhance this framework, Alkouri and Salleh^7^ introduced complex intuitionistic fuzzy sets (CIFSs), incorporating complex-valued membership and non-membership functions bounded within the unit interval—an approach well-suited for applications such as medical diagnosis, pattern recognition, and artificial intelligence. Building upon these ideas, Ullah et al.^8^ proposed complex Pythagorean fuzzy sets (CPFSs), where the membership and non-membership functions satisfy the Pythagorean condition in the complex plane, providing greater flexibility for modeling uncertainty in machine learning and decision analysis. Further generalization was achieved by Liu et al.^9^ through complex q-rung orthopair fuzzy sets (Cq-ROFSs), defined by $\mathscr {M}^q + \mathscr {N}^q \le 1$, introducing the parameter *q* to regulate the degree of uncertainty and hesitation. More recently, Chinnadurai et al.^10^ developed complex Fermatean fuzzy sets (CFFSs), a special case of Cq-ROFSs with $q=3$, which extend the ability to represent imprecise and oscillatory information in engineering, quantum mechanics, and signal processing.

Bipolar fuzzy sets (BFSs) extend classical fuzzy theory by introducing positive and negative membership functions, enabling the simultaneous representation of degrees of acceptance and rejection and allowing the modeling of information with both favorable and unfavorable tendencies. Zhang^11^ initially proposed BFSs as a framework for cognitive modeling and multi-agent decision-making, later refining the concept into Yin–Yang bipolar fuzzy sets^12^ to capture dual aspects of uncertainty. Ezhilmaran and Sankar^13^ further developed bipolar intuitionistic fuzzy sets (BIFSs), integrating bipolar membership and non-membership functions for a more comprehensive treatment of uncertainty. Mohana and Jansi^14^ advanced this framework through bipolar Pythagorean fuzzy sets (BPFSs), where the membership and non-membership values satisfy a Pythagorean condition, providing greater flexibility in multi-criteria decision-making with opposing factors. Ibrahim^15^ generalized the approach to bipolar q-rung orthopair fuzzy sets (Bq-ROFSs), introducing the parameter *q* to control uncertainty and hesitation in complex group decision-making scenarios, while Fahmi et al.^16^ proposed bipolar Fermatean fuzzy sets (BFFSs), a special case of Bq-ROFSs with $q=3$, further enhancing the modeling of imprecise and oscillatory information. Extending these ideas into the complex domain, bipolar complex fuzzy sets (BCFSs) combine the dual membership nature of BFSs with complex-valued representations, where each membership function is expressed in terms of real and imaginary components, capturing both positive and negative memberships alongside additional dimensions of uncertainty. Mahmood and Ur Rehman^17^ applied BCFSs to generalized similarity measures, demonstrating their utility in pattern recognition, decision analysis, and system optimization. Building upon this foundation, bipolar complex intuitionistic fuzzy sets (BCIFSs) incorporate both membership and non-membership functions as complex numbers, providing a flexible structure for representing dual-aspect uncertainties. Mahmood et al.^18^ formally defined BCIFSs and analyzed their properties, and subsequent applications, as demonstrated in studies by Alkouri and Alshboul^19^ and by Ibrahim and Alqahtani^20^, have shown their practical utility in environmental impact assessment, sustainable waste-to-energy alternatives, and multi-attribute decision-making, offering a robust framework for modeling complex, multidimensional uncertainties in real-world decision-support problems.

Fuzzy set theory and its extensions have become essential tools for handling uncertainty, vagueness, and complexity in multi-criteria decision-making problems. The concept of complex membership grades provides a novel interpretation for representing uncertain information^21^, while multi-criteria approaches have been employed in assessing the sustainability of small-scale cooking and sanitation technologies^22^, societal development patterns^23^, and financial efficiency in Sub-Saharan Africa^24^. Research on green travel intentions^25^, sustainable urban planning using geospatial technologies^26^, and rainfall forecasting with enhanced Facebook Prophet models^27^ demonstrates the growing practical relevance of decision-making frameworks. Advanced aggregation and group decision-making techniques, such as interval-valued probabilistic linguistic T-spherical fuzzy information^28^, Dombi aggregation operators for p, q, r-spherical fuzzy sets^29^, and confidence-level-based p, q, r-spherical fuzzy aggregation operators^30^, have further strengthened decision-making accuracy. Hybrid CRITIC-EDAS models employing linguistic T-spherical fuzzy Hamacher aggregation^31^, bipolar complex fuzzy credibility operators^32^, confidence-level bipolar complex fuzzy operators^33^, fuzzy Ostrowski integral inequalities^34^, and fuzzy N-bipolar soft sets^35^ offer robust solutions for complex decision scenarios. Moreover, complex intuitionistic fuzzy classes^36^, and linguistic or 2-tuple linguistic complex intuitionistic fuzzy aggregation operators^37,38^ facilitate the modeling of uncertain, conflicting, and linguistic information in multi-criteria problems.

Recent studies highlight the practical applications of Pythagorean, Fermatean, and q-rung orthopair fuzzy aggregation operators across various domains. Methods like TODIM under t-arbicular fuzzy environments^39^, complex hesitant fuzzy partitioned Maclaurin symmetric mean operators with SWARA^40^, and N-cubic fuzzy aggregation operators^41^ have been applied in sustainable supply chains and environmental decisions^42^. Pythagorean cubic fuzzy Einstein aggregation operators support investment management^43^, while complex Pythagorean fuzzy sets assist in visualization technologies^44^. Applications extend to biomedical waste disposal^45,46^, hostel site selection^47^, low-carbon green supply chain management^48,49^, medical diagnostics^50^, construction material selection^51^, interactive aggregation of q-rung orthopair fuzzy soft sets^52,53^, precision agriculture^54^, transportation decisions in supply chains^55^, hybrid structures for waterborne disease diagnosis^56^, site selection for electric vehicle charging stations^57^, carbon capture technology selection^58^, optimal vehicle selection^59^, plastic waste management solution selection^60^, water purification strategies^61^, COVID-19 risk assessment^62^, and 5G network provider evaluation using TODIM-based cubic quasi-rung orthopair fuzzy MCGDM^63^. Moreover, recent studies have applied bipolar complex fuzzy aggregation operators, complex T-spherical fuzzy sets, and q-rung orthopair fuzzy rough aggregation for advanced decision-making in multiple domains^64–66^.

Despite advances in fuzzy set theory, no existing MADM frameworks simultaneously address bipolar evaluations, complex membership structures, and multidimensional uncertainty in practical decision contexts. Conventional methods, including AHP, TOPSIS, and VIKOR, often assume precise data and deterministic judgments, limiting their applicability in real-world systems such as sustainable agriculture and resource management, where uncertainty, heterogeneity, and conflicting objectives are inherent. This gap motivates the development of BCq-ROFS, a unified framework that integrates these aspects to enable robust and discriminative multi-attribute decision-making^67^. The proposed BCq-ROFS-based aggregation operators, BCq-ROFWA and BCq-ROFWG, combine bipolarity, complex membership structures, and the flexible q-parameter to support evaluation across multiple attributes. In this study, the framework is applied to sustainable livestock farming in Saudi Arabia, a sector facing challenges such as water scarcity, limited feed resources, disease management, and the need to enhance productivity sustainably. Alternative practices, including rotational grazing, integrated livestock–crop systems, hydroponic feed production, and smart farming technologies, are comparatively evaluated across multidimensional criteria, producing robust and discriminative rankings that support informed, sustainable decisions.

This study advances MADM under uncertainty through several key contributions:

1. A novel BCq-ROFS framework is proposed, integrating bipolarity, complex membership, and q-rung orthopair structures to capture both qualitative and quantitative uncertainties, with formal mathematical foundations and illustrative examples ensuring theoretical rigor and practical interpretability.
2. Two aggregation operators, BCq-ROFWA and BCq-ROFWG, are developed to systematically combine multi-attribute information while preserving the bipolar and complex characteristics of decision data, with thorough analysis of the parameter *q* to demonstrate stability, monotonic consistency, and robust ranking outcomes.
3. The framework is applied to optimize sustainable livestock farming practices, demonstrating enhanced decision-making reliability, flexibility, and practical utility under real-world uncertainty, supported by comparative analyses with existing fuzzy MADM methods showing superior accuracy and ability to model complex interdependencies.
4. Graphical visualizations and theoretical extensions illustrate the behavior of the aggregation operators and lay the foundation for future work, including applications to large-scale problems, dynamic decision contexts, and integration with intelligent decision-support systems.

The paper is organized as follows: Section 2 reviews related works on MADM, focusing on bipolar, complex, and q-ROFS fuzzy sets, and identifies the research gaps addressed in this study. Section 3 introduces the BCq-ROFS framework, detailing its definitions, properties, subset relations, complement operations, and algebraic characteristics with illustrative examples. Section 4 develops novel aggregation operators for BCq-ROFS, including BCq-ROFWA and BCq-ROFWG, along with their mathematical formulations and properties. Section 5 applies BCq-ROFS to a real-world MADM problem in sustainable livestock farming, demonstrating its ability to handle uncertain and bipolar information effectively. Section 7 presents a comparative evaluation of BCq-ROFS against other fuzzy models, using numerical results and graphical visualizations to illustrate performance, decision-making efficiency, and interpretability. Section 10 provides a sensitivity analysis of the BCq-ROFWA and BCq-ROFWG operators, assessing stability under varying *q* values and discussing practical limitations. Section 11 concludes the paper by summarizing key findings and contributions, and suggesting future research directions, including computational improvements, dynamic decision-making applications, and extensions to fields like finance and medical diagnostics.

## Preliminaries

This section lays the groundwork by presenting key foundational concepts.

### Definition 2.1

Consider a universal set $\mathscr {U}$. We define a set $\mathcal {H}$ as:

$$\mathcal {H}=\left\{ \left\langle \nu , \mathscr {M}(\nu ), \mathscr {N}(\nu )\right\rangle : \nu \in \mathscr {U}\right\}$$,

where $$\mathscr {M}: \mathscr {U} \rightarrow \mathscr{M}\mathscr{C}: \mathscr{M}\mathscr{C} \in \mathcal {H}, |\mathscr{M}\mathscr{C}| \le 1$$ and

$\mathscr {N}: \mathscr {U} \rightarrow \mathscr{N}\mathscr{C}: \mathscr{N}\mathscr{C} \in \mathcal {H}, |\mathscr{N}\mathscr{C}| \le 1$ satisfy the conditions:

$$\mathscr {M}(\nu ) = \mathscr{M}\mathscr{C} = r_1 + i m_1, \quad \mathscr {N}(\nu ) = \mathscr{N}\mathscr{C} = r_2 + i m_2,$$

with the constraint:

$$0 \le |\mathscr{M}\mathscr{C}|^q + |\mathscr{N}\mathscr{C}|^q \le 1.$$

Depending on the value of *q*, $\mathcal {H}$ corresponds to different fuzzy set models:

1. CIFS^7^ for $$q = 1$$.
2. CPFS^8^ for $$q = 2$$.
3. CFFS^10^ for $$q = 3$$.
4. Cq-ROFS^9^ for $q > 3$.

### Definition 2.2

Given a universal set $\mathscr {U}$, we define:

$$\mathcal {H} = \left\{ \left\langle \nu , \mathscr {M} _{\mathcal {H}}^{+}(\nu ), \mathscr {N}_{\mathcal {H}}^{+}(\nu ), \mathscr {M}_{\mathcal {H}}^{-}(\nu ), \mathscr {N}_{\mathcal {H}}^{-}(\nu )\right\rangle : \nu \in \mathscr {U} \right\}$$

where $\mathcal {H}$ takes different forms based on specific constraints:

1. BIFS^13^ if $0 \le \mathscr {M}_{\mathcal {H}}^{+} + \mathscr {N}_{\mathcal {H}}^{+} \le 1$, and $-1 \le \mathscr {M}_{\mathcal {H}}^{-} + \mathscr {N}_{\mathcal {H}}^{-} \le 0$.
2. BPFS^14,42^ if $0 \le (\mathscr {M}_{\mathcal {H}}^{+})^2 + (\mathscr {N}_{\mathcal {H}}^{+})^2 \le 1$, and $0 \le (\mathscr {M}_{\mathcal {H}}^{-})^2 + (\mathscr {N}_{\mathcal {H}}^{-})^2 \le 1$.
3. BFFS^16^ if $$0 \le (\mathscr {M}_{\mathcal {H}}^{+})^3 (\mathscr {N}_{\mathcal {H}}^{+})^3 + (\mathscr {M}_{\mathcal {H}}^{-})^3 (\mathscr {N}_{\mathcal {H}}^{-})^3 \le 1$$.
4. Bq-ROFS^15^ if $0 \le (\mathscr {M}_{\mathcal {H}}^{+})^{n} + (\mathscr {N}_{\mathcal {H}}^{+})^{m} \le 1$, and $0 \le |\mathscr {M}_{\mathcal {H}}^{-}|^{n} + |\mathscr {N}_{\mathcal {H}}^{-}|^{m} \le 1$, where $n = m = q$.

### Definition 2.3

^18^ Let $\mathscr {U}$ be a universal set. Then,

$$\mathcal {H}=\left\{ \left\langle \nu , \mathscr {M}_{\mathcal {H}}^{+}(\nu ) +i \mathscr {A}_{\mathcal {H}}^{+}(\nu ),\mathscr {N}_{\mathcal {H}}^{+}(\nu ) +i \mathscr {B}_{\mathcal {H}}^{+}(\nu ), \right. \right.$$

$$\left. \left. \mathscr {M}_{\mathcal {H}}^{-}(\nu ) +i \mathscr {A}_{\mathcal {H}}^{-}(\nu ), \mathscr {N}_{\mathcal {H}}^{-}(\nu ) +i \mathscr {B}_{\mathcal {H}}^{-}(\nu )\right\rangle : \nu \in \mathscr {U} \right\}$$

is called a BCIFS if

$$0 \le \mathscr {M}_{\mathcal {H}}^{+}(\nu ) + \mathscr {N}_{\mathcal {H}}^{+}(\nu ) \le 1,\quad 0 \le \mathscr {A}_{\mathcal {H}}^{+}(\nu ) + \mathscr {B}_{\mathcal {H}}^{+}(\nu ) \le 1;$$

$$0 \le |\mathscr {M}_{\mathcal {H}}^{-}(\nu )|+ |\mathscr {N}_{\mathcal {H}}^{-}(\nu )|\le 1,\quad 0 \le |\mathscr {A}_{\mathcal {H}}^{-}(\nu )|+ |\mathscr {B}_{\mathcal {H}}^{-}(\nu )|\le 1,$$

where

$$\begin{aligned} \mathscr {M}_{\mathcal {H}}^{+}(\nu ),\, \mathscr {N}_{\mathcal {H}}^{+}(\nu ), \mathscr {A}_{\mathcal {H}}^{+}(\nu ),\, \mathscr {B}_{\mathcal {H}}^{+}(\nu )&\in [0,1], \end{aligned}$$

$$\begin{aligned} \mathscr {M}_{\mathcal {H}}^{-}(\nu ),\, \mathscr {N}_{\mathcal {H}}^{-}(\nu ), \mathscr {A}_{\mathcal {H}}^{-}(\nu ),\, \mathscr {B}_{\mathcal {H}}^{-}(\nu )&\in [-1,0]. \end{aligned}$$

## Bipolar complex q-rung orthopair fuzzy sets

Bipolar fuzzy sets extend classical fuzzy sets by assigning each element both a positive membership degree, representing acceptance or satisfaction, and a negative membership degree, representing rejection or opposition. This dual evaluation allows for a realistic representation of situations where favorable and unfavorable aspects coexist, reflecting the nuances of human judgment and complex decision-making. When combined with complex numbers and the q-rung orthopair structure, bipolar fuzzy sets can model both real and imaginary components, as well as multidimensional uncertainty and conflicting preferences.

This section then presents a detailed description of the fundamental concepts and essential operations related to bipolar complex q-ROFS.

### Definition 3.1

In the universe of discourse $\mathscr {U}$, a bipolar complex q-rung orthopair fuzzy set (BCq-ROFS) $\mathcal {H}$ is characterized as follows:

$$\mathcal {H}=\left\{ \left\langle \nu , \mathscr {M}_{\mathcal {H}}^{+}(\nu ) +i \mathscr {A}_{\mathcal {H}}^{+}(\nu ),\mathscr {N}_{\mathcal {H}}^{+}(\nu ) +i \mathscr {B}_{\mathcal {H}}^{+}(\nu ), \right. \right.$$

$$\left. \left. \mathscr {M}_{\mathcal {H}}^{-}(\nu ) +i \mathscr {A}_{\mathcal {H}}^{-}(\nu ), \mathscr {N}_{\mathcal {H}}^{-}(\nu ) +i \mathscr {B}_{\mathcal {H}}^{-}(\nu )\right\rangle : \nu \in \mathscr {U} \right\}$$

where

$$\begin{aligned} \mathscr {M}_{\mathcal {H}}^{+}(\nu ),\, \mathscr {N}_{\mathcal {H}}^{+}(\nu )&\in [0,1] \quad \text {(real positive components)},\\ \mathscr {A}_{\mathcal {H}}^{+}(\nu ),\, \mathscr {B}_{\mathcal {H}}^{+}(\nu )&\in [0,1] \quad \text {(imaginary positive components)}, \end{aligned}$$

with

$$0 \le (\mathscr {M}_{\mathcal {H}}^{+}(\nu ))^{q} + (\mathscr {N}_{\mathcal {H}}^{+}(\nu ))^{q} \le 1,\quad 0 \le (\mathscr {A}_{\mathcal {H}}^{+}(\nu ))^{q} + (\mathscr {B}_{\mathcal {H}}^{+}(\nu ))^{q} \le 1;$$

and

$$\begin{aligned} \mathscr {M}_{\mathcal {H}}^{-}(\nu ),\, \mathscr {N}_{\mathcal {H}}^{-}(\nu )&\in [-1,0] \quad \text {(real negative components)},\\ \mathscr {A}_{\mathcal {H}}^{-}(\nu ),\, \mathscr {B}_{\mathcal {H}}^{-}(\nu )&\in [-1,0] \quad \text {(imaginary negative components)}, \end{aligned}$$

with

$$0 \le |\mathscr {M}_{\mathcal {H}}^{-}(\nu )|^{q} + |\mathscr {N}_{\mathcal {H}}^{-}(\nu )|^{q} \le 1,\quad 0 \le |\mathscr {A}_{\mathcal {H}}^{-}(\nu )|^{q} + |\mathscr {B}_{\mathcal {H}}^{-}(\nu )|^{q} \le 1.$$

The value of $\nu$ is computed as

$$\mathcal {H} = \langle \mathscr {M}_{\mathcal {H}}^{+} + i \mathscr {A}_{\mathcal {H}}^{+}, \mathscr {N}_{\mathcal {H}}^{+} + i \mathscr {B}_{\mathcal {H}}^{+}, \mathscr {M}_{\mathcal {H}}^{-} + i \mathscr {A}_{\mathcal {H}}^{-}, \mathscr {N}_{\mathcal {H}}^{-} + i \mathscr {B}_{\mathcal {H}}^{-} \rangle$$

which represents the BCq-ROF number (BCq-ROFN).

**Fig. 1:**
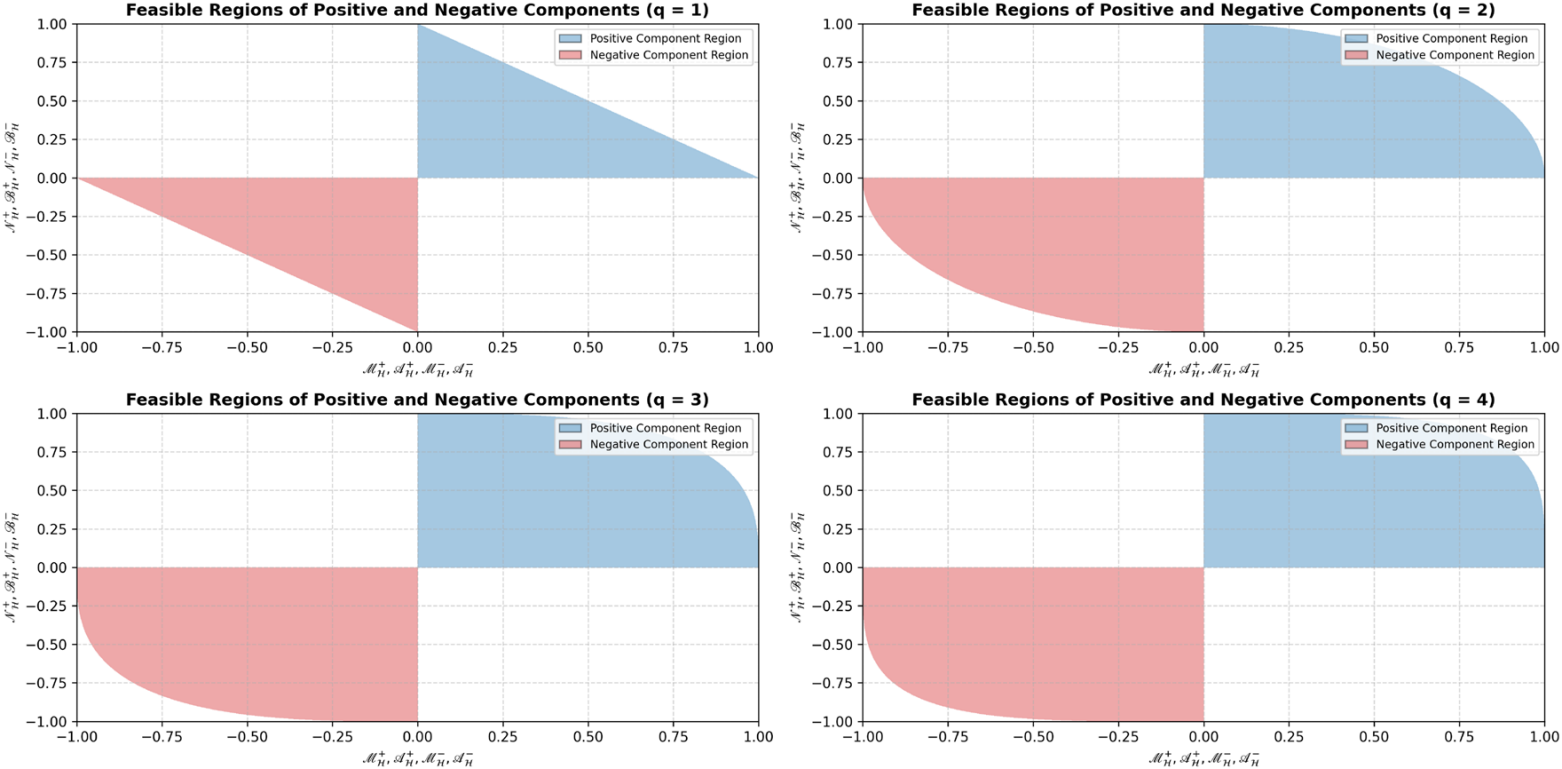
Grades space of BCq-ROF values for $q=1,2,3,4$.

Figure 1 illustrates the feasible space of bipolar complex q-rung orthopair fuzzy values, showing the interactions between the real and imaginary components for both positive and negative memberships. The figure highlights the admissible regions for different *q* values, with blue representing positive components and red representing negative components. This visualization clarifies the distinct behaviors and constraints of the bipolar complex components, providing a clear understanding of their relationships and how the q-rung structure shapes the feasible space.

### Example 3.2

Consider a decision-making scenario in which a company evaluates two renewable energy projects, $\mathscr {P}_1$ and $\mathscr {P}_2$, based on two attributes: economic viability ($\mathscr{A}\mathscr{T}_1$) and environmental impact ($\mathscr{A}\mathscr{T}_2$). The evaluations are expressed using BC2-ROFSs as follows:

$$\begin{aligned} {\mathcal{H}}_{{11}} = & \langle 0.8 + 0.6i,0.2 + 0.1i, - 0.3 - 0.2i, - 0.1 - 0.05i\rangle , \\ {\mathcal{H}}_{{12}} = & \langle 0.7 + 0.5i,0.3 + 0.1i, - 0.2 - 0.1i, - 0.05 - 0.02i\rangle , \\ {\mathcal{H}}_{{21}} = & \langle 0.6 + 0.4i,0.3 + 0.2i, - 0.1 - 0.05i, - 0.2 - 0.1i\rangle , \\ {\mathcal{H}}_{{22}} = & \langle 0.9 + 0.7i,0.1 + 0.05i, - 0.4 - 0.3i, - 0.1 - 0.02i\rangle . \\ \end{aligned}$$

Here:

- $\mathscr {M}^+ + i \mathscr {A}^+$: positive membership (supporting evidence),
- $\mathscr {N}^+ + i \mathscr {B}^+$: positive non-membership (degree of not supporting),
- $\mathscr {M}^- + i \mathscr {A}^-$: negative membership (opposing evidence),
- $\mathscr {N}^- + i \mathscr {B}^-$: negative non-membership (degree of not opposing).

### Definition 3.3

For any two BCq-ROFNs, let the following be defined:

$$\mathcal {H}_1 = \left\langle \mathscr {M}_{\mathcal {H}_1}^{+} + i \mathscr {A}_{\mathcal {H}_1}^{+}, \mathscr {N}_{\mathcal {H}_1}^{+} + i \mathscr {B}_{\mathcal {H}_1}^{+}, \mathscr {M}_{\mathcal {H}_1}^{-} + i \mathscr {A}_{\mathcal {H}_1}^{-}, \mathscr {N}_{\mathcal {H}_1}^{-} + i \mathscr {B}_{\mathcal {H}_1}^{-} \right\rangle$$

and

$$\mathcal {H}_2 = \left\langle \mathscr {M}_{\mathcal {H}_2}^{+} + i \mathscr {A}_{\mathcal {H}_2}^{+}, \mathscr {N}_{\mathcal {H}_2}^{+} + i \mathscr {B}_{\mathcal {H}_2}^{+}, \mathscr {M}_{\mathcal {H}_2}^{-} + i \mathscr {A}_{\mathcal {H}_2}^{-}, \mathscr {N}_{\mathcal {H}_2}^{-} + i \mathscr {B}_{\mathcal {H}_2}^{-} \right\rangle .$$

We have: 

1. $\mathcal {H}_1 \subseteq \mathcal {H}_2$ if and only if

$$\mathscr {M}_{\mathcal {H}_1}^{+} \le \mathscr {M}_{\mathcal {H}_2}^{+}, \quad \mathscr {M}_{\mathcal {H}_1}^{-} \ge \mathscr {M}_{\mathcal {H}_2}^{-}, \quad \mathscr {N}_{\mathcal {H}_1}^{+} \ge \mathscr {N}_{\mathcal {H}_2}^{+}, \quad \mathscr {N}_{\mathcal {H}_1}^{-} \le \mathscr {N}_{\mathcal {H}_2}^{-}$$

for real terms and

$$\mathscr {A}_{\mathcal {H}_1}^{+} \le \mathscr {A}_{\mathcal {H}_2}^{+}, \quad \mathscr {A}_{\mathcal {H}_1}^{-} \ge \mathscr {A}_{\mathcal {H}_2}^{-}, \quad \mathscr {B}_{\mathcal {H}_1}^{+} \ge \mathscr {B}_{\mathcal {H}_2}^{+}, \quad \mathscr {B}_{\mathcal {H}_1}^{-} \le \mathscr {B}_{\mathcal {H}_2}^{-}$$

for imaginary terms.

2. $\mathcal {H}_1 = \mathcal {H}_2$ if and only if

$$\mathscr {M}_{\mathcal {H}_1}^{+} = \mathscr {M}_{\mathcal {H}_2}^{+}, \quad \mathscr {M}_{\mathcal {H}_1}^{-} = \mathscr {M}_{\mathcal {H}_2}^{-}, \quad \mathscr {N}_{\mathcal {H}_1}^{+} = \mathscr {N}_{\mathcal {H}_2}^{+}, \quad \mathscr {N}_{\mathcal {H}_1}^{-} = \mathscr {N}_{\mathcal {H}_2}^{-},$$

$$\mathscr {A}_{\mathcal {H}_1}^{+} = \mathscr {A}_{\mathcal {H}_2}^{+}, \quad \mathscr {A}_{\mathcal {H}_1}^{-} = \mathscr {A}_{\mathcal {H}_2}^{-}, \quad \mathscr {B}_{\mathcal {H}_1}^{+} = \mathscr {B}_{\mathcal {H}_2}^{+}, \quad \mathscr {B}_{\mathcal {H}_1}^{-} = \mathscr {B}_{\mathcal {H}_2}^{-}.$$

3.

1 $$\begin{aligned} {\mathcal {H}_1}^{c}=\begin{array}{c}\langle \mathscr {N}_{\mathcal {H}_1}^{+} +i \mathscr {B}_{\mathcal {H}_1}^{+},\mathscr {M}_{\mathcal {H}_1}^{+} +i \mathscr {A}_{\mathcal {H}_1}^{+}, -|\mathscr {N}_{\mathcal {H}_1}^{-}| +i (-|\mathscr {B}_{\mathcal {H}_1}^{-}|) , -|\mathscr {M}_{\mathcal {H}_1}^{-}| +i (-|\mathscr {A}_{\mathcal {H}_1}^{-}|)\rangle . \end{array} \end{aligned}$$

4.

2 $$\begin{aligned} \mathcal {H}_1 \cap \mathcal {H}_2= \begin{array}{c} \langle \min \{\mathscr {M}_{\mathcal {H}_1}^{+}, \mathscr {M}_{\mathcal {H}_2}^{+}\} +i \min \{\mathscr {A}_{\mathcal {H}_1}^{+}, \mathscr {A}_{\mathcal {H}_2}^{+}\}, \\ \max \{\mathscr {N}_{\mathcal {H}_1}^{+}, \mathscr {N}_{\mathcal {H}_2}^{+}\} +i \max \{\mathscr {B}_{\mathcal {H}_1}^{+}, \mathscr {B}_{\mathcal {H}_2}^{+}\},\\ \max \{\mathscr {M}_{\mathcal {H}_1}^{-}, \mathscr {M}_{\mathcal {H}_2}^{-}\} +i \max \{\mathscr {A}_{\mathcal {H}_1}^{-}, \mathscr {A}_{\mathcal {H}_2}^{-}\}, \\ \min \{\mathscr {N}_{\mathcal {H}_1}^{-}, \mathscr {N}_{\mathcal {H}_2}^{-}\} +i \min \{\mathscr {B}_ {\mathcal {H}_1}^{-}, \mathscr {B}_ {\mathcal {H}_2}^{-}\} \rangle . \end{array} \end{aligned}$$

5.

3 $$\begin{aligned} \mathcal {H}_1 \cup \mathcal {H}_2=\begin{array}{c}\langle \max \{\mathscr {M}_{\mathcal {H}_1}^{+}, \mathscr {M}_{\mathcal {H}_2}^{+}\} +i \max \{\mathscr {A}_{\mathcal {H}_1}^{+}, \mathscr {A} _{\mathcal {H}_2}^{+}\}, \\ \min \{\mathscr {N}_{\mathcal {H}_1}^{+}, \mathscr {N}_{\mathcal {H}_2}^{+}\} +i \min \{\mathscr {B}_{\mathcal {H}_1}^{+}, \mathscr {B}_ {\mathcal {H}_2}^{+}\}, \\ \min \{\mathscr {M}_{\mathcal {H}_1}^{-}, \mathscr {M}_{\mathcal {H}_2}^{-}\} +i \min \{\mathscr {A}_{\mathcal {H}_1}^{-}, \mathscr {A}_{\mathcal {H}_2}^{-}\}, \\ \max \{\mathscr {N}_{\mathcal {H}_1}^{-}, \mathscr {N}_{\mathcal {H}_2}^{-}\} +i \max \{\mathscr {B}_ {\mathcal {H}_1}^{-}, \mathscr {B}_ {\mathcal {H}_2}^{-}\} \rangle . \end{array} \end{aligned}$$

### Example 3.4

Define the set $\mathscr {U}=\left\{ \nu _{1},\nu _{2}, \nu _{3}\right\}$. To enhance conciseness and maintain consistency in numerical expressions, all decimal values within the open interval $(-1,1)$ are represented without a leading zero. Then, the set $\mathcal {H}_1$ is expressed as:

$$\mathcal {H}_1=\left\{ \begin{array}{c}\left\langle \nu _1, .8+(.2)i,.7+(.1)i,-.2+(-.2)i,-.7+(-.1)i\right\rangle , \\ \left\langle \nu _2, .4+(.1)i,.6+(.2)i,-.1+(-.1)i,-.2+(-.3)i\right\rangle , \\ \left\langle \nu _3, .3+(.3)i,.8+(.2)i,-.6+(-.1)i,-.5+(-.1)i\right\rangle \end{array}\right\}$$

Similarly, the set $\mathcal {H}_2$ is given by:

$$\mathcal {H}_2=$$ $$\left\{ \begin{array}{c}\left\langle \nu _1, .7+(.1)i,.8+(.2)i,-.7+(-.3)i,-.2+(-.5)i\right\rangle , \\ \left\langle \nu _2, .6+(.2)i,.4+(.1)i,-.2+(-.5)i,-.1+(-.3)i\right\rangle , \\ \left\langle \nu _3, .6+(.1)i,.5+(.2)i,-.8+(-.3)i,-.3+(-.5)i\right\rangle \end{array}\right\}$$.

Both $\mathcal {H}_1$ and $\mathcal {H}_2$ represent BC3-ROFNs. Consequently,

1. $$\mathcal {H}_{1}^{c}=\left\{ \begin{array}{c}\left\langle \nu _1, .7+(.1)i,.8+(.2)i,-.7+(-.1)i,-.2+(-.2)i\right\rangle , \\ \left\langle \nu _2, .6+(.2)i,.4+(.1)i,-.2+(-.3)i,-.1+(-.1)i\right\rangle , \\ \left\langle \nu _3, .8+(.2)i,.3+(.3)i,-.5+(-.1)i,-.6+(-.1)i\right\rangle \end{array}\right\}$$.
2. $$\mathcal {H}_1 \cap \mathcal {H}_2= \left\{ \begin{array}{c}\left\langle \nu _1, .7+(.1)i,.8+(.2)i,-.2+(-.2)i,-.7+(-.5)i\right\rangle \\ \left\langle \nu _2, .4+(.1)i,.6+(.2)i,-.1+(-.1)i,-.2+(-.3)i\right\rangle \\ \left\langle \nu _3, .3+(.1)i,.8+(.2)i,-.6+(-.1)i,-.5+(-.5)i\right\rangle \end{array}\right\}$$.
3. $$\mathcal {H}_1 \cup \mathcal {H}_2=\left\{ \begin{array}{c}\left\langle \nu _1, .8+(.2)i,.7+(.1)i,-.7+(-.3)i,-.2+(-.1)i\right\rangle \\ \left\langle \nu _2, .6+(.2)i,.4+(.1)i,-.2+(-.5)i,-.1+(-.3)i\right\rangle \\ \left\langle \nu _3, .6+(.3)i,.5+(.2)i,-.8+(-.3)i,-.3+(-.1)i \right\rangle \end{array}\right\}$$.

### Theorem 3.5

Let

$$\mathcal {H}=\left\langle \mathscr {M}_{\mathcal {H}}^{+}+i \mathscr {A}_{\mathcal {H}}^{+}, \mathscr {N}_{\mathcal {H}}^{+} +i \mathscr {B}_{\mathcal {H}}^{+}, \mathscr {M}_{\mathcal {H}}^{-} +i \mathscr {A}_{\mathcal {H}}^{-}, \mathscr {N}_{\mathcal {H}}^{-} +i \mathscr {B}_{\mathcal {H}}^{-}\right\rangle$$

be a BCq-ROFN. Similarly, define

$$\mathcal {H}_1 = \left\langle \mathscr {M}_{\mathcal {H}_{1}}^{+}+i \mathscr {A}_{\mathcal {H}_{1}}^{+}, \mathscr {N}_{\mathcal {H}_{1}}^{+} +i \mathscr {B}_{\mathcal {H}_{1}}^{+}, \mathscr {M}_{\mathcal {H}_{1}}^{-} +i \mathscr {A}_{\mathcal {H}_{1}}^{-}, \mathscr {N}_{\mathcal {H}_{1}}^{-} +i \mathscr {B}_{\mathcal {H}_{1}}^{-}\right\rangle$$

and

$$\mathcal {H}_2 = \left\langle \mathscr {M}_{\mathcal {H}_{2}}^{+}+i \mathscr {A}_{\mathcal {H}_{2}}^{+}, \mathscr {N}_{\mathcal {H}_{2}}^{+} +i \mathscr {B}_{\mathcal {H}_{2}}^{+}, \mathscr {M}_{\mathcal {H}_{2}}^{-} +i \mathscr {A}_{\mathcal {H}_{2}}^{-}, \mathscr {N}_{\mathcal {H}_{2}}^{-} +i \mathscr {B}_{\mathcal {H}_{2}}^{-}\right\rangle .$$

These represent BCq-ROFNs, and therefore, 

1. ${\mathcal {H}}^{c}$ is a BCq-ROFN, and applying the complement twice yields $({\mathcal {H}}^{c})^{c}=\mathcal {H}$.

2. 

$\mathcal {H}_{1}\cup \mathcal {H}_{2}$ and $\mathcal {H}_{1}\cap \mathcal {H}_{2}$ are also BCq-ROFN.

### Proof

1. Since

$$\begin{gathered} 0 \le ({\mathscr{M}}_{{\mathcal{H}}}^{ + } )^{q} + ({\mathscr{N}}_{{\mathcal{H}}}^{ + } )^{q} \le 1, \hfill \\ 0 \le ({\mathscr{A}}_{{\mathcal{H}}}^{ + } )^{q} + ({\mathscr{B}}_{{\mathcal{H}}}^{ + } )^{q} \le 1, \hfill \\ 0 \le \left| {{\mathscr{M}}_{{\mathcal{H}}}^{ - } } \right|^{q} + \left| {{\mathscr{N}}_{{\mathcal{H}}}^{ - } } \right|^{q} \le 1{\mkern 1mu} {\mkern 1mu} {\mkern 1mu} {\mkern 1mu} {\mkern 1mu} {\mkern 1mu} {\mkern 1mu} \hfill \\ \,\,\,\,\,\,\,\,\,\,\,\,\,\,\,and \hfill \\ 0 \le |{\mathscr{A}}_{{\mathcal{H}}}^{ - } |^{q} + |{\mathscr{B}}_{{\mathcal{H}}}^{ - } |^{q} \le 1 \hfill \\ \end{gathered}$$

then

$$\begin{gathered} 0 \le ({\mathscr{N}}_{{\mathcal{H}}}^{ + } )^{q} + ({\mathscr{M}}_{{\mathcal{H}}}^{ + } )^{q} = ({\mathscr{M}}_{{\mathcal{H}}}^{ + } )^{q} + ({\mathscr{N}}_{{\mathcal{H}}}^{ + } )^{q} \le 1, \hfill \\ 0 \le ({\mathscr{B}}_{{\mathcal{H}}}^{ + } )^{q} + ({\mathscr{A}}_{{\mathcal{H}}}^{ + } )^{q} = ({\mathscr{A}}_{{\mathcal{H}}}^{ + } )^{q} + ({\mathscr{B}}_{{\mathcal{H}}}^{ + } )^{q} \le 1, \hfill \\ 0 \le \left| { - \left| {{\mathscr{N}}_{{\mathcal{H}}}^{ - } } \right|} \right|^{q} + \left| { - \left| {{\mathscr{M}}_{{\mathcal{H}}}^{ - } } \right|} \right|^{q} = \left| {{\mathscr{M}}_{{\mathcal{H}}}^{ - } } \right|^{q} + \left| {{\mathscr{N}}_{{\mathcal{H}}}^{ - } } \right|^{q} \le 1 \hfill \\ \,\,\,\,\,\,\,\,\,\,\,\,\,\,\,\,\,\,\,\,\,\,\,\,\,\,\,\,\,\,\,{\mathrm{and}} \hfill \\ 0 \le \left| { - \left| {{\mathscr{B}}_{{\mathcal{H}}}^{ - } } \right|} \right|^{q} + \left| { - \left| {{\mathscr{A}}_{{\mathcal{H}}}^{ - } } \right|} \right|^{q} = |{\mathscr{A}}_{{\mathcal{H}}}^{ - } |^{q} + |{\mathscr{B}}_{{\mathcal{H}}}^{ - } |^{q} \le 1. \hfill \\ \end{gathered}$$

Hence, $\mathcal {H}^{c}$ is a BCq-ROFN, and it is evident that

$$\begin{gathered} ({\mathcal {H}}^{c})^{c}=\langle \mathscr {N}_\mathcal {H}^{+} +i \mathscr {B}_\mathcal {H}^{+},\mathscr {M}_\mathcal {H}^{+} +i \mathscr {A}_\mathcal {H}^{+}, -|\mathscr {N}_\mathcal {H}^{-}|+i (-|\mathscr {B}_\mathcal {H}^{-}|), -|\mathscr {M}_\mathcal {H}^{-}| +i (-|\mathscr {A}_\mathcal {H}^{-}|)\rangle ^{c}\\ = \langle \mathscr {M}_\mathcal {H}^{+} +i \mathscr {A}_\mathcal {H}^{+}, \mathscr {N}_\mathcal {H}^{+} +i \mathscr {B}_\mathcal {H}^{+}, -| -|\mathscr {M}_\mathcal {H}^{-}|| +i (-|-|\mathscr {A}_\mathcal {H}^{-}||), -|-|\mathscr {N}_\mathcal {H}^{-}|| +i (-|-|\mathscr {B}_\mathcal {H}^{-}||)\rangle \\ =\langle \mathscr {M}_{\mathcal {H}}^{+} +i \mathscr {A}_{\mathcal {H}}^{+},\mathscr {N}_{\mathcal {H}}^{+} +i \mathscr {B}_{\mathcal {H}}^{+}, \mathscr {M}_{\mathcal {H}}^{-} +i \mathscr {A}_{\mathcal {H}}^{-}, \mathscr {N}_{\mathcal {H}}^{-} +i \mathscr {B}_{\mathcal {H}}^{-}\rangle, \end{gathered}$$

where $$\mathscr {M}_{\mathcal {H}}^{-}= -\left| \mathscr {M}_{\mathcal {H}}^{-}\right| ,\quad \mathscr {N}_{\mathcal {H}}^{-}=-\left| \mathscr {N}_{\mathcal {H}}^{-}\right|$$, $$\mathscr {A}_{\mathcal {H}}^{-}= -\left| \mathscr {A}_{\mathcal {H}}^{-}\right|$$, and $$\quad \mathscr {B}_{\mathcal {H}}^{-}=-\left| \mathscr {B}_{\mathcal {H}}^{-}\right|$$.

2. 

Since the following inequalities hold:

$$0 \le \mathscr {M}_{\mathcal {H}_1}^{+}, \mathscr {A}_{\mathcal {H}_1}^{+}, \mathscr {M}_{\mathcal {H}_2}^{+}, \mathscr {A}_{\mathcal {H}_2}^{+}, \mathscr {N}_{\mathcal {H}_1}^{+},\mathscr {B}_{\mathcal {H}_1}^{+}, \mathscr {N}_{\mathcal {H}_2}^{+}, \mathscr {B}_{\mathcal {H}_2}^{+} \le 1,$$

and

$$0 \le |\mathscr {M}_{\mathcal {H}_1}^{-}|, |\mathscr {A}_{\mathcal {H}_1}^{-}|, |\mathscr {M}_{\mathcal {H}_2}^{-}|, |\mathscr {A}_{\mathcal {H}_2}^{-}|, |\mathscr {N}_{\mathcal {H}_1}^{-}|,|\mathscr {B}_{\mathcal {H}_1}^{-}|, |\mathscr {N}_{\mathcal {H}_2}^{-}|,|\mathscr {B}_{\mathcal {H}_2}^{-}| \le 1,$$

it is evident that the following conditions hold:

$$0 \le \left( \max \left\{ \mathscr {M}_{\mathcal {H}_1}^{+}, \mathscr {M}_{\mathcal {H}_2}^{+} \right\} \right) ^q + \left( \min \left\{ \mathscr {N}_{\mathcal {H}_1}^{+}, \mathscr {N}_{\mathcal {H}_2}^{+} \right\} \right) ^q \le 1,$$

$$0 \le \left( \max \left\{ \mathscr {A}_{\mathcal {H}_1}^{+}, \mathscr {A}_{\mathcal {H}_2}^{+} \right\} \right) ^q + \left( \min \left\{ \mathscr {B}_{\mathcal {H}_1}^{+}, \mathscr {B}_{\mathcal {H}_2}^{+} \right\} \right) ^q \le 1,$$

$$0 \le \left( \min \left\{ |\mathscr {M}_{\mathcal {H}_1}^{-}|, |\mathscr {M}_{\mathcal {H}_2}^{-}| \right\} \right) ^q + \left( \max \left\{ |\mathscr {N}_{\mathcal {H}_1}^{-}|, |\mathscr {N}_{\mathcal {H}_2}^{-}| \right\} \right) ^q \le 1,$$

and

$$0 \le \left( \max \left\{ |\mathscr {B}_{\mathcal {H}_1}^{-}|, |\mathscr {B}_{\mathcal {H}_2}^{-}| \right\} \right) ^q + \left( \min \left\{ |\mathscr {A}_{\mathcal {H}_1}^{-}|, |\mathscr {A}_{\mathcal {H}_2}^{-}| \right\} \right) ^q \le 1.$$

Therefore, $\mathcal {H}_1 \cup \mathcal {H}_2$ is a BCq-ROFN. A similar proof can be applied to show that $\mathcal {H}_1 \cap \mathcal {H}_2$ is also a BCq-ROFN.

$$\square$$

### Theorem 3.6

Let $${\mathcal{H}}$$$$=$$$$\langle {\mathscr{M}}_{{\mathcal{H}}}^{ + }$$$$+ i{\mathscr{A}}_{{\mathcal{H}}}^{ + }$$$$,{\mathscr{N}}_{{\mathcal{H}}}^{ + }$$$$+ i{\mathscr{B}}_{{\mathcal{H}}}^{ + }$$$$,{\mathscr{M}}_{{\mathcal{H}}}^{ - }$$$$+ i{\mathscr{A}}_{{\mathcal{H}}}^{ - } ,$$$${\mathscr{N}}_{{\mathcal{H}}}^{ - }$$$$+ i{\mathscr{B}}_{{\mathcal{H}}}^{ - } \rangle$$,    $${\mathcal{H}}_{1}$$$$=$$$$\langle {\mathscr{M}}_{{{\mathcal{H}}_{1} }}^{ + }$$$$+ i{\mathscr{A}}_{{{\mathcal{H}}_{1} }}^{ + }$$$$,{\mathscr{N}}_{{{\mathcal{H}}_{1} }}^{ + }$$$$+ i{\mathscr{B}}_{{{\mathcal{H}}_{1} }}^{ + }$$$$,{\mathscr{M}}_{{{\mathcal{H}}_{1} }}^{ - }$$$$+ i{\mathscr{A}}_{{{\mathcal{H}}_{1} }}^{ - } ,$$$${\mathscr{N}}_{{{\mathcal{H}}_{1} }}^{ - }$$$$+ i{\mathscr{B}}_{{{\mathcal{H}}_{1} }}^{ - } \rangle$$, and $$\mathcal {H}_2 = \langle \mathscr {M}_{\mathcal {H}_2}^{+} + i \mathscr {A}_{\mathcal {H}_2}^{+}, \mathscr {N}_{\mathcal {H}_2}^{+} + i \mathscr {B}_{\mathcal {H}_2}^{+}, \mathscr {M}_{\mathcal {H}_2}^{-} + i \mathscr {A}_{\mathcal {H}_2}^{-}, \mathscr {N}_{\mathcal {H}_2}^{-} + i \mathscr {B}_{\mathcal {H}_2}^{-} \rangle$$ be BCq-ROFNs. Then the following properties hold:

1. $$\mathcal {H}_2 \cap \mathcal {H}_1=\mathcal {H}_1 \cap \mathcal {H}_2$$.
2. $$\mathcal {H}_2 \cup \mathcal {H}_1=\mathcal {H}_1 \cup \mathcal {H}_2$$.
3. $$\mathcal {H}_2=(\mathcal {H}_1 \cup \mathcal {H}_2) \cap \mathcal {H}_2$$.
4. $$\mathcal {H}_2=(\mathcal {H}_1 \cap \mathcal {H}_2) \cup \mathcal {H}_2$$.
5. $$(\mathcal {H} \cap \mathcal {H}_1) \cap \mathcal {H}_2=\mathcal {H} \cap (\mathcal {H}_1 \cap \mathcal {H}_2)$$.
6. $$(\mathcal {H} \cup \mathcal {H}_1) \cup \mathcal {H}_2=\mathcal {H} \cup (\mathcal {H}_1 \cup \mathcal {H}_2)$$.
7. $$(\mathcal {H} \cup \mathcal {H}_2) \cap (\mathcal {H}_1 \cup \mathcal {H}_2)=(\mathcal {H} \cap \mathcal {H}_1) \cup \mathcal {H}_2$$.
8. $$(\mathcal {H} \cap \mathcal {H}_2) \cup (\mathcal {H}_1 \cap \mathcal {H}_2)=(\mathcal {H} \cup \mathcal {H}_1) \cap \mathcal {H}_2$$.

### Proof

The statements are straightforward. Therefore, the results are obvious. $\square$

### Theorem 3.7

Let $$\mathcal {H}_1 = \langle \mathscr {M}_{\mathcal {H}_1}^{+} + i \mathscr {A}_{\mathcal {H}_1}^{+}, \mathscr {N}_{\mathcal {H}_1}^{+} + i \mathscr {B}_{\mathcal {H}_1}^{+}, \mathscr {M}_{\mathcal {H}_1}^{-} + i \mathscr {A}_{\mathcal {H}_1}^{-}, \mathscr {N}_{\mathcal {H}_1}^{-} + i \mathscr {B}_{\mathcal {H}_1}^{-} \rangle$$ and $$\mathcal {H}_2$$$$=$$$$\langle \mathscr {M}_{\mathcal {H}_2}^{+} + i \mathscr {A}_{\mathcal {H}_2}^{+}, \mathscr {N}_{\mathcal {H}_2}^{+} + i \mathscr {B}_{\mathcal {H}_2}^{+}, \mathscr {M}_{\mathcal {H}_2}^{-} + i \mathscr {A}_{\mathcal {H}_2}^{-}, \mathscr {N}_{\mathcal {H}_2}^{-} + i \mathscr {B}_{\mathcal {H}_2}^{-} \rangle$$ be BCq-ROFNs. Then,

1. $${\mathcal {H}_1}^{c}\cup {\mathcal {H}_2}^{c}={\left( \mathcal {H}_1\cap \mathcal {H}_2\right) }^{c}$$.
2. $${\mathcal {H}_1}^{c}\cap {\mathcal {H}_2}^{c}=\left( \mathcal {H}_1\cup \mathcal {H}_2\right) ^{c}$$.

### Proof

1.

$$\begin{aligned}{\left( \mathcal {H}_1\cap \mathcal {H}_2\right) }^{c} & =\left\{ \begin{array}{c}\langle \{\min \left\{ \mathscr {M}_{\mathcal {H}_1}^{+}, \mathscr {M}_{\mathcal {H}_2}^{+}\right\} +i \min \left\{ \mathscr {A}_{\mathcal {H}_1}^{+}, \mathscr {A}_{\mathcal {H}_2}^{+}\right\} ,\\ \max \left\{ \mathscr {N}_{\mathcal {H}_1}^{+}, \mathscr {N}_{\mathcal {H}_2}^{+}\right\} +i \max \left\{ \mathscr {B}_{\mathcal {H}_1}^{+}, \mathscr {B}_{\mathcal {H}_2}^{+}\right\} ,\\ \max \left\{ \mathscr {M}_{\mathcal {H}_1}^{-}, \mathscr {M}_{\mathcal {H}_2}^{-}\right\} +i \max \left\{ \mathscr {A}_{\mathcal {H}_1}^{-}, \mathscr {A}_{\mathcal {H}_2}^{-}\right\} ,\\ \min \left\{ \mathscr {N}_{\mathcal {H}_1}^{-}, \mathscr {N}_{\mathcal {H}_2}^{-}\right\} +i \min \left\{ \mathscr {B}_{\mathcal {H}_1}^{-}, \mathscr {B}_ {\mathcal {H}_2}^{-}\right\} \}\rangle ^{c}\end{array}\right\} \\ &=\left\{ \begin{array}{c}\langle \max \{\mathscr {N}_{\mathcal {H}_1}^{+}, \mathscr {N}_{\mathcal {H}_2}^{+}\} +i \max \{\mathscr {B}_{\mathcal {H}_1}^{+}, \mathscr {B}_{\mathcal {H}_2}^{+}\},\\ \min \{\mathscr {M}_{\mathcal {H}_1}^{+}, \mathscr {M}_{\mathcal {H}_2}^{+}\} +i \min \{\mathscr {A}_{\mathcal {H}_1}^{+}, \mathscr {A}_{\mathcal {H}_2}^{+} \},\\ \min \{-|\mathscr {N}_{\mathcal {H}_1}^{-}|, -|\mathscr {N}_{\mathcal {H}_2}^{-}|\} +i \min \{-|\mathscr {B}_{\mathcal {H}_1}^{-}|, -|\mathscr {B}_ {\mathcal {H}_2}^{-}|\},\\ \max \{-|\mathscr {M}_{\mathcal {H}_1}^{-}|, -|\mathscr {M}_{\mathcal {H}_2}^{-}|\} +i \max \{-|\mathscr {A}_{\mathcal {H}_1}^{-}|,-|\mathscr {A}_{\mathcal {H}_{2}}^{-}|\}\rangle \end{array}\right\} \\ & = \langle ({\mathcal{N}}_{{{\mathcal{H}}_{1} }}^{ + } ) + i({\mathcal{B}}_{{{\mathcal{H}}_{1} }}^{ + } ),({\mathcal{M}}_{{{\mathcal{H}}_{1} }}^{ + } ) + i({\mathcal{A}}_{{{\mathcal{H}}_{1} }}^{ + } ), - |{\mathcal{N}}_{{{\mathcal{H}}_{1} }}^{ - } | + i( - |{\mathcal{B}}_{{{\mathcal{H}}_{1} }}^{ - } |), - |{\mathcal{M}}_{{{\mathcal{H}}_{1} }}^{ - } | + i( - |{\mathcal{A}}_{{{\mathcal{H}}_{1} }}^{ - } |)\rangle \\ & \,\,\,\,\,\,\,\,\,\,\,\,\,\,\,\,\,\,\,\,\,\,\,\,\,\,\,\,\,\,\,\,\,\,\,\,\,\,\,\,\,\,\,\,\,\,\,\,\,\,\,\,\,\,\,\,\,\,\,\,\,\,\,\,\,\,\,\,\,\,\,\,\,\,\,\,\,\,\,\,\,\,\, \cup \\ & \langle ({\mathcal{N}}_{{{\mathcal{H}}_{2} }}^{ + } ) + i({\mathcal{B}}_{{{\mathcal{H}}_{2} }}^{ + } ),({\mathcal{M}}_{{{\mathcal{H}}_{2} }}^{ + } ) + i({\mathcal{A}}_{{{\mathcal{H}}_{2} }}^{ + } ), - |{\mathcal{N}}_{{{\mathcal{H}}_{2} }}^{ - } | + i( - |{\mathcal{B}}_{{{\mathcal{H}}_{2} }}^{ - } |), - |{\mathcal{M}}_{{{\mathcal{H}}_{2} }}^{ - } | + i( - |{\mathcal{A}}_{{{\mathcal{H}}_{2} }}^{ - } |)\rangle \\ & = {\mathcal{H}}_{1} ^{c} \cup {\mathcal{H}}_{2} ^{c} = {\mathcal{H}}_{1} ^{c} \cup {\mathcal{H}}_{2} ^{c} .\end{aligned}$$

2. This can be proven similarly to (1).

$$\square$$

### Definition 3.8

Let $$\mathcal {H}$$ be defined as $$\langle \mathscr {M}_{\mathcal {H}}^{+} + i \mathscr {A}_{\mathcal {H}}^{+}, \mathscr {N}_{\mathcal {H}}^{+} + i \mathscr {B}_{\mathcal {H}}^{+}, \mathscr {M}_{\mathcal {H}}^{-} + i \mathscr {A}_{\mathcal {H}}^{-}, \mathscr {N}_{\mathcal {H}}^{-} + i \mathscr {B}_{\mathcal {H}}^{-} \rangle$$, $$\mathcal {H}_1$$ as $$\langle \mathscr {M}_{\mathcal {H}_1}^{+} + i \mathscr {A}_{\mathcal {H}_1}^{+}, \mathscr {N}_{\mathcal {H}_1}^{+} + i \mathscr {B}_{\mathcal {H}_1}^{+}, \mathscr {M}_{\mathcal {H}_1}^{-} + i \mathscr {A}_{\mathcal {H}_1}^{-}, \mathscr {N}_{\mathcal {H}_1}^{-} + i \mathscr {B}_{\mathcal {H}_1}^{-} \rangle$$, and $$\mathcal {H}_2$$ as $$\langle \mathscr {M}_{\mathcal {H}_2}^{+}$$$$+ i \mathscr {A}_{\mathcal {H}_2}^{+},$$$$\mathscr {N}_{\mathcal {H}_2}^{+} +$$$$i \mathscr {B}_{\mathcal {H}_2}^{+},$$$$\mathscr {M}_{\mathcal {H}_2}^{-} + i \mathscr {A}_{\mathcal {H}_2}^{-}, \mathscr {N}_{\mathcal {H}_2}^{-} + i \mathscr {B}_{\mathcal {H}_2}^{-} \rangle$$, where both $$\mathcal {H}, \mathcal {H}_1$$ and $$\mathcal {H}_2$$ are BCq-ROFNs, and let $$\mathscr {I}$$ be a positive real number with $$\mathscr {I} > 0$$. Then, 

1.

4 $$\begin{aligned} \begin{aligned}\mathcal {H}_1 \oplus \mathcal {H}_2 =& \biggl \langle \left( \left( \mathscr {M}_{\mathcal {H}_1}^{+} \right) ^{q} + \left( \mathscr {M}_{\mathcal {H}_2}^{+} \right) ^{q} - \left( \mathscr {M}_{\mathcal {H}_1}^{+} \right) ^{q} \left( \mathscr {M}_{\mathcal {H}_2}^{+} \right) ^{q} \right) ^{\frac{1}{q}}\\&\quad + i \left( \left( \mathscr {A}_{\mathcal {H}_1}^{+} \right) ^{q} + \left( \mathscr {A}_{\mathcal {H}_2}^{+} \right) ^{q} - \left( \mathscr {A}_{\mathcal {H}_1}^{+} \right) ^{q} \left( \mathscr {A}_{\mathcal {H}_2}^{+} \right) ^{q} \right) ^{\frac{1}{q}},\\&\quad \mathscr {N}_{\mathcal {H}_1}^{+} \mathscr {N}_{\mathcal {H}_2}^{+} + i \left( \mathscr {B}_{\mathcal {H}_1}^{+} \mathscr {B}_{\mathcal {H}_2}^{+} \right) , - \left( \mathscr {M}_{\mathcal {H}_1}^{-} \mathscr {M}_{\mathcal {H}_2}^{-} \right) + i \left( - \left( \mathscr {A}_{\mathcal {H}_1}^{-} \mathscr {A}_{\mathcal {H}_2}^{-} \right) \right) ,\\&\quad - \left( \left| \mathscr {N}_{\mathcal {H}_1}^{-} \right| ^{q} + \left| \mathscr {N}_{\mathcal {H}_2}^{-} \right| ^{q} - \left| \mathscr {N}_{\mathcal {H}_1}^{-} \right| ^{q} \left| \mathscr {N}_{\mathcal {H}_2}^{-} \right| ^{q} \right) ^{\frac{1}{q}}\\&\quad + i \left( - \left( \left| \mathscr {B}_{\mathcal {H}_1}^{-} \right| ^{q} + \left| \mathscr {B}_{\mathcal {H}_2}^{-} \right| ^{q} - \left| \mathscr {B}_{\mathcal {H}_1}^{-} \right| ^{q} \left| \mathscr {B}_{\mathcal {H}_2}^{-} \right| ^{q} \right) ^{\frac{1}{q}} \right) \biggr \rangle , \end{aligned} \end{aligned}$$

2.

5 $$\begin{aligned} \begin{aligned}\mathcal {H}_1 \otimes \mathcal {H}_2 =& \biggl \langle \left( \mathscr {M}_{\mathcal {H}_1}^{+} \mathscr {M}_{\mathcal {H}_2}^{+} \right) + i \left( \mathscr {A}_{\mathcal {H}_1}^{+} \mathscr {A}_{\mathcal {H}_2}^{+} \right) ,\\&\quad \left( \left( \mathscr {N}_{\mathcal {H}_1}^{+} \right) ^{q} + \left( \mathscr {N}_{\mathcal {H}_2}^{+} \right) ^{q} - \left( \mathscr {N}_{\mathcal {H}_1}^{+} \right) ^{q} \left( \mathscr {N}_{\mathcal {H}_2}^{+} \right) ^{q} \right) ^{\frac{1}{q}} \\&\quad + i \left( \left( \mathscr {B}_{\mathcal {H}_1}^{+} \right) ^{q} + \left( \mathscr {B}_{\mathcal {H}_2}^{+} \right) ^{q} - \left( \mathscr {B}_{\mathcal {H}_1}^{+} \right) ^{q} \left( \mathscr {B}_{\mathcal {H}_2}^{+} \right) ^{q} \right) ^{\frac{1}{q}},\\&\quad - \left( \left| \mathscr {M}_{\mathcal {H}_1}^{-} \right| ^{q} + \left| \mathscr {M}_{\mathcal {H}_2}^{-} \right| ^{q} - \left| \mathscr {M}_{\mathcal {H}_1}^{-} \right| ^{q} \left| \mathscr {M}_{\mathcal {H}_2}^{-} \right| ^{q} \right) ^{\frac{1}{q}} \\&\quad + i \left( - \left( \left| \mathscr {A}_{\mathcal {H}_1}^{-} \right| ^{q} + \left| \mathscr {A}_{\mathcal {H}_2}^{-} \right| ^{q} - \left| \mathscr {A}_{\mathcal {H}_1}^{-} \right| ^{q} \left| \mathscr {A}_{\mathcal {H}_2}^{-} \right| ^{q} \right) ^{\frac{1}{q}} \right) ,\\&\quad - \left( \mathscr {N}_{\mathcal {H}_1}^{-} \mathscr {N}_{\mathcal {H}_2}^{-} \right) + i \left( - \left( \mathscr {B}_{\mathcal {H}_1}^{-} \mathscr {B}_{\mathcal {H}_2}^{-} \right) \right) \biggr \rangle , \end{aligned} \end{aligned}$$

3.

6 $$\begin{aligned} \begin{aligned}&\mathscr {I} \mathcal {H} = \biggl \langle \left( 1 - \left( 1 - \left( \mathscr {M}_{\mathcal {H}}^{+} \right) ^{q} \right) ^{\mathscr {I}} \right) ^{\frac{1}{q}} + i \left( 1 - \left( 1 - \left( \mathscr {A}_{\mathcal {H}}^{+} \right) ^{q} \right) ^{\mathscr {I}} \right) ^{\frac{1}{q}},\\&\quad \left( \mathscr {N}_{\mathcal {H}}^{+} \right) ^{\mathscr {I}} + i \left( \mathscr {B}_{\mathcal {H}}^{+} \right) ^{\mathscr {I}}, - \left| \mathscr {M}_{\mathcal {H}}^{-} \right| ^{\mathscr {I}} + i \left( - \left| \mathscr {A}_{\mathcal {H}}^{-} \right| ^{\mathscr {I}} \right) ,\\&\quad - \left( 1 - \left( 1 - \left| \mathscr {N}_{\mathcal {H}}^{-} \right| ^{q} \right) ^{\mathscr {I}} \right) ^{\frac{1}{q}} + i \left( - \left( 1 - \left( 1 - \left| \mathscr {B}_{\mathcal {H}}^{-} \right| ^{q} \right) ^{\mathscr {I}} \right) ^{\frac{1}{q}} \right) \biggr \rangle , \end{aligned} \end{aligned}$$

4.

7 $$\begin{aligned} \begin{aligned}&\mathcal {H}^{\mathscr {I}} = \biggl \langle \left( \mathscr {M}_{\mathcal {H}}^{+} \right) ^{\mathscr {I}} + i \left( \mathscr {A}_{\mathcal {H}}^{+} \right) ^{\mathscr {I}},\\&\quad \left( 1 - \left( 1 - \left( \mathscr {N}_{\mathcal {H}}^{+} \right) ^{q} \right) ^{\mathscr {I}} \right) ^{\frac{1}{q}} + i \left( 1 - \left( 1 - \left( \mathscr {B}_{\mathcal {H}}^{+} \right) ^{q} \right) ^{\mathscr {I}} \right) ^{\frac{1}{q}},\\&\quad - \left( 1 - \left( 1 - \left| \mathscr {M}_{\mathcal {H}}^{-} \right| ^{q} \right) ^{\mathscr {I}} \right) ^{\frac{1}{q}} + i \left( - \left( 1 - \left( 1 - \left| \mathscr {A}_{\mathcal {H}}^{-} \right| ^{q} \right) ^{\mathscr {I}} \right) ^{\frac{1}{q}} \right) , \\&\quad - \left| \mathscr {N}_{\mathcal {H}}^{-} \right| ^{\mathscr {I}} + i \left( - \left| \mathscr {B}_{\mathcal {H}}^{-} \right| ^{\mathscr {I}} \right) \biggr \rangle . \end{aligned} \end{aligned}$$

### Example 3.9

We define two BC6-ROFNs as follows:

$$\mathcal {H}_1 = \left\langle .92 + i(.84),.84 + i(.73), -.55 + i(-.92), -.84 + i(-.73) \right\rangle$$

and

$$\mathcal {H}_2 = \left\langle .37 + i(.92),.55 + i(.84), -.92 + i(-.73), -.73 + i(-.92) \right\rangle$$.

Given $\mathscr {I} = 3$, the following results are derived:

1. $$\mathcal {H}_1 \oplus \mathcal {H}_2=$$
  $$\left\langle \left( \left( \mathscr {M}_{\mathcal {H}_1}^{+} \right) ^{q} + \left( \mathscr {M}_{\mathcal {H}_2}^{+} \right) ^{q} - \left( \mathscr {M}_{\mathcal {H}_1}^{+} \right) ^{q} \left( \mathscr {M}_{\mathcal {H}_2}^{+} \right) ^{q} \right) ^{\frac{1}{q}} \right.$$
  $$\left. + i \left( \left( \mathscr {A}_{\mathcal {H}_1}^{+} \right) ^{q} + \left( \mathscr {A}_{\mathcal {H}_2}^{+} \right) ^{q} - \left( \mathscr {A}_{\mathcal {H}_1}^{+} \right) ^{q} \left( \mathscr {A}_{\mathcal {H}_2}^{+} \right) ^{q} \right) ^{\frac{1}{q}}, \right.$$
  $$\left. \mathscr {N}_{\mathcal {H}_1}^{+} \mathscr {N}_{\mathcal {H}_2}^{+} + i \left( \mathscr {B}_{\mathcal {H}_1}^{+} \mathscr {B}_{\mathcal {H}_2}^{+} \right) , - \left( \mathscr {M}_{\mathcal {H}_1}^{-} \mathscr {M}_{\mathcal {H}_2}^{-} \right) + i \left( - \left( \mathscr {A}_{\mathcal {H}_1}^{-} \mathscr {A}_{\mathcal {H}_2}^{-} \right) \right) , \right.$$
  $$\left. - \left( \left| \mathscr {N}_{\mathcal {H}_1}^{-} \right| ^{q} + \left| \mathscr {N}_{\mathcal {H}_2}^{-} \right| ^{q} - \left| \mathscr {N}_{\mathcal {H}_1}^{-} \right| ^{q} \left| \mathscr {N}_{\mathcal {H}_2}^{-} \right| ^{q} \right) ^{\frac{1}{q}} \right.$$
  $$\left. + i \left( - \left( \left| \mathscr {B}_{\mathcal {H}_1}^{-} \right| ^{q} + \left| \mathscr {B}_{\mathcal {H}_2}^{-} \right| ^{q} - \left| \mathscr {B}_{\mathcal {H}_1}^{-} \right| ^{q} \left| \mathscr {B}_{\mathcal {H}_2}^{-} \right| ^{q} \right) ^{\frac{1}{q}} \right) \right\rangle$$
  $$= \left\langle ((.92)^{6} + (.37)^{6} - (.92)^{6} (.37)^{6} )^{\frac{1}{6}} + i ((.84)^{6} + (.92)^{6} - (.84)^{6} (.92)^{6})^{\frac{1}{6}}, \right.$$
  $$\left. (.84) (.55) + i ((.73) (.84)), - ((-.55) (-.92)) + i (-((-.92) (-.73))), \right.$$
  $$\left. -(|-.84|^{6} + |-.73|^{6} - |-.84|^{6} |-.73|^{6})^{\frac{1}{6}} \right.$$
  $$\left. + i (-(|-.73|^{6} + |-.92|^{6} - |-.73|^{6}|-.92|^{6})^{\frac{1}{6}}\right\rangle$$
  $$\approx \langle .9203+.9520 i,.4620+.6132 i, -.5060+(-.6716)i,-.8752+(-.9345)i\rangle$$.
2. $$\mathcal {H}_1 \otimes \mathcal {H}_2=$$
  $$\left\langle \left( \mathscr {M}_{\mathcal {H}_1}^{+} \mathscr {M}_{\mathcal {H}_2}^{+} \right) + i \left( \mathscr {A}_{\mathcal {H}_1}^{+} \mathscr {A}_{\mathcal {H}_2}^{+} \right) , \right.$$
  $$\left. \left( \left( \mathscr {N}_{\mathcal {H}_1}^{+} \right) ^{q} + \left( \mathscr {N}_{\mathcal {H}_2}^{+} \right) ^{q} - \left( \mathscr {N}_{\mathcal {H}_1}^{+} \right) ^{q} \left( \mathscr {N}_{\mathcal {H}_2}^{+} \right) ^{q} \right) ^{\frac{1}{q}} \right.$$
  $$\left. + i \left( \left( \mathscr {B}_{\mathcal {H}_1}^{+} \right) ^{q} + \left( \mathscr {B}_{\mathcal {H}_2}^{+} \right) ^{q} - \left( \mathscr {B}_{\mathcal {H}_1}^{+} \right) ^{q} \left( \mathscr {B}_{\mathcal {H}_2}^{+} \right) ^{q} \right) ^{\frac{1}{q}}, \right.$$
  $$\left. - \left( \left| \mathscr {M}_{\mathcal {H}_1}^{-} \right| ^{q} + \left| \mathscr {M}_{\mathcal {H}_2}^{-} \right| ^{q} - \left| \mathscr {M}_{\mathcal {H}_1}^{-} \right| ^{q} \left| \mathscr {M}_{\mathcal {H}_2}^{-} \right| ^{q} \right) ^{\frac{1}{q}} \right.$$
  $$\left. + i \left( - \left( \left| \mathscr {A}_{\mathcal {H}_1}^{-} \right| ^{q} + \left| \mathscr {A}_{\mathcal {H}_2}^{-} \right| ^{q} - \left| \mathscr {A}_{\mathcal {H}_1}^{-} \right| ^{q} \left| \mathscr {A}_{\mathcal {H}_2}^{-} \right| ^{q} \right) ^{\frac{1}{q}} \right) , \right.$$
  $$\left. - \left( \mathscr {N}_{\mathcal {H}_1}^{-} \mathscr {N}_{\mathcal {H}_2}^{-} \right) + i \left( - \left( \mathscr {B}_{\mathcal {H}_1}^{-} \mathscr {B}_{\mathcal {H}_2}^{-} \right) \right) \right\rangle$$
  $$=\left\langle ((.92) (.37)) + i ((.84) (.92)), \right.$$
  $$\left. ((.84)^{6} + (.55)^{6} - ((.84)^{6} (.55)^{6}))^{\frac{1}{6}} + i ((.73)^{6} + (.84)^{6} - ((.73)^{6} (.84)^{6}))^{\frac{1}{6}}, \right.$$
  $$\left. - (|-.55|^{6} + |-.92|^{6} - |-.55|^{6} |-.92|^{6})^{\frac{1}{6}} \right.$$
  $$\left. + i (-(|-.92|^{6} + |-.73|^{6} - | -.92|^{6} |-.73|^{6})^{\frac{1}{6}}), \right.$$
  $$\left. - ((-.84) (-.73)) + i (-((-.73) (-.92))) \right\rangle$$
  $$\approx \langle .3404+.7728 i,.8470+.8752 i, -.9227+(-.9345)i,-.6132+(-.6716)i\rangle$$.
3. $$3\mathcal {H}_{1}=$$
  $$\left\langle (1 -(1 - (.92)^{6})^{3})^{\frac{1}{6}} + i (1 - (1 - (.84)^{6})^{3})^{\frac{1}{6}}, \right.$$
  $$\left. (.84)^{3} + i (.73)^{3}, \right.$$
  $$\left. - |-.55|^{3} + i (-|-.92|^{3}), \right.$$
  $$\left. - (1 - (1 - |-.84|^{6})^{3})^{\frac{1}{6}} + i (-(1 - (1 - |-.73|^{6})^{3})^{\frac{1}{6}}) \right\rangle$$
  $$\approx \langle .9896+.9483i,.5927+.3890i, -.1664+(-.7787)i,-.9483+(-.8543)i\rangle$$.
4. $$\mathcal {H}_{1}^{3}=$$
  $$\left\langle (.92)^{3} + i (.84)^{3}, \right.$$
  $$\left. (1 - (1 - (.84)^{6})^{3})^{\frac{1}{6}} + i (1 - (1 - (.73)^{6})^{3})^{\frac{1}{6}}, \right.$$
  $$\left. - (1 - (1 - |-.55|^{6})^{3})^{\frac{1}{6}} + i (-(1 - (1 - |-.92|^{6})^{3})^{\frac{1}{6}}), \right.$$
  $$\left. - |-.84|^{3} + i (- |-.73|^{3}) \right\rangle$$
  $$\approx \langle .7787+.5927i,.9483+.8543i, -.6575+(-.9896)i,-.5927+(-.3890)i\rangle$$.

### Theorem 3.10

Consider two BCq-ROFNs $\mathcal {H}_1$ and $\mathcal {H}_2$ defined as follows:

$$\mathcal {H}_1 = \left\langle \mathscr {M}_{\mathcal {H}_1}^{+} + i \mathscr {A}_{\mathcal {H}_1}^{+}, \mathscr {N}_{\mathcal {H}_1}^{+} + i \mathscr {B}_{\mathcal {H}_1}^{+}, \mathscr {M}_{\mathcal {H}_1}^{-} + i \mathscr {A}_{\mathcal {H}_1}^{-}, \mathscr {N}_{\mathcal {H}_1}^{-} + i \mathscr {B}_{\mathcal {H}_1}^{-} \right\rangle$$

and

$$\mathcal {H}_2 = \left\langle \mathscr {M}_{\mathcal {H}_2}^{+} + i \mathscr {A}_{\mathcal {H}_2}^{+}, \mathscr {N}_{\mathcal {H}_2}^{+} + i \mathscr {B}_{\mathcal {H}_2}^{+}, \mathscr {M}_{\mathcal {H}_2}^{-} + i \mathscr {A}_{\mathcal {H}_2}^{-}, \mathscr {N}_{\mathcal {H}_2}^{-} + i \mathscr {B}_{\mathcal {H}_2}^{-} \right\rangle .$$

Then, it follows that both $\mathcal {H}_1 \oplus \mathcal {H}_2$ and $\mathcal {H}_1 \otimes \mathcal {H}_2$ are also BCq-ROFNs.

### Proof

For the two BCq-ROFNs, $\mathcal {H}_1$ and $\mathcal {H}_2$, the following inequalities hold:

$$\begin{aligned} &0 \le \left( \mathscr {M}_{\mathcal {H}_1}^{+}\right) ^{q} \le 1, \quad 0 \le \left( \mathscr {N}_{\mathcal {H}_1}^{+}\right) ^{q} \le 1, \quad 0 \le \left( \mathscr {M}_{\mathcal {H}_1}^{+}\right) ^{q} + \left( \mathscr {N}_{\mathcal {H}_1}^{+}\right) ^{q} \le 1, \\& 0 \le \left| \mathscr {M}_{\mathcal {H}_1}^{-}\right| ^{q} \le 1, \quad 0 \le \left| \mathscr {N}_{\mathcal {H}_1}^{-}\right| ^{q} \le 1, \quad 0 \le \left| \mathscr {M}_{\mathcal {H}_1}^{-}\right| ^{q} + \left| \mathscr {N}_{\mathcal {H}_1}^{-}\right| ^{q} \le 1, \\& 0 \le \left( \mathscr {M}_{\mathcal {H}_2}^{+}\right) ^{q} \le 1, \quad 0 \le \left( \mathscr {N}_{\mathcal {H}_2}^{+}\right) ^{q} \le 1, \quad 0 \le \left( \mathscr {M}_{\mathcal {H}_2}^{+}\right) ^{q} + \left( \mathscr {N}_{\mathcal {H}_2}^{+}\right) ^{q} \le 1, \\& 0 \le \left| \mathscr {M}_{\mathcal {H}_2}^{-}\right| ^{q} \le 1, \quad 0 \le \left| \mathscr {N}_{\mathcal {H}_2}^{-}\right| ^{q} \le 1, \quad \text {and} \quad 0 \le \left| \mathscr {M}_{\mathcal {H}_2}^{-}\right| ^{q} + \left| \mathscr {N}_{\mathcal {H}_2}^{-}\right| ^{q} \le 1. \end{aligned}$$

Moreover, given that:

$$\begin{aligned}&0 \le \mathscr {A}_{\mathcal {H}_1}^{+} \le 1, \quad 0 \le \mathscr {A}_{\mathcal {H}_2}^{+} \le 1, \quad 0 \le \mathscr {B}_{\mathcal {H}_1}^{+} \le 1, \quad 0 \le \mathscr {B}_{\mathcal {H}_2}^{+} \le 1, \\& 0 \le (\mathscr {A}_{\mathcal {H}_1}^{+})^{q} + (\mathscr {B}_{\mathcal {H}_1}^{+})^{q} \le 1, \quad 0 \le (\mathscr {A}_{\mathcal {H}_2}^{+})^{q} + (\mathscr {B}_{\mathcal {H}_2}^{+})^{q} \le 1, \\& 0 \le |\mathscr {B}_{\mathcal {H}_1}^{-}| \le 1, \quad 0 \le |\mathscr {B}_{\mathcal {H}_2}^{-}| \le 1, \quad 0 \le |\mathscr {A}_{\mathcal {H}_1}^{-}| \le 1, \quad 0 \le |\mathscr {A}_{\mathcal {H}_2}^{-}| \le 1, \\& 0 \le |\mathscr {A}_{\mathcal {H}_1}^{-}|^{q} + |\mathscr {B}_{\mathcal {H}_1}^{-}|^{q} \le 1, \quad \text {and} \quad 0 \le |\mathscr {A}_{\mathcal {H}_2}^{-}|^{q} + |\mathscr {B}_{\mathcal {H}_2}^{-}|^{q} \le 1. \end{aligned}$$

These inequalities establish the necessary constraints on the parameters of the BCq-ROFNs. Then, we obtain the following inequalities:

$$\begin{aligned}&\left( \mathscr {M}_{\mathcal {H}_1}^{+}\right) ^{q} \ge \left( \mathscr {M}_{\mathcal {H}_1}^{+}\right) ^{q} \left( \mathscr {M}_{\mathcal {H}_2}^{+}\right) ^{q}, \quad \left( \mathscr {M}_{\mathcal {H}_2}^{+}\right) ^{q} \ge \left( \mathscr {M}_{\mathcal {H}_1}^{+}\right) ^{q} \left( \mathscr {M}_{\mathcal {H}_2}^{+}\right) ^{q}, \\&\quad 1 \ge \left( \mathscr {M}_{\mathcal {H}_1}^{+}\right) ^{q} \left( \mathscr {M}_{\mathcal {H}_2}^{+}\right) ^{q} \ge 0, \quad \left( \mathscr {N}_{\mathcal {H}_1}^{+}\right) ^{q} \ge \left( \mathscr {N}_{\mathcal {H}_1}^{+}\right) ^{q} \left( \mathscr {N}_{\mathcal {H}_2}^{+}\right) ^{q}, \\&\quad \left( \mathscr {N}_{\mathcal {H}_2}^{+}\right) ^{q} \ge \left( \mathscr {N}_{\mathcal {H}_1}^{+}\right) ^{q} \left( \mathscr {N}_{\mathcal {H}_2}^{+}\right) ^{q}, \quad \text {and} \quad 1 \ge \left( \mathscr {N}_{\mathcal {H}_1}^{+}\right) ^{q} \left( \mathscr {N}_{\mathcal {H}_2}^{+}\right) ^{q} \ge . \end{aligned}$$

These relationships imply that:

$$\begin{aligned} \left( \mathscr {M}_{\mathcal {H}_1}^{+}\right) ^{q} + \left( \mathscr {M}_{\mathcal {H}_2}^{+}\right) ^{q} - \left( \mathscr {M}_{\mathcal {H}_1}^{+}\right) ^{q} \left( \mathscr {M}_{\mathcal {H}_2}^{+}\right) ^{q} \ge 0 \end{aligned}$$

which further leads to:

$$\begin{aligned} \left( \left( \mathscr {M}_{\mathcal {H}_1}^{+}\right) ^{q} + \left( \mathscr {M}_{\mathcal {H}_2}^{+}\right) ^{q} - \left( \mathscr {M}_{\mathcal {H}_1}^{+}\right) ^{q} \left( \mathscr {M}_{\mathcal {H}_2}^{+}\right) ^{q}\right) ^{\frac{1}{q}} \ge . \end{aligned}$$

Similarly, we have:

$$\begin{aligned} \left( \mathscr {N}_{\mathcal {H}_1}^{+}\right) ^{q} + \left( \mathscr {N}_{\mathcal {H}_2}^{+}\right) ^{q} - \left( \mathscr {N}_{\mathcal {H}_1}^{+}\right) ^{q} \left( \mathscr {N}_{\mathcal {H}_2}^{+}\right) ^{q} \ge 0, \end{aligned}$$

which implies:

$$\begin{aligned} \left( \left( \mathscr {N}_{\mathcal {H}_1}^{+}\right) ^{q} + \left( \mathscr {N}_{\mathcal {H}_2}^{+}\right) ^{q} - \left( \mathscr {N}_{\mathcal {H}_1}^{+}\right) ^{q} \left( \mathscr {N}_{\mathcal {H}_2}^{+}\right) ^{q}\right) ^{\frac{1}{q}} \ge . \end{aligned}$$

Given that

$$\left( \mathscr {M}_{\mathcal {H}_2}^{+}\right) ^{q} \le 1 \quad \text {and} \quad 0 \le 1-\left( \mathscr {M}_{\mathcal {H}_1}^{+}\right) ^{q},$$

we obtain the inequality

$$\left( \mathscr {M}_{\mathcal {H}_2}^{+}\right) ^{q} \left( 1 - \left( \mathscr {M}_{\mathcal {H}_1}^{+}\right) ^{q} \right) \le 1 - \left( \mathscr {M}_{\mathcal {H}_1}^{+}\right) ^{q}.$$

From this, it follows that

$$\left( \mathscr {M}_{\mathcal {H}_1}^{+}\right) ^{q} + \left( \mathscr {M}_{\mathcal {H}_2}^{+}\right) ^{q} - \left( \mathscr {M}_{\mathcal {H}_1}^{+}\right) ^{q} \left( \mathscr {M}_{\mathcal {H}_2}^{+}\right) ^{q} \le 1.$$

Taking the $q$th root on both sides gives

$$\left( \left( \mathscr {M}_{\mathcal {H}_1}^{+}\right) ^{q} + \left( \mathscr {M}_{\mathcal {H}_2}^{+}\right) ^{q} - \left( \mathscr {M}_{\mathcal {H}_1}^{+}\right) ^{q} \left( \mathscr {M}_{\mathcal {H}_2}^{+}\right) ^{q} \right) ^{\frac{1}{q}} \le 1.$$

By applying the same reasoning, we derive a similar bound:

$$\left( \left( \mathscr {N}_{\mathcal {H}_1}^{+}\right) ^{q} + \left( \mathscr {N}_{\mathcal {H}_2}^{+}\right) ^{q} - \left( \mathscr {N}_{\mathcal {H}_1}^{+}\right) ^{q} \left( \mathscr {N}_{\mathcal {H}_2}^{+}\right) ^{q} \right) ^{\frac{1}{q}} \le 1.$$

Furthermore, it is evident that

$$0 \le \left( \mathscr {N}_{\mathcal {H}_1}^{+}\right) ^{q} \le 1 - \left( \mathscr {M}_{\mathcal {H}_1}^{+}\right) ^{q}, \quad 0 \le \left( \mathscr {N}_{\mathcal {H}_2}^{+}\right) ^{q} \le 1 - \left( \mathscr {M}_{\mathcal {H}_2}^{+}\right) ^{q}.$$

Using these bounds, we establish the inequality

$$\begin{aligned}&\quad \left( \left( \mathscr {M}_{\mathcal {H}_1}^{+}\right) ^{q} + \left( \mathscr {M}_{\mathcal {H}_2}^{+}\right) ^{q} - \left( \mathscr {M}_{\mathcal {H}_1}^{+}\right) ^{q} \left( \mathscr {M}_{\mathcal {H}_2}^{+}\right) ^{q} \right) ^{\frac{1}{q}} + \left( \mathscr {N}_{\mathcal {H}_1}^{+} \mathscr {N}_{\mathcal {H}_2}^{+} \right) ^{q} \\&\le \left( \mathscr {M}_{\mathcal {H}_1}^{+}\right) ^{q} + \left( \mathscr {M}_{\mathcal {H}_2}^{+}\right) ^{q} - \left( \mathscr {M}_{\mathcal {H}_1}^{+}\right) ^{q} \left( \mathscr {M}_{\mathcal {H}_2}^{+}\right) ^{q} + \left( 1 - \left( \mathscr {M}_{\mathcal {H}_1}^{+}\right) ^{q} \right) \left( 1 - \left( \mathscr {M}_{\mathcal {H}_2}^{+}\right) ^{q} \right) = 1. \end{aligned}$$

Thus, we conclude that

$$0 \le \left( \left( \mathscr {M}_{\mathcal {H}_1}^{+}\right) ^{q} + \left( \mathscr {M}_{\mathcal {H}_2}^{+}\right) ^{q} - \left( \mathscr {M}_{\mathcal {H}_1}^{+}\right) ^{q} \left( \mathscr {M}_{\mathcal {H}_2}^{+}\right) ^{q} \right) ^{\frac{1}{q}} \le 1,$$

$$0 \le \mathscr {N}_{\mathcal {H}_1}^{+} \mathscr {N}_{\mathcal {H}_2}^{+} \le 1,$$

and

$$0 \le \left( \left( \left( \mathscr {M}_{\mathcal {H}_1}^{+}\right) ^{q} + \left( \mathscr {M}_{\mathcal {H}_2}^{+}\right) ^{q} - \left( \mathscr {M}_{\mathcal {H}_1}^{+}\right) ^{q} \left( \mathscr {M}_{\mathcal {H}_2}^{+}\right) ^{q} \right) ^{\frac{1}{q}} \right) ^{q} + \left( \mathscr {N}_{\mathcal {H}_1}^{+} \mathscr {N}_{\mathcal {H}_2}^{+} \right) ^{q} \le 1.$$

Similarly, we obtain the following inequalities: 

1.

$$\begin{aligned}&0 \le \mathscr {M}_{\mathcal {H}_1}^{+} \mathscr {M}_{\mathcal {H}_2}^{+} \le 1, \\&0 \le \left( \left( \mathscr {N}_{\mathcal {H}_1}^{+}\right) ^{q} + \left( \mathscr {N}_{\mathcal {H}_2}^{+}\right) ^{q} - \left( \mathscr {N}_{\mathcal {H}_1}^{+}\right) ^{q} \left( \mathscr {N}_{\mathcal {H}_2}^{+}\right) ^{q} \right) ^{\frac{1}{q}} \le 1, \\ 0&\le \left( \mathscr {M}_{\mathcal {H}_1}^{+} \mathscr {M}_{\mathcal {H}_2}^{+} \right) ^{q} + \left( \left( \left( \mathscr {N}_{\mathcal {H}_1}^{+}\right) ^{q} + \left( \mathscr {N}_{\mathcal {H}_2}^{+}\right) ^{q} - \left( \mathscr {N}_{\mathcal {H}_1}^{+}\right) ^{q} \left( \mathscr {N}_{\mathcal {H}_2}^{+}\right) ^{q} \right) ^{\frac{1}{q}} \right) ^{q} \le 1. \end{aligned}$$

2.

$$\begin{aligned} -1&\le -\mathscr {M}_{\mathcal {H}_1}^{-} \mathscr {M}_{\mathcal {H}_2}^{-} \le 0, \\ -1&\le -\left( \left| \mathscr {N}_{\mathcal {H}_1}^{-} \right| ^{q} + \left| \mathscr {N}_{\mathcal {H}_2}^{-} \right| ^{q} - \left| \mathscr {N}_{\mathcal {H}_1}^{-} \right| ^{q} \left| \mathscr {N}_{\mathcal {H}_2}^{-} \right| ^{q} \right) ^{\frac{1}{q}} \le 0, \\ 0&\le \left| -\mathscr {M}_{\mathcal {H}_1}^{-} \mathscr {M}_{\mathcal {H}_2}^{-} \right| ^{q} + \left| -\left( \left| \mathscr {N}_{\mathcal {H}_1}^{-} \right| ^{q} + \left| \mathscr {N}_{\mathcal {H}_2}^{-} \right| ^{q} - \left| \mathscr {N}_{\mathcal {H}_1}^{-} \right| ^{q} \left| \mathscr {N}_{\mathcal {H}_2}^{-} \right| ^{q} \right) ^{\frac{1}{q}} \right| ^{q} \le 1. \end{aligned}$$

3.

$$\begin{aligned} -1&\le -\left( \left| \mathscr {M}_{\mathcal {H}_1}^{-} \right| ^{q} + \left| \mathscr {M}_{\mathcal {H}_2}^{-} \right| ^{q} - \left| \mathscr {M}_{\mathcal {H}_1}^{-} \right| ^{q} \left| \mathscr {M}_{\mathcal {H}_2}^{-} \right| ^{q} \right) ^{\frac{1}{q}} \le 0, \\ -1&\le -\mathscr {N}_{\mathcal {H}_1}^{-} \mathscr {N}_{\mathcal {H}_2}^{-} \le 0, \\ 0&\le \left| -\left( \left| \mathscr {M}_{\mathcal {H}_1}^{-} \right| ^{q} + \left| \mathscr {M}_{\mathcal {H}_2}^{-} \right| ^{q} - \left| \mathscr {M}_{\mathcal {H}_1}^{-} \right| ^{q} \left| \mathscr {M}_{\mathcal {H}_2}^{-} \right| ^{q} \right) ^{\frac{1}{q}} \right| ^{q} + \left| -\mathscr {N}_{\mathcal {H}_1}^{-} \mathscr {N}_{\mathcal {H}_2}^{-} \right| ^{q} \le 1. \end{aligned}$$

Following the same approach, we derive:

- (i)
  $$0 \le \left( \left( \mathscr {A}_{\mathcal {H}_1}^{+} \right) ^{q} + \left( \mathscr {A}_{\mathcal {H}_2}^{+} \right) ^{q} - \left( \mathscr {A}_{\mathcal {H}_1}^{+} \right) ^{q} \left( \mathscr {A}_{\mathcal {H}_2}^{+} \right) ^{q} \right) ^{\frac{1}{q}} \le 1, \quad 0 \le \left( \mathscr {B}_{\mathcal {H}_1}^{+} \right) \left( \mathscr {B}_{\mathcal {H}_2}^{+} \right) \le 1,$$
  and
  $$0 \le \left( \left( \left( \mathscr {A}_{\mathcal {H}_1}^{+} \right) ^{q} + \left( \mathscr {A}_{\mathcal {H}_2}^{+} \right) ^{q} - \left( \mathscr {A}_{\mathcal {H}_1}^{+} \right) ^{q} \left( \mathscr {A}_{\mathcal {H}_2}^{+} \right) ^{q} \right) ^{\frac{1}{q}} \right) ^{q} + \left( \left( \mathscr {B}_{\mathcal {H}_1}^{+} \right) \left( \mathscr {B}_{\mathcal {H}_2}^{+} \right) \right) ^{q} \le 1.$$
- (ii)
  $$0 \le \left| - \left( \left| \mathscr {B}_{\mathcal {H}_1}^{-} \right| ^{q} + \left| \mathscr {B}_{\mathcal {H}_2}^{-} \right| ^{q} - \left| \mathscr {B}_{\mathcal {H}_1}^{-} \right| ^{q} \left| \mathscr {B}_{\mathcal {H}_2}^{-} \right| ^{q} \right) \right| ^{\frac{1}{q}} \le 1, \quad 0 \le \left| \mathscr {A}_{\mathcal {H}_1}^{-} \right| \left| \mathscr {A}_{\mathcal {H}_2}^{-} \right| \le 1,$$
  and
  $$0 \le \left| - \left( \left| \mathscr {B}_{\mathcal {H}_1}^{-} \right| ^{q} + \left| \mathscr {B}_{\mathcal {H}_2}^{-} \right| ^{q} - \left| \mathscr {B}_{\mathcal {H}_1}^{-} \right| ^{q} \left| \mathscr {B}_{\mathcal {H}_2}^{-} \right| ^{q} \right) ^{\frac{1}{q}} \right| ^{q} + \left( \left| \mathscr {A}_{\mathcal {H}_1}^{-} \right| \left| \mathscr {A}_{\mathcal {H}_2}^{-} \right| \right) ^{q} \le 1.$$
- (iii)
  $$0 \le \left| - \left( \left| \mathscr {A}_{\mathcal {H}_1}^{-} \right| ^{q} + \left| \mathscr {A}_{\mathcal {H}_2}^{-} \right| ^{q} - \left| \mathscr {A}_{\mathcal {H}_1}^{-} \right| ^{q} \left| \mathscr {A}_{\mathcal {H}_2}^{-} \right| ^{q} \right) \right| ^{\frac{1}{q}} \le 1, \quad 0 \le \left| \mathscr {B}_{\mathcal {H}_1}^{-} \right| \left| \mathscr {B}_{\mathcal {H}_2}^{-} \right| \le 1,$$
  and
  $$0 \le \left| - \left( \left| \mathscr {A}_{\mathcal {H}_1}^{-} \right| ^{q} + \left| \mathscr {A}_{\mathcal {H}_2}^{-} \right| ^{q} - \left| \mathscr {A}_{\mathcal {H}_1}^{-} \right| ^{q} \left| \mathscr {A}_{\mathcal {H}_2}^{-} \right| ^{q} \right) ^{\frac{1}{q}} \right| ^{q} + \left( \left| \mathscr {B}_{\mathcal {H}_1}^{-} \right| \left| \mathscr {B}_{\mathcal {H}_2}^{-} \right| \right) ^{q} \le 1.$$
- (iv)
  $$0 \le \left( \left( \mathscr {B}_{\mathcal {H}_1}^{+} \right) ^{q} + \left( \mathscr {B}_{\mathcal {H}_2}^{+} \right) ^{q} - \left( \mathscr {B}_{\mathcal {H}_1}^{+} \right) ^{q} \left( \mathscr {B}_{\mathcal {H}_2}^{+} \right) ^{q} \right) ^{\frac{1}{q}} \le 1, \quad 0 \le \left( \mathscr {A}_{\mathcal {H}_1}^{+} \right) \left( \mathscr {A}_{\mathcal {H}_2}^{+} \right) \le 1,$$
  and
  $$0 \le \left( \left( \left( \mathscr {B}_{\mathcal {H}_1}^{+} \right) ^{q} + \left( \mathscr {B}_{\mathcal {H}_2}^{+} \right) ^{q} - \left( \mathscr {B}_{\mathcal {H}_1}^{+} \right) ^{q} \left( \mathscr {B}_{\mathcal {H}_2}^{+} \right) ^{q} \right) ^{\frac{1}{q}} \right) ^{q} + \left( \left( \mathscr {A}_{\mathcal {H}_1}^{+} \right) \left( \mathscr {A}_{\mathcal {H}_2}^{+} \right) \right) ^{q} \le 1.$$

Therefore, it follows that $\mathcal {H}_1 \oplus \mathcal {H}_2$ and $\mathcal {H}_1 \otimes \mathcal {H}_2$ satisfy the conditions for being BCq-ROFNs. $\square$

### Theorem 3.11

Assume that $\mathcal {H} = \langle \mathscr {M}_{\mathcal {H}}^{+} + i \mathscr {A}_{\mathcal {H}}^{+}, \mathscr {N}_{\mathcal {H}}^{+} + i \mathscr {B}_{\mathcal {H}}^{+}, \mathscr {M}_{\mathcal {H}}^{-} + i \mathscr {A}_{\mathcal {H}}^{-}, \mathscr {N}_{\mathcal {H}}^{-} + i \mathscr {B}_{\mathcal {H}}^{-} \rangle$ is a BCq-ROFN, and $\mathscr {I}>0$. Then, both $\mathscr {I}\mathcal {H}$ and $\mathcal {H}^{\mathscr {I}}$ are also BCq-ROFNs.

### Proof

We start by noting the following inequalities:

$$0 \le \left( \mathscr {M}_{\mathcal {H}}^{+} \right) ^{q} \le 1, \quad 0 \le \left( \mathscr {N}_{\mathcal {H}}^{+} \right) ^{q} \le 1, \quad 0 \le \left( \mathscr {M}_{\mathcal {H}}^{+} \right) ^{q} + \left( \mathscr {N}_{\mathcal {H}}^{+} \right) ^{q} \le 1,$$

$$0 \le \left| \mathscr {M}_{\mathcal {H}}^{-} \right| ^{q} \le 1, \quad 0 \le \left| \mathscr {N}_{\mathcal {H}}^{-} \right| ^{q} \le 1, \quad 0 \le \left| \mathscr {M}_{\mathcal {H}}^{-} \right| ^{q} + \left| \mathscr {N}_{\mathcal {H}}^{-} \right| ^{q} \le 1.$$

This implies the following bounds:

$$0 \le \left( \mathscr {N}_{\mathcal {H}}^{+} \right) ^{q} \le 1 - \left( \mathscr {M}_{\mathcal {H}}^{+} \right) ^{q}, \quad 0 \le \left| \mathscr {M}_{\mathcal {H}}^{-} \right| ^{q} \le 1 - \left| \mathscr {N}_{\mathcal {H}}^{-} \right| ^{q}.$$

Thus, we can conclude:

$$0 \le \left( 1 - \left( \mathscr {M}_{\mathcal {H}}^{+} \right) ^{q} \right) ^{\mathscr {I}}, \quad 0 \le \left( 1 - \left| \mathscr {N}_{\mathcal {H}}^{-} \right| ^{q} \right) ^{\mathscr {I}}.$$

Next, we apply the following relations:

$$1 - \left( 1 - \left( \mathscr {M}_{\mathcal {H}}^{+} \right) ^{q} \right) ^{\mathscr {I}} \le 1, \quad 1 - \left( 1 - \left| \mathscr {N}_{\mathcal {H}}^{-} \right| ^{q} \right) ^{\mathscr {I}} \le 1.$$

This leads to the bounds:

$$0 \le \left( 1 - \left( 1 - \left( \mathscr {M}_{\mathcal {H}}^{+} \right) ^{q} \right) ^{\mathscr {I}} \right) ^{\frac{1}{q}} \le 1, \quad 0 \le \left( 1 - \left( 1 - \left| \mathscr {N}_{\mathcal {H}}^{-} \right| ^{q} \right) ^{\mathscr {I}} \right) ^{\frac{1}{q}} \le 1.$$

We also observe that:

$$0 \le \left( \mathscr {N}_{\mathcal {H}}^{+} \right) ^{\mathscr {I}} \le 1, \quad -1 \le - \left| \mathscr {M}_{\mathcal {H}}^{-} \right| ^{\mathscr {I}} \le .$$

From this, we deduce:

$$0 \le \left( \left( 1 - \left( 1 - \left( \mathscr {M}_{\mathcal {H}}^{+} \right) ^{q} \right) ^{\mathscr {I}} \right) ^{\frac{1}{q}} \right) ^{q} + \left( \left( \mathscr {N}_{\mathcal {H}}^{+} \right) ^{\mathscr {I}} \right) ^{q} \le 1,$$

and

$$0 \le \left| - \left| \mathscr {M}_{\mathcal {H}}^{-} \right| ^{\mathscr {I}} \right| ^{q} + \left( 1 - \left( 1 - \left| \mathscr {N}_{\mathcal {H}}^{-} \right| ^{q} \right) ^{\mathscr {I}} \right) ^{\frac{1}{q}} \le 1.$$

Next, we consider similar relations for other terms:

1. $$0 \le \left( 1 - \left( 1 - \left( \mathscr {A}_{\mathcal {H}}^{+} \right) ^{q} \right) ^{\mathscr {I}} \right) ^{\frac{1}{q}} \le 1, \quad 0 \le \left( \mathscr {B}_{\mathcal {H}}^{+} \right) ^{\mathscr {I}} \le 1,$$
  and
  $$0 \le \left( \left( 1 - \left( 1 - \left( \mathscr {A}_{\mathcal {H}}^{+} \right) ^{q} \right) ^{\mathscr {I}} \right) ^{\frac{1}{q}} \right) ^{q} + \left( \left( \mathscr {B}_{\mathcal {H}}^{+} \right) ^{\mathscr {I}} \right) ^{q} \le 1.$$
2. $$0 \le \left| \mathscr {A}_{\mathcal {H}}^{-} \right| ^{\mathscr {I}} \le 1, \quad -1 \le - \left( 1 - \left( 1 - \left| \mathscr {B}_{\mathcal {H}}^{-} \right| ^{q} \right) ^{\mathscr {I}} \right) ^{\frac{1}{q}} \le 0,$$
  and
  $$0 \le \left| - \left| \mathscr {A}_{\mathcal {H}}^{-} \right| ^{\mathscr {I}} \right| ^{q} + \left| - \left( 1 - \left( 1 - \left| \mathscr {B}_{\mathcal {H}}^{-} \right| ^{q} \right) ^{\mathscr {I}} \right) ^{\frac{1}{q}} \right| ^{q} \le 1.$$

Similarly, we obtain the following relations:

$$0 \le \left( \left( \mathscr {M}_{\mathcal {H}}^{+} \right) ^{\mathscr {I}} \right) ^{q} + \left( \left( 1 - \left( 1 - \left( \mathscr {N}_{\mathcal {H}}^{+} \right) ^{q} \right) ^{\mathscr {I}} \right) ^{\frac{1}{q}} \right) ^{q} \le 1,$$

and

$$0 \le \left| - \left( 1 - \left( 1 - \left| \mathscr {M}_{\mathcal {H}}^{-} \right| ^{q} \right) ^{\mathscr {I}} \right) ^{\frac{1}{q}} \right| ^{q} + \left| -\left| \mathscr {N}_{\mathcal {H}}^{-} \right| ^{\mathscr {I}} \right| ^{q} \le 1.$$

Finally, from the above, we conclude: 

1.

$$0 \le \left( \mathscr {A}_{\mathcal {H}}^{+} \right) ^{\mathscr {I}} \le 1, \quad 0 \le \left( 1 - \left( 1 - \left( \mathscr {B}_{\mathcal {H}}^{+} \right) ^{q} \right) ^{\mathscr {I}} \right) ^{\frac{1}{q}} \le 1,$$

and

$$0 \le \left( \left( \mathscr {A}_{\mathcal {H}}^{+} \right) ^{\mathscr {I}} \right) ^{q} + \left( \left( 1 - \left( 1 - \left( \mathscr {B}_{\mathcal {H}}^{+} \right) ^{q} \right) ^{\mathscr {I}} \right) ^{\frac{1}{q}} \right) ^{q} \le 1.$$

2.

$$-1 \le - \left( 1 - \left( 1 - \left| \mathscr {A}_{\mathcal {H}}^{-} \right| ^{q} \right) ^{\mathscr {I}} \right) ^{\frac{1}{q}} \le 0, \quad 0 \le \left| \mathscr {B}_{\mathcal {H}}^{-} \right| ^{\mathscr {I}} \le 1,$$

and

$$0 \le \left| - \left( 1 - \left( 1 - \left| \mathscr {A}_{\mathcal {H}}^{-} \right| ^{q} \right) ^{\mathscr {I}} \right) ^{\frac{1}{q}} \right| ^{q} + \left| - \left| \mathscr {B}_{\mathcal {H}}^{-} \right| ^{\mathscr {I}} \right| ^{q} \le 1.$$

Thus, it follows that both $\mathscr {I} \mathcal {H}$ and $\mathcal {H}^{\mathscr {I}}$ are BCq-ROFNs. $\square$

### Theorem 3.12

Consider the BCq-ROFNs given by

$$\mathcal {H} = \left\langle \mathscr {M}_{\mathcal {H}}^{+} + i \mathscr {A}_{\mathcal {H}}^{+}, \mathscr {N}_{\mathcal {H}}^{+} + i \mathscr {B}_{\mathcal {H}}^{+}, \mathscr {M}_{\mathcal {H}}^{-} + i \mathscr {A}_{\mathcal {H}}^{-}, \mathscr {N}_{\mathcal {H}}^{-} + i \mathscr {B}_{\mathcal {H}}^{-} \right\rangle ,$$

$$\mathcal {H}_1 = \left\langle \mathscr {M}_{\mathcal {H}_{1}}^{+} + i \mathscr {A}_{\mathcal {H}_{1}}^{+}, \mathscr {N}_{\mathcal {H}_{1}}^{+} + i \mathscr {B}_{\mathcal {H}_{1}}^{+}, \mathscr {M}_{\mathcal {H}_{1}}^{-} + i \mathscr {A}_{\mathcal {H}_{1}}^{-}, \mathscr {N}_{\mathcal {H}_{1}}^{-} + i \mathscr {B}_{\mathcal {H}_{1}}^{-} \right\rangle ,$$

and

$$\mathcal {H}_2 = \left\langle \mathscr {M}_{\mathcal {H}_{2}}^{+} + i \mathscr {A}_{\mathcal {H}_{2}}^{+}, \mathscr {N}_{\mathcal {H}_{2}}^{+} + i \mathscr {B}_{\mathcal {H}_{2}}^{+}, \mathscr {M}_{\mathcal {H}_{2}}^{-} + i \mathscr {A}_{\mathcal {H}_{2}}^{-}, \mathscr {N}_{\mathcal {H}_{2}}^{-} + i \mathscr {B}_{\mathcal {H}_{2}}^{-} \right\rangle .$$

Then, the following fundamental properties hold:

1. $$\mathcal {H}_{2} \oplus \mathcal {H}_{1}=\mathcal {H}_{1} \oplus \mathcal {H}_{2}.$$
2. $$\mathcal {H}_{2} \otimes \mathcal {H}_{1}=\mathcal {H}_1 \otimes \mathcal {H}_{2}$$.
3. $$\mathcal {H} \oplus (\mathcal {H}_{1} \oplus \mathcal {H}_{2})=(\mathcal {H} \oplus \mathcal {H}_{1}) \oplus \mathcal {H}_{2}$$.
4. $$\mathcal {H} \otimes (\mathcal {H}_{1} \otimes \mathcal {H}_{2})=(\mathcal {H} \otimes \mathcal {H}_{1}) \otimes \mathcal {H}_{2}$$.
5. $$\mathcal {H} \oplus (\mathcal {H}_{1} \cup \mathcal {H}_{2})=(\mathcal {H} \oplus \mathcal {H}_{1}) \cup (\mathcal {H} \oplus \mathcal {H}_{2})$$.
6. $$\mathcal {H} \oplus (\mathcal {H}_{1} \cap \mathcal {H}_{2})=(\mathcal {H} \oplus \mathcal {H}_{1}) \cap (\mathcal {H} \oplus \mathcal {H}_{2})$$.
7. $$\mathcal {H} \otimes (\mathcal {H}_{1} \cup \mathcal {H}_{2})=(\mathcal {H} \otimes \mathcal {H}_{1}) \cup (\mathcal {H} \otimes \mathcal {H}_{2})$$.
8. $$\mathcal {H} \otimes (\mathcal {H}_{1} \cap \mathcal {H}_{2})=(\mathcal {H} \otimes \mathcal {H}_{1}) \cap (\mathcal {H} \otimes \mathcal {H}_{2})$$.
9. $$\left( \mathcal {H}_1 \cup \mathcal {H}_2\right) \oplus \left( \mathcal {H}_1 \cap \mathcal {H}_2\right) =\mathcal {H}_1 \oplus \mathcal {H}_2$$.
10. $$\left( \mathcal {H}_1 \cup \mathcal {H}_2\right) \otimes \left( \mathcal {H}_1 \cap \mathcal {H}_2\right) =\mathcal {H}_1 \otimes \mathcal {H}_2$$.

### Proof

The parts (1), (3), and (5) will be demonstrated here, while the remaining parts can be similarly derived.

(1) The operation $\mathcal {H}_{1} \oplus \mathcal {H}_{2}$ is given by:

$$\mathcal {H}_{1} \oplus \mathcal {H}_{2} =$$

$$\left\langle \begin{array}{c} ((\mathscr {M}_{\mathcal {H}_1}^{+})^{q}+(\mathscr {M}_{\mathcal {H}_2}^{+})^{q}-(\mathscr {M}_{\mathcal {H}_1}^{+})^{q}(\mathscr {M}_{\mathcal {H}_2}^{+})^{q})^{\frac{1}{q}}\\ +i((\mathscr {A}_{\mathcal {H}_{1}}^{+})^{q}+(\mathscr {A}_{\mathcal {H}_{2}}^{+})^{q}-(\mathscr {A}_{\mathcal {H}_{1}}^{+})^{q} (\mathscr {A}_{\mathcal {H}_{2}}^{+})^{q})^{\frac{1}{q}},\\ (\mathscr {N}_{\mathcal {H}_1}^{+} \mathscr {N}_{\mathcal {H}_2}^{+})+i(\mathscr {B}_{\mathcal {H}_{1}}^{+}\mathscr {B}_{\mathcal {H}_{2}}^{+}),\\ -(\mathscr {M}_{\mathcal {H}_1}^{-} \mathscr {M}_{\mathcal {H}_2}^{-})+i(-(\mathscr {A}_{\mathcal {H}_{1}}^{-}\mathscr {A}_{\mathcal {H}_{2}}^{-})),\\ -(|\mathscr {N}_{\mathcal {H}_1}^{-}|^{q}+|\mathscr {N}_{\mathcal {H}_2}^{-}|^{q}-|\mathscr {N}_{\mathcal {H}_1}^{-}|^{q}|\mathscr {N}_{\mathcal {H}_2}^{-}|^{q})^{\frac{1}{q}}\\ +i(-(|\mathscr {B}_{\mathcal {H}_{1}}^{-}|^{q}+|\mathscr {B}_{\mathcal {H}_{2}}^{-}|^{q}-|\mathscr {B}_{\mathcal {H}_{1}}^{-}|^{q} |\mathscr {B}_{\mathcal {H}_{2}}^{-}|^{q})^{\frac{1}{q}}) \end{array} \right\rangle$$

which can also be rewritten as:

$$\left\langle \begin{array}{c} ((\mathscr {M}_{\mathcal {H}_2}^{+})^{q}+(\mathscr {M}_{\mathcal {H}_1}^{+})^{q}-(\mathscr {M}_{\mathcal {H}_2}^{+})^{q}(\mathscr {M}_{\mathcal {H}_1}^{+})^{q})^{\frac{1}{q}}\\ +i((\mathscr {A}_{\mathcal {H}_{2}}^{+})^{q}+(\mathscr {A}_{\mathcal {H}_{1}}^{+})^{q}-(\mathscr {A}_{\mathcal {H}_{2}}^{+})^{q} (\mathscr {A}_{\mathcal {H}_{1}}^{+})^{q})^{\frac{1}{q}},\\ (\mathscr {N}_{\mathcal {H}_2}^{+} \mathscr {N}_{\mathcal {H}_1}^{+})+i(\mathscr {B}_{\mathcal {H}_{2}}^{+}\mathscr {B}_{\mathcal {H}_{1}}^{+}),\\ -(\mathscr {M}_{\mathcal {H}_2}^{-} \mathscr {M}_{\mathcal {H}_1}^{-})+i(-(\mathscr {A}_{\mathcal {H}_{2}}^{-}\mathscr {A}_{\mathcal {H}_{1}}^{-})),\\ -(|\mathscr {N}_{\mathcal {H}_2}^{-}|^{q}+|\mathscr {N}_{\mathcal {H}_1}^{-}|^{q}-|\mathscr {N}_{\mathcal {H}_2}^{-}|^{q}|\mathscr {N}_{\mathcal {H}_1}^{-}|^{q})^{\frac{1}{q}}\\ +i(-(|\mathscr {B}_{\mathcal {H}_{2}}^{-}|^{q}+|\mathscr {B}_{\mathcal {H}_{1}}^{-}|^{q}-|\mathscr {B}_{\mathcal {H}_{2}}^{-}|^{q} |\mathscr {B}_{\mathcal {H}_{1}}^{-}|^{q})^{\frac{1}{q}}) \end{array} \right\rangle .$$

Thus, we conclude that:

$$\mathcal {H}_{1} \oplus \mathcal {H}_{2} = \mathcal {H}_{2} \oplus \mathcal {H}_{1}.$$

(3) $$\mathcal {H} \oplus (\mathcal {H}_{1} \oplus \mathcal {H}_{2})=$$

$$\begin{aligned} \langle \mathscr {M}_{\mathcal {H}}^{+}+i \mathscr {A}_{\mathcal {H}}^{+}, \mathscr {M}_{\mathcal {H}}^{-} +i \mathscr {A}_{\mathcal {H}}^{-}, \mathscr {N}_{\mathcal {H}}^{+} +i \mathscr {B}_{\mathcal {H}}^{+}, \mathscr {N}_{\mathcal {H}}^{-} +i \mathscr {B}_{\mathcal {H}}^{-}\rangle \end{aligned}$$

$$\begin{aligned} \oplus \end{aligned}$$

$$\left\langle \begin{array}{c} ((\mathscr {M}_{\mathcal {H}_1}^{+})^{q}+(\mathscr {M}_{\mathcal {H}_2}^{+})^{q}-(\mathscr {M}_{\mathcal {H}_1}^{+})^{q}(\mathscr {M}_{\mathcal {H}_2}^{+})^{q})^{\frac{1}{q}}\\ +i((\mathscr {A}_{\mathcal {H}_{1}}^{+})^{q}+(\mathscr {A}_{\mathcal {H}_{2}}^{+})^{q}-(\mathscr {A}_{\mathcal {H}_{1}}^{+})^{q} (\mathscr {A}_{\mathcal {H}_{2}}^{+})^{q})^{\frac{1}{q}},\\ (\mathscr {N}_{\mathcal {H}_1}^{+} \mathscr {N}_{\mathcal {H}_2}^{+})+i(\mathscr {B}_{\mathcal {H}_{1}}^{+}\mathscr {B}_{\mathcal {H}_{2}}^{+}),\\ -(\mathscr {M}_{\mathcal {H}_1}^{-} \mathscr {M}_{\mathcal {H}_2}^{-})+i(-(\mathscr {A}_{\mathcal {H}_{1}}^{-}\mathscr {A}_{\mathcal {H}_{2}}^{-})),\\ -(|\mathscr {N}_{\mathcal {H}_1}^{-}|^{q}+|\mathscr {N}_{\mathcal {H}_2}^{-}|^{q}-|\mathscr {N}_{\mathcal {H}_1}^{-}|^{q}|\mathscr {N}_{\mathcal {H}_2}^{-}|^{q})^{\frac{1}{q}}\\ +i(-(|\mathscr {B}_{\mathcal {H}_{1}}^{-}|^{q}+|\mathscr {B}_{\mathcal {H}_{2}}^{-}|^{q}-|\mathscr {B}_{\mathcal {H}_{1}}^{-}|^{q} |\mathscr {B}_{\mathcal {H}_{2}}^{-}|^{q})^{\frac{1}{q}}) \end{array}\right\rangle$$

$$=\left\langle \begin{array}{c} ((\mathscr {M}_{\mathcal {H}}^{+})^{q}+((\mathscr {M}_{\mathcal {H}_1}^{+})^{q}+(\mathscr {M}_{\mathcal {H}_2}^{+})^{q}\\ -(\mathscr {M}_{\mathcal {H}_1}^{+})^{q}(\mathscr {M}_{\mathcal {H}_2}^{+})^{q})-(\mathscr {M}_{\mathcal {H}}^{+})^{q}((\mathscr {M}_{\mathcal {H}_1}^{+})^{q}+(\mathscr {M}_{\mathcal {H}_2}^{+})^{q}\\ -(\mathscr {M}_{\mathcal {H}_1}^{+})^{q}(\mathscr {M}_{\mathcal {H}_2}^{+})^{q}))^{\frac{1}{q}}\\ +i((\mathscr {A}_{\mathcal {H}}^{+})^{q}+((\mathscr {A}_{\mathcal {H}_{1}}^{+})^{q}+(\mathscr {A}_{\mathcal {H}_{2}}^{+})^{q}\\ -(\mathscr {A}_{\mathcal {H}_{1}}^{+})^{q} (\mathscr {A}_{\mathcal {H}_{2}}^{+})^{q})\\ -(\mathscr {A}_{\mathcal {H}}^{+})^{q}((\mathscr {A}_{\mathcal {H}_{1}}^{+})^{q}+(\mathscr {A}_{\mathcal {H}_{2}}^{+})^{q}-(\mathscr {A}_{\mathcal {H}_{1}}^{+})^{q} (\mathscr {A}_{\mathcal {H}_{2}}^{+})^{q}))^{\frac{1}{q}},\\ (\mathscr {N}_{\mathcal {H}}^{+}\mathscr {N}_{\mathcal {H}_1}^{+} \mathscr {N}_{\mathcal {H}_2}^{+})+i(\mathscr {B}_{\mathcal {H}}^{+}\mathscr {B}_{\mathcal {H}_{1}}^{+}\mathscr {B}_{\mathcal {H}_{2}}^{+}),\\ -(|\mathscr {M}_{\mathcal {H}}^{-}||\mathscr {M}_{\mathcal {H}_1}^{-}| |\mathscr {M}_{\mathcal {H}_2}^{-}|)\\ +i(-(|\mathscr {A}_{\mathcal {H}}^{-}||\mathscr {A}_{\mathcal {H}_{1}}^{-}||\mathscr {A}_{\mathcal {H}_{2}}^{-}|)),\\ -(|\mathscr {N}_{\mathcal {H}}^{-}|^{q}+(|\mathscr {N}_{\mathcal {H}_1}^{-}|^{q}+|\mathscr {N}_{\mathcal {H}_2}^{-}|^{q}-|\mathscr {N}_{\mathcal {H}_1}^{-}|^{q}|\mathscr {N}_{\mathcal {H}_2}^{-}|^{q})\\ -|\mathscr {N}_{\mathcal {H}}^{-}|^{q}(|\mathscr {N}_{\mathcal {H}_1}^{-}|^{q}+|\mathscr {N}_{\mathcal {H}_2}^{-}|^{q}-|\mathscr {N}_{\mathcal {H}_1}^{-}|^{q}|\mathscr {N}_{\mathcal {H}_2}^{-}|^{q}))^{\frac{1}{q}}\\ +i(-(|\mathscr {B}_{\mathcal {H}}^{-}|^{q}+(|\mathscr {B}_{\mathcal {H}_{1}}^{-}|^{q}+|\mathscr {B}_{\mathcal {H}_{2}}^{-}|^{q}-|\mathscr {B}_{\mathcal {H}_{1}}^{-}|^{q} |\mathscr {B}_{\mathcal {H}_{2}}^{-}|^{q})\\ -|\mathscr {B}_{\mathcal {H}}^{-}|^{q}(|\mathscr {B}_{\mathcal {H}_{1}}^{-}|^{q}+|\mathscr {B}_{\mathcal {H}_{2}}^{-}|^{q}-|\mathscr {B}_{\mathcal {H}_{1}}^{-}|^{q} |\mathscr {B}_{\mathcal {H}_{2}}^{-}|^{q}))^{\frac{1}{q}})\end{array}\right\rangle$$

$$\begin{aligned}&=\left\langle \begin{array}{c} ((\mathscr {M}_{\mathcal {H}}^{+})^{q}+(\mathscr {M}_{\mathcal {H}_1}^{+})^{q}-(\mathscr {M}_{\mathcal {H}}^{+})^{q}(\mathscr {M}_{\mathcal {H}_1}^{+})^{q})^{\frac{1}{q}}\\ +i((\mathscr {A}_{\mathcal {H}}^{+})^{q}+(\mathscr {A}_{\mathcal {H}_{1}}^{+})^{q}-(\mathscr {A}_{\mathcal {H}}^{+})^{q} (\mathscr {A}_{\mathcal {H}_{1}}^{+})^{q})^{\frac{1}{q}},\\ (\mathscr {N}_{\mathcal {H}}^{+} \mathscr {N}_{\mathcal {H}_1}^{+})+i(\mathscr {B}_{\mathcal {H}}^{+}\mathscr {B}_{\mathcal {H}_{1}}^{+}) ,\\ -(\mathscr {M}_{\mathcal {H}}^{-} \mathscr {M}_{\mathcal {H}_1}^{-})+i(-(\mathscr {A}_{\mathcal {H}}^{-}\mathscr {A}_{\mathcal {H}_{1}}^{-})),\\ -(|\mathscr {N}_{\mathcal {H}}^{-}|^{q}+|\mathscr {N}_{\mathcal {H}_1}^{-}|^{q}-|\mathscr {N}_{\mathcal {H}}^{-}|^{q}|\mathscr {N}_{\mathcal {H}_1}^{-}|^{q})^{\frac{1}{q}}\\ +i(-(|\mathscr {B}_{\mathcal {H}}^{-}|^{q}+|\mathscr {B}_{\mathcal {H}_{1}}^{-}|^{q}-|\mathscr {B}_{\mathcal {H}}^{-}|^{q} |\mathscr {B}_{\mathcal {H}_{1}}^{-}|^{q})^{\frac{1}{q}})\end{array}\right\rangle\\ &\,\,\,\,\,\,\,\,\,\,\,\,\,\,\,\,\,\,\,\,\,\,\,\,\,\,\,\,\,\,\,\,\,\,\,\,\,\,\,\,\,\,\,\,\,\,\,\,\,\,\,\,\, \oplus\\ & \langle \mathscr {M}_{\mathcal {H}_{2}}^{+}+i \mathscr {A}_{\mathcal {H}_{2}}^{+}, \mathscr {M}_{\mathcal {H}_{2}}^{-} +i \mathscr {A}_{\mathcal {H}_{2}}^{-}, \mathscr {N}_{\mathcal {H}_{2}}^{+} +i \mathscr {B}_{\mathcal {H}_{2}}^{+}, \mathscr {N}_{\mathcal {H}_{2}}^{-} +i \mathscr {B}_{\mathcal {H}_{2}}^{-}\rangle \\ & =(\mathcal {H} \oplus \mathcal {H}_{1}) \oplus \mathcal {H}_{2}.\end{aligned}$$

(5)

$$\begin{aligned}&\mathcal {H} \oplus (\mathcal {H}_{1} \cup \mathcal {H}_{2})=\langle \mathscr {M}_{\mathcal {H}}^{+}+i \mathscr {A}_{\mathcal {H}}^{+}, \mathscr {M}_{\mathcal {H}}^{-} +i \mathscr {A}_{\mathcal {H}}^{-}, \mathscr {N}_{\mathcal {H}}^{+} +i \mathscr {B}_{\mathcal {H}}^{+}, \mathscr {N}_{\mathcal {H}}^{-} +i \mathscr {B}_{\mathcal {H}}^{-}\rangle\\ &\,\,\,\,\,\,\,\,\,\,\,\,\,\,\,\,\,\,\,\,\,\,\,\,\,\,\,\,\,\,\,\,\,\,\,\,\,\,\,\,\,\,\,\,\,\,\,\,\,\,\,\,\,\,\,\,\,\,\,\,\,\,\,\,\,\,\,\,\,\,\,\,\,\,\,\,\,\,\,\,\,\,\, \oplus\\ & \left\langle \begin{array}{c} \max \{\mathscr {M}_{\mathcal {H}_1}^{+}, \mathscr {M}_{\mathcal {H}_2}^{+}\} +i \max \{\mathscr {A}_{\mathcal {H}_1}^{+}, \mathscr {A} _{\mathcal {H}_2}^{+}\},\min \{\mathscr {N}_{\mathcal {H}_1}^{+}, \mathscr {N}_{\mathcal {H}_2}^{+}\} +i \min \{\mathscr {B}_{\mathcal {H}_1}^{+}, \mathscr {B}_ {\mathcal {H}_2}^{+}\},\\ \min \{\mathscr {M}_{\mathcal {H}_1}^{-}, \mathscr {M}_{\mathcal {H}_2}^{-}\}+i \min \{\mathscr {A}_{\mathcal {H}_1}^{-}, \mathscr {A}_{\mathcal {H}_2}^{-}\}, \max \{\mathscr {N}_{\mathcal {H}_1}^{-}, \mathscr {N}_{\mathcal {H}_2}^{-}\} +i \max \{\mathscr {B}_ {\mathcal {H}_1}^{-}, \mathscr {B}_ {\mathcal {H}_2}^{-}\}\end{array}\right\rangle\end{aligned}$$

$$\begin{aligned}& =\left\langle \begin{array}{c} ((\mathscr {M}_{\mathcal {H}}^{+})^{q}+\max \{\mathscr {M}_{\mathcal {H}_1}^{+}, \mathscr {M}_{\mathcal {H}_2}^{+}\}^{q}-(\mathscr {M}_{\mathcal {H}}^{+})^{q}\max \{\mathscr {M}_{\mathcal {H}_1}^{+}, \mathscr {M}_{\mathcal {H}_2}^{+}\}^{q})^\frac{1}{q}\\ +i ((\mathscr {A}_{\mathcal {H}}^{+})^{q}+\max \{\mathscr {A}_{\mathcal {H}_1}^{+}, \mathscr {A} _{\mathcal {H}_2}^{+}\}^{q}-(\mathscr {A}_{\mathcal {H}}^{+})^{q}\max \{\mathscr {A}_{\mathcal {H}_1}^{+}, \mathscr {A} _{\mathcal {H}_2}^{+}\}^{q})^\frac{1}{q},\\ (\mathscr {N}_{\mathcal {H}}^{+} \min \{\mathscr {N}_{\mathcal {H}_1}^{+}, \mathscr {N}_{\mathcal {H}_2}^{+}\}) +i (\mathscr {B}_{\mathcal {H}} \min \{\mathscr {B}_{\mathcal {H}_1}^{+}, \mathscr {B}_ {\mathcal {H}_2}^{+}\}),\\ -(\mathscr {M}_{\mathcal {H}}^{-}\min \{\mathscr {M}_{\mathcal {H}_1}^{-}, \mathscr {M}_{\mathcal {H}_2}^{-}\}) +i (-(\mathscr {A}_{\mathcal {H}}^{-}\min \{\mathscr {A}_{\mathcal {H}_1}^{-}, \mathscr {A}_{\mathcal {H}_2}^{-}\})),\\ - (|\mathscr {N}_{\mathcal {H}}^{-}|^{q}+|\max \{\mathscr {N}_{\mathcal {H}_1}^{-}, \mathscr {N}_{\mathcal {H}_2}^{-}\}|^{q}-|\mathscr {N}_{\mathcal {H}}^{-}|^{q}|\max \{\mathscr {N}_{\mathcal {H}_1}^{-}, \mathscr {N}_{\mathcal {H}_2}^{-}\}|^{q})^\frac{1}{q}\\ +i(-(|\mathscr {B}_{\mathcal {H}}^{-}|^{q}+|\max \{\mathscr {B}_ {\mathcal {H}_1}^{-}, \mathscr {B}_ {\mathcal {H}_2}^{-}\}|^{q}-|\mathscr {B}_{\mathcal {H}}^{-}|^{q}|\max \{\mathscr {B}_ {\mathcal {H}_1}^{-}, \mathscr {B}_ {\mathcal {H}_2}^{-}\}|^{q})^\frac{1}{q})\end{array}\right\rangle\\ & =\left\langle \begin{array}{c} ((\mathscr {M}_{\mathcal {H}}^{+})^{q}+(1-(\mathscr {M}_{\mathcal {H}}^{+})^{q})\max \{(\mathscr {M}_{\mathcal {H}_1}^{+})^{q},(\mathscr {M}_{\mathcal {H}_2}^{+})^{q}\})^{\frac{1}{q}}+ \\ i((\mathscr {A}_{\mathcal {H}}^{+})^{q}+(1-(\mathscr {A}_{\mathcal {H}}^{+})^{q})\max \{(\mathscr {A}_{\mathcal {H}_{1}}^{+})^{q},(\mathscr {A}_{\mathcal {H}_{2}}^{+})^{q}\})^{\frac{1}{q}},\\ \min \{\mathscr {N}_{\mathcal {H}}^{+}\mathscr {N}_{\mathcal {H}_{1}}^{+},\mathscr {N}_{\mathcal {H}}^{+}\mathscr {N}_{\mathcal {H}_{2}}^{+}\}+i(\min \{\mathscr {B}_{\mathcal {H}}^{+}\mathscr {B}_{\mathcal {H}_{1}}^{+},\mathscr {B}_{\mathcal {H}}^{+}\mathscr {B}_{\mathcal {H}_{2}}^{+}\}),\\ \min \{-(\mathscr {M}_{\mathcal {H}}^{-}\mathscr {M}_{\mathcal {H}_{1}}^{-}),-(\mathscr {M}_{\mathcal {H}}^{-}\mathscr {M}_{\mathcal {H}_{2}}^{-})\}+\\ i(\min \{-(\mathscr {A}_{\mathcal {H}}^{-}\mathscr {A}_{\mathcal {H}_{1}}^{-}),-(\mathscr {A}_{\mathcal {H}}^{-}\mathscr {A}_{\mathcal {H}_{2}}^{-})\}),\\ -(|\mathscr {N}_{\mathcal {H}}^{-}|^{q}+(1-|\mathscr {N}_{\mathcal {H}}^{-}|^{q})\max \{|\mathscr {N}_{\mathcal {H}_1}^{-}|^{q},|\mathscr {N}_{\mathcal {H}_2}^{-}|^{q}\})^{\frac{1}{q}}\\ +i(-(|\mathscr {B}_{\mathcal {H}}^{-}|^{q}+(1-|\mathscr {B}_{\mathcal {H}}^{-}|^{q})\max \{|\mathscr {B}_{\mathcal {H}_{1}}^{-}|^{q},|\mathscr {B}_{\mathcal {H}_{2}}^{-}|^{q}\})^{\frac{1}{q}})\end{array}\right\rangle .\end{aligned}$$

However,

$$\begin{aligned}& (\mathcal {H} \oplus \mathcal {H}_{1}) \cup (\mathcal {H} \oplus \mathcal {H}_{2})=\\ & \left\langle \begin{array}{c} ((\mathscr {M}_{\mathcal {H}}^{+})^{q}+(\mathscr {M}_{\mathcal {H}_1}^{+})^{q}-(\mathscr {M}_{\mathcal {H}}^{+})^{q}(\mathscr {M}_{\mathcal {H}_1}^{+})^{q})^{\frac{1}{q}}\\ +i((\mathscr {A}_{\mathcal {H}}^{+})^{q}+(\mathscr {A}_{\mathcal {H}_{1}}^{+})^{q}-(\mathscr {A}_{\mathcal {H}}^{+})^{q} (\mathscr {A}_{\mathcal {H}_{1}}^{+})^{q})^{\frac{1}{q}},\\ (\mathscr {N}_{\mathcal {H}}^{+} \mathscr {N}_{\mathcal {H}_1}^{+})+i(\mathscr {B}_{\mathcal {H}}^{+}\mathscr {B}_{\mathcal {H}_{1}}^{+}) , \\ -(\mathscr {M}_{\mathcal {H}}^{-} \mathscr {M}_{\mathcal {H}_1}^{-})+i(-(\mathscr {A}_{\mathcal {H}}^{-}\mathscr {A}_{\mathcal {H}_{1}}^{-})),\\ -(|\mathscr {N}_{\mathcal {H}}^{-}|^{q}+|\mathscr {N}_{\mathcal {H}_1}^{-}|^{q}-|\mathscr {N}_{\mathcal {H}}^{-}|^{q}|\mathscr {N}_{\mathcal {H}_1}^{-}|^{q})^{\frac{1}{q}}\\ +i(-(|\mathscr {B}_{\mathcal {H}}^{-}|^{q}+|\mathscr {B}_{\mathcal {H}_{1}}^{-}|^{q}-|\mathscr {B}_{\mathcal {H}}^{-}|^{q} |\mathscr {B}_{\mathcal {H}_{1}}^{-}|^{q})^{\frac{1}{q}}) \end{array}\right\rangle\\ &\,\,\,\,\,\,\,\,\,\,\,\,\,\,\,\,\,\,\,\,\,\,\,\,\,\,\,\,\,\,\,\,\,\,\,\,\,\,\,\,\,\,\,\,\,\,\,\,\,\, \cup \end{aligned}$$

$$\left\langle \begin{array}{c} ((\mathscr {M}_{\mathcal {H}}^{+})^{q}+(\mathscr {M}_{\mathcal {H}_2}^{+})^{q}-(\mathscr {M}_{\mathcal {H}}^{+})^{q}(\mathscr {M}_{\mathcal {H}_2}^{+})^{q})^{\frac{1}{q}}\\ +i((\mathscr {A}_{\mathcal {H}}^{+})^{q}+(\mathscr {A}_{\mathcal {H}}^{+})^{q}-(\mathscr {A}_{\mathcal {H}}^{+})^{q} (\mathscr {A}_{\mathcal {H}_{2}}^{+})^{q})^{\frac{1}{q}},\\ (\mathscr {N}_{\mathcal {H}}^{+} \mathscr {N}_{\mathcal {H}_2}^{+})+i(\mathscr {B}_{\mathcal {H}}^{+}\mathscr {B}_{\mathcal {H}_{2}}^{+}) , \\ -(\mathscr {M}_{\mathcal {H}}^{-} \mathscr {M}_{\mathcal {H}_2}^{-})+i(-(\mathscr {A}_{\mathcal {H}}^{-}\mathscr {A}_{\mathcal {H}_{2}}^{-})),\\ -(|\mathscr {N}_{\mathcal {H}}^{-}|^{q}+|\mathscr {N}_{\mathcal {H}_2}^{-}|^{q}-|\mathscr {N}_{\mathcal {H}}^{-}|^{q}|\mathscr {N}_{\mathcal {H}_2}^{-}|^{q})^{\frac{1}{q}}\\ +i(-(|\mathscr {B}_{\mathcal {H}}^{-}|^{q}+|\mathscr {B}_{\mathcal {H}_{2}}^{-}|^{q}-|\mathscr {B}_{\mathcal {H}}^{-}|^{q} |\mathscr {B}_{\mathcal {H}_{2}}^{-}|^{q})^{\frac{1}{q}})\end{array}\right\rangle$$

$$\begin{aligned}&=\left\langle \begin{array}{c} \max \{((\mathscr {M}_{\mathcal {H}}^{+})^{q}+(\mathscr {M}_{\mathcal {H}_1}^{+})^{q}-(\mathscr {M}_{\mathcal {H}}^{+})^{q}(\mathscr {M}_{\mathcal {H}_1}^{+})^{q})^{\frac{1}{q}},\\ ((\mathscr {M}_{\mathcal {H}}^{+})^{q}+(\mathscr {M}_{\mathcal {H}_2}^{+})^{q}-(\mathscr {M}_{\mathcal {H}}^{+})^{q}(\mathscr {M}_{\mathcal {H}_2}^{+})^{q})^{\frac{1}{q}}\}\\ +i(\max \{((\mathscr {A}_{\mathcal {H}}^{+})^{q}+(\mathscr {A}_{\mathcal {H}_{1}}^{+})^{q}-(\mathscr {A}_{\mathcal {H}}^{+})^{q} (\mathscr {A}_{\mathcal {H}_{1}}^{+})^{q})^{\frac{1}{q}},\\ ((\mathscr {A}_{\mathcal {H}}^{+})^{q}+(\mathscr {A}_{\mathcal {H}}^{+})^{q}-(\mathscr {A}_{\mathcal {H}}^{+})^{q} (\mathscr {A}_{\mathcal {H}_{2}}^{+})^{q})^{\frac{1}{q}}\}),\\ \min \{\mathscr {N}_{\mathcal {H}}^{+}\mathscr {N}_{\mathcal {H}_{1}}^{+},\mathscr {N}_{\mathcal {H}}^{+}\mathscr {N}_{\mathcal {H}_{2}}^{+}\}+\\ i(\min \{\mathscr {B}_{\mathcal {H}}^{+}\mathscr {B}_{\mathcal {H}_{1}}^{+},\mathscr {B}_{\mathcal {H}}^{+}\mathscr {B}_{\mathcal {H}_{2}}^{+}\}),\\ \min \{-(\mathscr {M}_{\mathcal {H}}^{-}\mathscr {M}_{\mathcal {H}_{1}}^{-}),-(\mathscr {M}_{\mathcal {H}}^{-}\mathscr {M}_{\mathcal {H}_{2}}^{-})\}+\\ i(\min \{-(\mathscr {A}_{\mathcal {H}}^{-}\mathscr {A}_{\mathcal {H}_{1}}^{-}),\\ -(\mathscr {A}_{\mathcal {H}}^{-}\mathscr {A}_{\mathcal {H}_{2}}^{-})\}),\\ \max \{-(|\mathscr {N}_{\mathcal {H}}^{-}|^{q}+|\mathscr {N}_{\mathcal {H}_1}^{-}|^{q}-|\mathscr {N}_{\mathcal {H}}^{-}|^{q}|\mathscr {N}_{\mathcal {H}_1}^{-}|^{q})^{\frac{1}{q}},\\ -(|\mathscr {N}_{\mathcal {H}}^{-}|^{q}+|\mathscr {N}_{\mathcal {H}_2}^{-}|^{q}-|\mathscr {N}_{\mathcal {H}}^{-}|^{q}|\mathscr {N}_{\mathcal {H}_2}^{-}|^{q})^{\frac{1}{q}}\}\\ +i(\max \{-(|\mathscr {B}_{\mathcal {H}}^{-}|^{q}+|\mathscr {B}_{\mathcal {H}_{1}}^{-}|^{q}-|\mathscr {B}_{\mathcal {H}}^{-}|^{q} |\mathscr {B}_{\mathcal {H}_{1}}^{-}|^{q})^{\frac{1}{q}},\\ -(|\mathscr {B}_{\mathcal {H}}^{-}|^{q}+|\mathscr {B}_{\mathcal {H}_{2}}^{-}|^{q}-|\mathscr {B}_{\mathcal {H}}^{-}|^{q} |\mathscr {B}_{\mathcal {H}_{2}}^{-}|^{q})^{\frac{1}{q}}\})\end{array}\right\rangle \end{aligned}$$

$$\begin{aligned}& =\left\langle \begin{array}{c} ((\mathscr {M}_{\mathcal {H}}^{+})^{q}+(1-(\mathscr {M}_{\mathcal {H}}^{+})^{q})\max \{(\mathscr {M}_{\mathcal {H}_1}^{+})^{q},\\ (\mathscr {M}_{\mathcal {H}_2}^{+})^{q}\})^{\frac{1}{q}}+i((\mathscr {A}_{\mathcal {H}}^{+})^{q}+(1-(\mathscr {A}_{\mathcal {H}}^{+})^{q})\max \{(\mathscr {A}_{\mathcal {H}_{1}}^{+})^{q},(\mathscr {A}_{\mathcal {H}_{2}}^{+})^{q}\})^{\frac{1}{q}},\\ \min \{\mathscr {N}_{\mathcal {H}}^{+}\mathscr {N}_{\mathcal {H}_{1}}^{+},\mathscr {N}_{\mathcal {H}}^{+}\mathscr {N}_{\mathcal {H}_{2}}^{+}\}\\ +i(\min \{\mathscr {B}_{\mathcal {H}}^{+}\mathscr {B}_{\mathcal {H}_{1}}^{+},\mathscr {B}_{\mathcal {H}}^{+}\mathscr {B}_{\mathcal {H}_{2}}^{+}\}),\\ \min \{-(\mathscr {M}_{\mathcal {H}}^{-}\mathscr {M}_{\mathcal {H}_{1}}^{-}),-(\mathscr {M}_{\mathcal {H}}^{-}\mathscr {M}_{\mathcal {H}_{2}}^{-})\}+\\ i(\min \{-(\mathscr {A}_{\mathcal {H}}^{-}\mathscr {A}_{\mathcal {H}_{1}}^{-}),-(\mathscr {A}_{\mathcal {H}}^{-}\mathscr {A}_{\mathcal {H}_{2}}^{-})\}),\\ -(|\mathscr {N}_{\mathcal {H}}^{-}|^{q}+(1-|\mathscr {N}_{\mathcal {H}}^{-}|^{q})\max \{|\mathscr {N}_{\mathcal {H}_1}^{-}|^{q},|\mathscr {N}_{\mathcal {H}_2}^{-}|^{q}\})^{\frac{1}{q}}\\ +i(-(|\mathscr {B}_{\mathcal {H}}^{-}|^{q}+(1-|\mathscr {B}_{\mathcal {H}}^{-}|^{q})\max \{|\mathscr {B}_{\mathcal {H}_{1}}^{-}|^{q},|\mathscr {B}_{\mathcal {H}_{2}}^{-}|^{q}\})^{\frac{1}{q}})\end{array}\right\rangle . \end{aligned}$$

$$\square$$

### Theorem 3.13

Let

$$\mathcal {H} = \left\langle \mathscr {M}_{\mathcal {H}}^{+} + i \mathscr {A}_{\mathcal {H}}^{+}, \mathscr {N}_{\mathcal {H}}^{+} + i \mathscr {B}_{\mathcal {H}}^{+}, \mathscr {M}_{\mathcal {H}}^{-} + i \mathscr {A}_{\mathcal {H}}^{-}, \mathscr {N}_{\mathcal {H}}^{-} + i \mathscr {B}_{\mathcal {H}}^{-} \right\rangle ,$$

$$\mathcal {H}_1 = \left\langle \mathscr {M}_{\mathcal {H}_{1}}^{+} + i \mathscr {A}_{\mathcal {H}_{1}}^{+}, \mathscr {N}_{\mathcal {H}_{1}}^{+} + i \mathscr {B}_{\mathcal {H}_{1}}^{+}, \mathscr {M}_{\mathcal {H}_{1}}^{-} + i \mathscr {A}_{\mathcal {H}_{1}}^{-}, \mathscr {N}_{\mathcal {H}_{1}}^{-} + i \mathscr {B}_{\mathcal {H}_{1}}^{-} \right\rangle ,$$

and

$$\mathcal {H}_2 = \left\langle \mathscr {M}_{\mathcal {H}_{2}}^{+} + i \mathscr {A}_{\mathcal {H}_{2}}^{+}, \mathscr {N}_{\mathcal {H}_{2}}^{+} + i \mathscr {B}_{\mathcal {H}_{2}}^{+}, \mathscr {M}_{\mathcal {H}_{2}}^{-} + i \mathscr {A}_{\mathcal {H}_{2}}^{-}, \mathscr {N}_{\mathcal {H}_{2}}^{-} + i \mathscr {B}_{\mathcal {H}_{2}}^{-} \right\rangle$$

be BCq-ROFNs. For any $\mathscr {I}>0$, the following characteristics are satisfied:

1. $$(\mathcal {H}_{1} \oplus \mathcal {H}_{2})^c=\mathcal {H}_{1}^c \otimes \mathcal {H}_{2}^c$$.
2. $$(\mathcal {H}_{1} \otimes \mathcal {H}_{2})^{c}=\mathcal {H}_{1}^c \oplus \mathcal {H}_{2}^{c}$$.
3. $$(\mathcal {H}^{c})^{\mathscr {I}}=(\mathscr {I}\mathcal {H})^{c}$$.
4. $$\mathscr {I}(\mathcal {H})^{c}=(\mathcal {H}^{\mathscr {I}})^{c}$$.

### Proof

Parts (1) and (3) will be displayed here. Similarly, the other sections can be presented as well.

(1)

$$(\mathcal {H}_{1} \oplus \mathcal {H}_{2})^{c}={\left\langle \begin{array}{c} ((\mathscr {M}_{\mathcal {H}_1}^{+})^{q}+(\mathscr {M}_{\mathcal {H}_2}^{+})^{q}-(\mathscr {M}_{\mathcal {H}_1}^{+})^{q}(\mathscr {M}_{\mathcal {H}_2}^{+})^{q})^{\frac{1}{q}}\\ +i((\mathscr {A}_{\mathcal {H}_{1}}^{+})^{q}+(\mathscr {A}_{\mathcal {H}_{2}}^{+})^{q}-(\mathscr {A}_{\mathcal {H}_{1}}^{+})^{q} (\mathscr {A}_{\mathcal {H}_{2}}^{+})^{q})^{\frac{1}{q}},\\ (\mathscr {N}_{\mathcal {H}_1}^{+} \mathscr {N}_{\mathcal {H}_2}^{+})+i(\mathscr {B}_{\mathcal {H}_{1}}^{+}\mathscr {B}_{\mathcal {H}_{2}}^{+}),\\ -(\mathscr {M}_{\mathcal {H}_1}^{-} \mathscr {M}_{\mathcal {H}_2}^{-})+i(-(\mathscr {A}_{\mathcal {H}_{1}}^{-}\mathscr {A}_{\mathcal {H}_{2}}^{-})),\\ -(|\mathscr {N}_{\mathcal {H}_1}^{-}|^{q}+|\mathscr {N}_{\mathcal {H}_2}^{-}|^{q}-|\mathscr {N}_{\mathcal {H}_1}^{-}|^{q}|\mathscr {N}_{\mathcal {H}_2}^{-}|^{q})^{\frac{1}{q}}\\ +i(-(|\mathscr {B}_{\mathcal {H}_{1}}^{-}|^{q}+|\mathscr {B}_{\mathcal {H}_{2}}^{-}|^{q}-|\mathscr {B}_{\mathcal {H}_{1}}^{-}|^{q} |\mathscr {B}_{\mathcal {H}_{2}}^{-}|^{q})^{\frac{1}{q}})\end{array}\right\rangle }^{c}$$

$$\begin{aligned}& = \left\langle \begin{array}{c} (\mathscr {N}_{\mathcal {H}_1}^{+} \mathscr {N}_{\mathcal {H}_2}^{+})+i(\mathscr {B}_{\mathcal {H}_{1}}^{+}\mathscr {B}_{\mathcal {H}_{2}}^{+}),\\ ((\mathscr {M}_{\mathcal {H}_1}^{+})^{q}+(\mathscr {M}_{\mathcal {H}_2}^{+})^{q}-(\mathscr {M}_{\mathcal {H}_1}^{+})^{q}(\mathscr {M}_{\mathcal {H}_2}^{+})^{q})^{\frac{1}{q}}\\ +i((\mathscr {A}_{\mathcal {H}_{1}}^{+})^{q}+(\mathscr {A}_{\mathcal {H}_{2}}^{+})^{q}-(\mathscr {A}_{\mathcal {H}_{1}}^{+})^{q} (\mathscr {A}_{\mathcal {H}_{2}}^{+})^{q})^{\frac{1}{q}}, \\ -|-(|\mathscr {N}_{\mathcal {H}_1}^{-}|^{q}+|\mathscr {N}_{\mathcal {H}_2}^{-}|^{q}-|\mathscr {N}_{\mathcal {H}_1}^{-}|^{q}|\mathscr {N}_{\mathcal {H}_2}^{-}|^{q})^{\frac{1}{q}}|\\ +i(-|-(|\mathscr {B}_{\mathcal {H}_{1}}^{-}|^{q}+|\mathscr {B}_{\mathcal {H}_{2}}^{-}|^{q}-|\mathscr {B}_{\mathcal {H}_{1}}^{-}|^{q} |\mathscr {B}_{\mathcal {H}_{2}}^{-}|^{q})^{\frac{1}{q}}|) \\ -|-(\mathscr {M}_{\mathcal {H}_1}^{-} \mathscr {M}_{\mathcal {H}_2}^{-})|+i(-|-(\mathscr {A}_{\mathcal {H}_{1}}^{-}\mathscr {A}_{\mathcal {H}_{2}}^{-})|)\end{array}\right\rangle\\ & =\langle ( \mathscr {N}_{\mathcal {H}_1}^{+}) +i (\mathscr {B}_{\mathcal {H}_1}^{+}), (\mathscr {M}_{\mathcal {H}_1}^{+}) +i (\mathscr {A}_{\mathcal {H}_1}^{+}), -|\mathscr {N}_{\mathcal {H}_1}^{-}| +i (-|\mathscr {B}_{\mathcal {H}_1}^{-}|), -|\mathscr {M}_{\mathcal {H}_1}^{-}| +i (-|\mathscr {A}_{\mathcal {H}_1}^{-}|)\rangle\\ &\,\,\,\,\,\,\,\,\,\,\,\,\,\,\,\,\,\,\,\,\,\,\,\,\,\,\,\,\,\,\,\,\,\,\,\,\,\,\,\,\,\,\,\,\,\,\,\,\,\,\,\,\,\,\,\,\,\,\,\,\,\,\,\,\,\,\,\, \otimes\\ & \langle ( \mathscr {N}_{\mathcal {H}_2}^{+}) +i (\mathscr {B}_{\mathcal {H}_2}^{+}), (\mathscr {M}_{\mathcal {H}_2}^{+}) +i (\mathscr {A}_{\mathcal {H}_2}^{+}), -|\mathscr {N}_{\mathcal {H}_2}^{-}| +i (-|\mathscr {B}_{\mathcal {H}_2}^{-}|), -|\mathscr {M}_{\mathcal {H}_2}^{-}| +i (-|\mathscr {A}_{\mathcal {H}_2}^{-}|)\rangle\\ & =\mathcal {H}_{1}^c \otimes \mathcal {H}_{2}^c.\end{aligned}$$

(3)

$$\begin{aligned} & (\mathcal {H}^{c})^{\mathscr {I}}\\ & =\left\langle (\mathscr {N}_{\mathcal {H}}^{+}) +i (\mathscr {B}_{\mathcal {H}}^{+}), (\mathscr {M}_{\mathcal {H}}^{+}) +i (\mathscr {A}_{\mathcal {H}}^{+}), -|\mathscr {N}_{\mathcal {H}}^{-}| +i (-|\mathscr {B}_{\mathcal {H}}^{-}|) , -|\mathscr {M}_{\mathcal {H}}^{-}| +i (-|\mathscr {A}_{\mathcal {H}}^{-}|)\right\rangle ^{\mathscr {I}} \\ & =\left\langle \begin{array}{c} ( \mathscr {N}_{\mathcal {H}}^{+})^{\mathscr {I}}+i(\mathscr {B}_{\mathcal {H}}^{+})^{\mathscr {I}},(1-(1-(\mathscr {M}_{\mathcal {H}}^{+})^{q})^\mathscr {I})^{\frac{1}{q}}\\ +i(1-(1-(\mathscr {A}_{\mathcal {H}}^{+})^{q})^{\mathscr {I}})^{\frac{1}{q}},\\ -(1-(1-|-|\mathscr {N}_{\mathcal {H}}^{-}||^{q})^{\mathscr {I}})^\frac{1}{q}\\ +i(-(1-(1-|-|\mathscr {B}_{\mathcal {H}}^{-}||^{q})^{\mathscr {I}})^{\frac{1}{q}}),\\ -|-|\mathscr {M}_{\mathcal {H}}^{-}||^{\mathscr {I}}+i(-|-|\mathscr {A}_{\mathcal {H}}^{-}||^{\mathscr {I}})\end{array}\right\rangle \end{aligned}$$

$$\begin{aligned} & =\left\langle \begin{array}{c} (( \mathscr {N}_{\mathcal {H}}^{+})^{\mathscr {I}})+i((\mathscr {B}_{\mathcal {H}}^{+})^{\mathscr {I}}),((1-(1-(\mathscr {M}_{\mathcal {H}}^{+})^{q})^\mathscr {I})^\frac{1}{q})\\ +i((1-(1-(\mathscr {A}_{\mathcal {H}}^{+})^{q})^{\mathscr {I}})^{\frac{1}{q}}),\\ -|-(1-(1-|-|\mathscr {N}_{\mathcal {H}}^{-}|^{q}|^{\mathscr {I}})^{\frac{1}{q}}|\\ +i(-|-(1-(1-|\mathscr {B}_{\mathcal {H}}^{-}|^{q})^{\mathscr {I}})^{\frac{1}{q}}|),\\ -|-|\mathscr {M}_{\mathcal {H}}^{-}|^{\mathscr {I}}|+i(-|-|\mathscr {A}_{\mathcal {H}}^{-}|^{\mathscr {I}}|)\end{array}\right\rangle\\ & =\left\langle \begin{array}{c} (1-(1-(\mathscr {M}_{\mathcal {H}}^{+})^{q})^\mathscr {I})^\frac{1}{q}\\ +i(1-(1-(\mathscr {A}_{\mathcal {H}}^{+})^{q})^{\mathscr {I}})^{\frac{1}{q}},\\ (\mathscr {N}_{\mathcal {H}}^{+})^{\mathscr {I}}+i(\mathscr {B}_{\mathcal {H}}^{+})^{\mathscr {I}},\\ -|\mathscr {M}_{\mathcal {H}}^{-}|^{\mathscr {I}}+i(-|\mathscr {A}_{\mathcal {H}}^{-}|^{\mathscr {I}}),\\ -(1-(1-|\mathscr {N}_{\mathcal {H}}^{-}|^{q}|)^{\mathscr {I}})^{\frac{1}{q}}\\ +i(-(1-(1-|\mathscr {B}_{\mathcal {H}}^{-}|^{q}|)^{\mathscr {I}})^{\frac{1}{q}})\end{array}\right\rangle ^{c}\\ & =(\mathscr {I}\mathcal {H})^{c}. \end{aligned}$$

$$\square$$

### Theorem 3.14

Let

$$\mathcal {H} = \left\langle \mathscr {M}_{\mathcal {H}}^{+} + i \mathscr {A}_{\mathcal {H}}^{+}, \mathscr {N}_{\mathcal {H}}^{+} + i \mathscr {B}_{\mathcal {H}}^{+}, \mathscr {M}_{\mathcal {H}}^{-} + i \mathscr {A}_{\mathcal {H}}^{-}, \mathscr {N}_{\mathcal {H}}^{-} + i \mathscr {B}_{\mathcal {H}}^{-} \right\rangle ,$$

$$\mathcal {H}_1 = \left\langle \mathscr {M}_{\mathcal {H}_{1}}^{+} + i \mathscr {A}_{\mathcal {H}_{1}}^{+}, \mathscr {N}_{\mathcal {H}_{1}}^{+} + i \mathscr {B}_{\mathcal {H}_{1}}^{+}, \mathscr {M}_{\mathcal {H}_{1}}^{-} + i \mathscr {A}_{\mathcal {H}_{1}}^{-}, \mathscr {N}_{\mathcal {H}_{1}}^{-} + i \mathscr {B}_{\mathcal {H}_{1}}^{-} \right\rangle ,$$

and

$$\mathcal {H}_2 = \left\langle \mathscr {M}_{\mathcal {H}_{2}}^{+} + i \mathscr {A}_{\mathcal {H}_{2}}^{+}, \mathscr {N}_{\mathcal {H}_{2}}^{+} + i \mathscr {B}_{\mathcal {H}_{2}}^{+}, \mathscr {M}_{\mathcal {H}_{2}}^{-} + i \mathscr {A}_{\mathcal {H}_{2}}^{-}, \mathscr {N}_{\mathcal {H}_{2}}^{-} + i \mathscr {B}_{\mathcal {H}_{2}}^{-} \right\rangle$$

be BCq-ROFNs. For any $\mathscr {I}, \mathscr {I}_1, \mathscr {I}_2>0$, the following characteristics are satisfied:

1. $$\mathscr {I}\left( \mathcal {H}_1\oplus \mathcal {H}_2\right) =\mathscr {I} \mathcal {H}_1 \oplus \mathscr {I} \mathcal {H}_2$$.
2. $$\left( \mathscr {I}_1+\mathscr {I}_2\right) \mathcal {H}=\mathscr {I}_1 \mathcal {H} \oplus \mathscr {I}_2 \mathcal {H}$$.
3. $$\left( \mathcal {H}_1 \otimes \mathcal {H}_2\right) ^\mathscr {I}=\mathcal {H}_1^\mathscr {I} \otimes \mathcal {H}_2^\mathscr {I}$$.
4. $$\mathcal {H}^{\left( \mathscr {I}_1+\mathscr {I}_2\right) }=\mathcal {H}^{\mathscr {I}_1} \otimes \mathcal {H}^{\mathscr {I}_2}$$.

### Proof

Only expressions (1) and (2) are explicitly illustrated at this stage; however, the subsequent components may be derived or expressed analogously. 

(1)

$$\mathscr {I}\left( \mathcal {H}_1\oplus \mathcal {H}_2\right) =$$

$$\begin{aligned}&\mathscr {I}\left\langle \begin{array}{c} ((\mathscr {M}_{\mathcal {H}_1}^{+})^{q}+(\mathscr {M}_{\mathcal {H}_2}^{+})^{q}-(\mathscr {M}_{\mathcal {H}_1}^{+})^{q}(\mathscr {M}_{\mathcal {H}_2}^{+})^{q})^{\frac{1}{q}}\\ +i((\mathscr {A}_{\mathcal {H}_{1}}^{+})^{q}+(\mathscr {A}_{\mathcal {H}_{2}}^{+})^{q}-(\mathscr {A}_{\mathcal {H}_{1}}^{+})^{q} (\mathscr {A}_{\mathcal {H}_{2}}^{+})^{q})^{\frac{1}{q}},\\ (\mathscr {N}_{\mathcal {H}_1}^{+} \mathscr {N}_{\mathcal {H}_2}^{+})+i(\mathscr {B}_{\mathcal {H}_{1}}^{+}\mathscr {B}_{\mathcal {H}_{2}}^{+}),\\ -(\mathscr {M}_{\mathcal {H}_1}^{-} \mathscr {M}_{\mathcal {H}_2}^{-})+i(-(\mathscr {A}_{\mathcal {H}_{1}}^{-}\mathscr {A}_{\mathcal {H}_{2}}^{-})),\\ -(|\mathscr {N}_{\mathcal {H}_1}^{-}|^{q}+|\mathscr {N}_{\mathcal {H}_2}^{-}|^{q}-|\mathscr {N}_{\mathcal {H}_1}^{-}|^{q}|\mathscr {N}_{\mathcal {H}_2}^{-}|^{q})^{\frac{1}{q}}\\ +i(-(|\mathscr {B}_{\mathcal {H}_{1}}^{-}|^{q}+|\mathscr {B}_{\mathcal {H}_{2}}^{-}|^{q}-|\mathscr {B}_{\mathcal {H}_{1}}^{-}|^{q} |\mathscr {B}_{\mathcal {H}_{2}}^{-}|^{q})^{\frac{1}{q}}))\end{array}\right\rangle\\ & =\left\langle \begin{array}{c} (1-(1-((\mathscr {M}_{\mathcal {H}_1}^{+})^{q}+(\mathscr {M}_{\mathcal {H}_2}^{+})^{q}-(\mathscr {M}_{\mathcal {H}_1}^{+})^{q}(\mathscr {M}_{\mathcal {H}_2}^{+})^{q}))^\mathscr {I})^{\frac{1}{q}}\\ +i(1-(1-((\mathscr {A}_{\mathcal {H}_{1}}^{+})^{q}+(\mathscr {A}_{\mathcal {H}_{2}}^{+})^{q}-(\mathscr {A}_{\mathcal {H}_{1}}^{+})^{q} (\mathscr {A}_{\mathcal {H}_{2}}^{+})^{q}))^{\mathscr {I}})^{\frac{1}{q}},\\ (\mathscr {N}_{\mathcal {H}_{1}}^{+}\mathscr {N}_{\mathcal {H}_{2}}^{+})^{\mathscr {I}}+i(\mathscr {B}_{\mathcal {H}_{1}}^{+}\mathscr {B}_{\mathcal {H}_{2}}^{+})^{\mathscr {I}},\\ -(|\mathscr {M}_{\mathcal {H}_{1}}^{-}||\mathscr {M}_{\mathcal {H}_{2}}^{-}|)^{\mathscr {I}}+i(-(|\mathscr {A}_{\mathcal {H}_{1}}^{-}||\mathscr {A}_{\mathcal {H}_{2}}^{-}|)^{\mathscr {I}}),\\ -(1-(1-(-(|\mathscr {N}_{\mathcal {H}_1}^{-}|^{q}+|\mathscr {N}_{\mathcal {H}_2}^{-}|^{q}-|\mathscr {N}_{\mathcal {H}_1}^{-}|^{q}|\mathscr {N}_{\mathcal {H}_2}^{-}|^{q})))^\mathscr {I})^{\frac{1}{q}}\\ +i(-(1-(1-(-(|\mathscr {B}_{\mathcal {H}_{1}}^{-}|^{q}+|\mathscr {B}_{\mathcal {H}_{2}}^{-}|^{q}-|\mathscr {B}_{\mathcal {H}_{1}}^{-}|^{q} |\mathscr {B}_{\mathcal {H}_{2}}^{-}|^{q})))^{\mathscr {I}})^{\frac{1}{q}})\rangle \end{array}\right\rangle .\end{aligned}$$

However,

$$\begin{aligned}\mathscr {I} \mathcal {H}_1 \oplus \mathscr {I} \mathcal {H}_2=& \left\langle \begin{array}{c} 1-(1-(\mathscr {M}_{\mathcal {H}_{1}}^{+})^{q})^\mathscr {I})^{\frac{1}{q}}+i(1-(1-(\mathscr {A}_{\mathcal {H}_{1}}^{+})^{q})^{\mathscr {I}})^{\frac{1}{q}},(\mathscr {N}_{\mathcal {H}_{1}}^{+})^{\mathscr {I}}+i(\mathscr {B}_{\mathcal {H}_{1}}^{+})^{\mathscr {I}},\\ -|\mathscr {M}_{\mathcal {H}_{1}}^{-}|^{\mathscr {I}}+i(-|\mathscr {A}_{\mathcal {H}_{1}}^{-}|^{\mathscr {I}}), -(1-(1-|\mathscr {N}_{\mathcal {H}_{1}}^{-}|^{q})^\mathscr {I})^{\frac{1}{q}}+i(-(1-(1-|\mathscr {B}_{\mathcal {H}_{1}}^{-}|^{q})^{\mathscr {I}})^{\frac{1}{q}})\end{array}\right\rangle\\ &\,\,\,\,\,\,\,\,\,\,\,\,\,\,\,\,\,\,\,\,\,\,\,\,\,\,\,\,\,\,\,\,\,\,\,\,\,\,\,\,\,\,\,\,\,\,\,\,\,\,\,\,\,\,\,\,\,\,\,\,\,\,\,\,\,\,\,\,\,\,\,\,\,\,\,\,\,\,\,\,\,\,\,\,\,\,\,\,\,\,\,\,\,\,\,\,\,\, \oplus\\ & \left\langle \begin{array}{c}(1-(1-(\mathscr {M}_{\mathcal {H}_{2}}^{+})^{q})^\mathscr {I})^{\frac{1}{q}}+i(1-(1-(\mathscr {A}_{\mathcal {H}_{2}}^{+})^{q})^{\mathscr {I}})^{\frac{1}{q}},(\mathscr {N}_{\mathcal {H}_{2}}^{+})^{\mathscr {I}}+i(\mathscr {B}_{\mathcal {H}_{2}}^{+})^{\mathscr {I}},\\ -|\mathscr {M}_{\mathcal {H}_{2}}^{-}|^{\mathscr {I}}+i(-|\mathscr {A}_{\mathcal {H}_{2}}^{-}|^{\mathscr {I}}), -(1-(1-|\mathscr {N}_{\mathcal {H}_{2}}^{-}|^{q})^\mathscr {I})^{\frac{1}{q}}+i(-(1-(1-|\mathscr {B}_{\mathcal {H}_{2}}^{-}|^{q})^{\mathscr {I}})^{\frac{1}{q}})\end{array}\right\rangle\\ & =\left\langle \begin{array}{c}((1-(1-(\mathscr {M}_{\mathcal {H}_{1}}^{+})^{q})^\mathscr {I})+(1-(1-(\mathscr {M}_{\mathcal {H}_{2}}^{+})^{q})^\mathscr {I})\\ -(1-(1-(\mathscr {M}_{\mathcal {H}_{1}}^{+})^{q})^\mathscr {I})(1-(1-(\mathscr {M}_{\mathcal {H}_{2}}^{+})^{q})^\mathscr {I}))^{\frac{1}{q}}\\ +i ((1-(1-(\mathscr {A}_{\mathcal {H}_{1}}^{+})^{q})^\mathscr {I})+(1-(1-(\mathscr {A}_{\mathcal {H}_{2}}^{+})^{q})^\mathscr {I})\\ -(1-(1-(\mathscr {A}_{\mathcal {H}_{1}}^{+})^{q})^\mathscr {I})(1-(1-(\mathscr {A}_{\mathcal {H}_{2}}^{+})^{q})^\mathscr {I}))^{\frac{1}{q}},\\ (\mathscr {N}_{\mathcal {H}_{1}}^{+}\mathscr {N}_{\mathcal {H}_{2}}^{+})^{\mathscr {I}}+i(\mathscr {B}_{\mathcal {H}_{1}}^{+}\mathscr {B}_{\mathcal {H}_{2}}^{+})^{\mathscr {I}},\\ -(|\mathscr {M}_{\mathcal {H}_{1}}^{-}||\mathscr {M}_{\mathcal {H}_{2}}^{-}|)^{\mathscr {I}}+i(-(|\mathscr {A}_{\mathcal {H}_{1}}^{-}||\mathscr {A}_{\mathcal {H}_{2}}^{-}|)^{\mathscr {I}}),\\ -((1-(1-|\mathscr {N}_{\mathcal {H}_{1}}^{-}|^{q})^\mathscr {I})+(1-(1-|\mathscr {N}_{\mathcal {H}_{2}}^{-}|^{q})^\mathscr {I})\\ -(1-(1-|\mathscr {N}_{\mathcal {H}_{1}}^{-}|^{q})^\mathscr {I})(1-(1-|\mathscr {N}_{\mathcal {H}_{2}}^{-}|^{q})^\mathscr {I}))^{\frac{1}{q}}\\ +i(-((1-(1-|\mathscr {B}_{\mathcal {H}_{1}}^{-}|^{q})^\mathscr {I})+(1-(1-|\mathscr {B}_{\mathcal {H}_{2}}^{-}|^{q})^\mathscr {I})\\ -(1-(1-|\mathscr {B}_{\mathcal {H}_{1}}^{-}|^{q})^\mathscr {I})(1-(1-|\mathscr {B}_{\mathcal {H}_{2}}^{-}|^{q})^\mathscr {I}))^{\frac{1}{q}})\end{array}\right\rangle \\ & =\mathscr {I}(\mathcal {H}_1\oplus \mathcal {H}_2).\end{aligned}$$

(2)

$$\begin{aligned} \left( \mathscr {I}_1+\mathscr {I}_2\right) \mathcal {H}=&\left\langle \begin{array}{c}(1-(1-(\mathscr {M}_{\mathcal {H}}^{+})^{q})^{\mathscr {I}_{1}+\mathscr {I}_{2}})^{\frac{1}{q}}+i(1-(1-(\mathscr {A}_{\mathcal {H}}^{+})^{q})^{\mathscr {I}_{1}+\mathscr {I}_{2}})^{\frac{1}{q}},\\ (\mathscr {N}_{\mathcal {H}}^{+})^{\mathscr {I}_{1}+\mathscr {I}_{2}}+i(\mathscr {B}_{\mathcal {H}}^{+})^{\mathscr {I}_{1}+\mathscr {I}_{2}},-|\mathscr {M}_{\mathcal {H}}^{-}|^{\mathscr {I}_{1}+\mathscr {I}_{2}}+i(-|\mathscr {A}_{\mathcal {H}}^{-}|^{\mathscr {I}_{1}+\mathscr {I}_{2}}),\\ -(1-(1-|\mathscr {N}_{\mathcal {H}}^{-}|^{q})^{\mathscr {I}_{1}+\mathscr {I}_{2}})^{\frac{1}{q}}+i(-(1-(1-|\mathscr {B}_{\mathcal {H}}^{-}|^{q})^{\mathscr {I}_{1}+\mathscr {I}_{2}})^{\frac{1}{q}})\end{array}\right\rangle \end{aligned}$$

$$\begin{aligned} & =\left\langle \begin{array}{c}((1-(1-(\mathscr {M}_{\mathcal {H}}^{+})^{q})^{\mathscr {I}_{1}})+(1-(1-(\mathscr {M}_{\mathcal {H}}^{+})^{q})^{\mathscr {I}_{2}})\\ -(1-(1-(\mathscr {M}_{\mathcal {H}}^{+})^{q})^{\mathscr {I}_{1}})(1-(1-(\mathscr {M}_{\mathcal {H}}^{+})^{q})^{\mathscr {I}_{2}}))^{\frac{1}{q}}\\ +i((1-(1-(\mathscr {A}_{\mathcal {H}}^{+})^{q})^{\mathscr {I}_{1}})+(1-(1-(\mathscr {A}_{\mathcal {H}}^{+})^{q})^{\mathscr {I}_{2}})\\ -(1-(1-(\mathscr {A}_{\mathcal {H}}^{+})^{q})^{\mathscr {I}_{1}})(1-(1-(\mathscr {A}_{\mathcal {H}}^{+})^{q})^{\mathscr {I}_{2}}))^{\frac{1}{q}},\\ (\mathscr {N}_{\mathcal {H}}^{+})^{\mathscr {I}_{1}}(\mathscr {N}_{\mathcal {H}}^{+})^{\mathscr {I}_{2}}+i(\mathscr {B}_{\mathcal {H}}^{+})^{\mathscr {I}_{1}}(\mathscr {B}_{\mathcal {H}}^{+})^{\mathscr {I}_{2}},\\ -((-|\mathscr {M}_{\mathcal {H}}^{-}|^{\mathscr {I}_{1}})(-|\mathscr {M}_{\mathcal {H}}^{-}|^{\mathscr {I}_{2}}))\\ +i(-((-|\mathscr {A}_{\mathcal {H}}^{-}|^{\mathscr {I}_{1}})(-|\mathscr {A}_{\mathcal {H}}^{-}|^{\mathscr {I}_{2}}))),\\ -(|-(1-(1-|\mathscr {N}_{\mathcal {H}}^{-}|^{q})^{\mathscr {I}_{1}})|\\ +|-(1-(1-|\mathscr {N}_{\mathcal {H}}^{-}|^{q})^{\mathscr {I}_{2}})|-|-(1-(1-|\mathscr {N}_{\mathcal {H}}^{-}|^{q})^{\mathscr {I}_{1}})|\\ |-(1-(1-|\mathscr {N}_{\mathcal {H}}^{-}|^{q})^{\mathscr {I}_{2}})|)^{\frac{1}{q}}\\ +i((-(|-(1-(1-|\mathscr {B}_{\mathcal {H}}^{-}|^{q})^{\mathscr {I}_{1}})|+|-(1-(1-|\mathscr {B}_{\mathcal {H}}^{-}|^{q})^{\mathscr {I}_{2}})|-\\ |-(1-(1-|\mathscr {B}_{\mathcal {H}}^{-}|^{q})^{\mathscr {I}_{1}})||-(1-(1-|\mathscr {B}_{\mathcal {H}}^{-}|^{q})^{\mathscr {I}_{2}})|)^{\frac{1}{q}}))\end{array}\right\rangle \end{aligned}$$

$$\begin{aligned} & =\left\langle \begin{array}{c}(1-(1-(\mathscr {M}_{\mathcal {H}}^{+})^{q})^{\mathscr {I}_{1}})^{\frac{1}{q}}\\ +i(1-(1-(\mathscr {A}_{\mathcal {H}}^{+})^{q})^{\mathscr {I}_{1}})^{\frac{1}{q}},(\mathscr {N}_{\mathcal {H}}^{+})^{\mathscr {I}_{1}}\\ +i(\mathscr {B}_{\mathcal {H}}^{+})^{\mathscr {I}_{1}},\\ -|\mathscr {M}_{\mathcal {H}}^{-}|^{\mathscr {I}_{1}}+i(-|\mathscr {A}_{\mathcal {H}}^{-}|^{\mathscr {I}_{1}}),\\ -(1-(1-|\mathscr {N}_{\mathcal {H}}^{-}|^{q})^{\mathscr {I}_{1}})^{\frac{1}{q}}\\ +i(-(1-(1-|\mathscr {B}_{\mathcal {H}}^{-}|^{q})^{\mathscr {I}_{1}})^{\frac{1}{q}})\end{array}\right\rangle \\ &\,\,\,\,\,\,\,\,\,\,\,\,\,\,\,\,\,\,\,\,\,\,\,\,\,\,\,\,\,\,\,\,\,\,\,\,\,\,\,\,\,\,\,\,\,\,\,\,\,\,\,\,\, \oplus \end{aligned}$$

$$\begin{aligned}&\left\langle \begin{array}{c}(1-(1-(\mathscr {M}_{\mathcal {H}}^{+})^{q})^{\mathscr {I}_{2}})^{\frac{1}{q}}\\ +i(1-(1-(\mathscr {A}_{\mathcal {H}}^{+})^{q})^{\mathscr {I}_{2}})^{\frac{1}{q}},(\mathscr {N}_{\mathcal {H}}^{+})^{\mathscr {I}_{2}}+i(\mathscr {B}_{\mathcal {H}}^{+})^{\mathscr {I}_{2}},\\ -|\mathscr {M}_{\mathcal {H}}^{-}|^{\mathscr {I}_{2}}+i(-|\mathscr {A}_{\mathcal {H}}^{-}|^{\mathscr {I}_{2}}), \\ -(1-(1-|\mathscr {N}_{\mathcal {H}}^{-}|^{q})^{\mathscr {I}_{2}})^{\frac{1}{q}}+i(-(1-(1-|\mathscr {B}_{\mathcal {H}}^{-}|^{q})^{\mathscr {I}_{2}})^{\frac{1}{q}})\end{array}\right\rangle\\ &=\mathscr {I}_1 \mathcal {H} \oplus \mathscr {I}_2 \mathcal {H}. \end{aligned}$$

$$\square$$

## BCq-ROF aggregation operators

This part is dedicated to exploring the utilization of BCq-ROF weighted average and geometric aggregation operators for handling and analyzing data within the framework of BCq-ROFS. A comprehensive discussion is provided, highlighting the mathematical principles underlying these aggregation techniques and their significance in decision-making processes.

### Definition 4.1

Let us consider a set of BCq-ROFNs, symbolized as:

$$\mathcal {H}_{i} = \left\{ \left\langle \mathscr {M}_{\mathcal {H}_{i}}^{+} + i \mathscr {A}_{\mathcal {H}_{i}}^{+}, \mathscr {N}_{\mathcal {H}_{i}}^{+} + i \mathscr {B}_{\mathcal {H}_{i}}^{+}, \mathscr {M}_{\mathcal {H}_{i}}^{-} + i \mathscr {A}_{\mathcal {H}_{i}}^{-}, \mathscr {N}_{\mathcal {H}_{i}}^{-} + i \mathscr {B}_{\mathcal {H}_{i}}^{-} \right\rangle \right\} , \quad \forall i \in \{1,2,\dots ,k\}$$

where each $\mathcal {H}_i$ represents an individual BCq-ROFN. Additionally, we define a weight vector $\mathscr {X}$ associated with these elements, given by:

$$\mathscr {X} = \left( \mathscr {X}_1, \mathscr {X}_2, \dots , \mathscr {X}_k\right) ^T$$

where each weight component satisfies $\mathscr {X}_i > 0$ and collectively they sum up to unity, that is, $\sum _{i=1}^{k} \mathscr {X}_i = 1$. Based on this formulation, we introduce two fundamental aggregation operators:

1. The BCq-ROF weighted averaging (BCq-ROFWA) operator is a mapping BCq-ROFWA$: \mathcal {H}^k \rightarrow \mathcal {H}$ which aggregates the given BCq-ROFNs using a weighted averaging approach. It is mathematically expressed as:
  8 $$\begin{aligned} BCq-ROFWA\left( \mathcal {H}_1, \mathcal {H}_2, \ldots , \mathcal {H}_k\right) =\bigoplus _{i=1}^k \mathscr {X}_i \mathcal {H}_i=\mathscr {X}_1 \mathcal {H}_1 \oplus \mathscr {X}_2 \mathcal {H}_2 \oplus \cdots \oplus \mathscr {X}_k \mathcal {H}_k. \end{aligned}$$
2. The BCq-ROF weighted geometric (BCq-ROFWG) operator provides an alternative aggregation mechanism by employing a weighted geometric approach. It is defined as a mapping BCq-ROFWG$: \mathcal {H}^k \rightarrow \mathcal {H}$, and its functional form is given by:
  9 $$\begin{aligned} BCq-ROFWG\left( \mathcal {H}_1, \mathcal {H}_2, \ldots , \mathcal {H}_k\right) =\bigotimes _{i=1}^k \mathcal {H}_i^{\mathscr {X}_i}=\mathcal {H}_1^{\mathscr {X}_1} \otimes \mathcal {H}_2^{\mathscr {X}_2} \otimes \cdots \otimes \mathcal {H}_k^{\mathscr {X}_k}. \end{aligned}$$
  This operation applies a weighted geometric combination of the given BCq-ROFNs, effectively capturing a different mode of aggregation compared to the averaging approach.

### Theorem 4.2

Let $\mathcal {H}_i$ represent a collection of BCq-ROFNs, where each element is defined as:

$$\mathcal {H}_{i} = \left\langle \mathscr {M}_{\mathcal {H}_{i}}^{+} + i \mathscr {A}_{\mathcal {H}_{i}}^{+}, \mathscr {N}_{\mathcal {H}_{i}}^{+} + i \mathscr {B}_{\mathcal {H}_{i}}^{+}, \mathscr {M}_{\mathcal {H}_{i}}^{-} + i \mathscr {A}_{\mathcal {H}_{i}}^{-}, \mathscr {N}_{\mathcal {H}_{i}}^{-} + i \mathscr {B}_{\mathcal {H}_{i}}^{-} \right\rangle , \quad i=1,2,\dots ,k.$$

Furthermore, let $\mathscr {X} = \left( \mathscr {X}_1, \mathscr {X}_2, \dots , \mathscr {X}_k \right) ^T$ be the corresponding weight vector, where each weight satisfies $\mathscr {X}_i > 0$ and the sum constraint $\sum _{i=1}^{k} \mathscr {X}_i = 1$. Then, the BCq-ROFW averaging and geometric aggregation operators can be alternatively formulated as follows:

1. The BCq-ROFWA operator aggregates the given BCq-ROFNs based on a weighted averaging approach, and its alternative formulation is:
  10 $$\begin{aligned} \operatorname {BCq-ROFWA}\left( \mathcal {H}_1, \mathcal {H}_2, \ldots , \mathcal {H}_k\right) =\left\langle \begin{array}{c} (1-{\prod }_{i=1}^k(1-(\mathscr {M}_{\mathcal {H}_i}^{+})^{q})^{\mathscr {X}_i})^{\frac{1}{q}}+i(1-{\prod }_{i=1}^k(1-(\mathscr {A}_{\mathcal {H}_i}^{+})^{q})^{\mathscr {X}_i})^{\frac{1}{q}}, \\ {\prod }_{i=1}^k(\mathscr {N}_{\mathcal {H}_i}^{+})^{\mathscr {X}_i}+i{\prod }_{i=1}^k(\mathscr {B}_{\mathcal {H}_i}^{+})^{\mathscr {X}_i},\\ -({\prod }_{i=1}^k|\mathscr {M}_{\mathcal {H}_i}^{-}|^{\mathscr {X}_i})+i(-({\prod }_{i=1}^k|\mathscr {A}_{\mathcal {H}_i}^{-}|^{\mathscr {X}_i})) \\ -(1-{\prod }_{i=1}^k(1-|\mathscr {N}_{\mathcal {H}_i}^{-}|^{q})^{\mathscr {X}_i})^{\frac{1}{q}}+i(-(1-{\prod }_{i=1}^k(1-|\mathscr {B}_{\mathcal {H}_i}^{-}|^{q})^{\mathscr {X}_i})^{\frac{1}{q}})\end{array}\right\rangle \end{aligned}$$
2. The BCq-ROFWG operator follows a geometric-based aggregation method, and its alternative formulation is given by:
  11 $$\begin{aligned} \operatorname {BCq-ROFWG}\left( \mathcal {H}_1, \mathcal {H}_2, \ldots , \mathcal {H}_k\right) = \left\langle \begin{array}{c}{\prod }_{i=1}^k(\mathscr {M}_{\mathcal {H}_i}^{+})^{\mathscr {X}_i}+i({\prod }_{i=1}^k\mathscr {A}_{\mathcal {H}_i}^{+})^{\mathscr {X}_{i}},\\ (1-{\prod }_{i=1}^k(1-(\mathscr {N}_{\mathcal {H}_i}^{+})^{q})^{\mathscr {X}_i})^{\frac{1}{q}}+i(1-{\prod }_{i=1}^k(1-(\mathscr {B}_{\mathcal {H}_i}^{+})^{q})^{\mathscr {X}_i})^{\frac{1}{q}}, \\ -(1-{\prod }_{i=1}^k(1-|\mathscr {M}_{\mathcal {H}_i}^{-}|^{q})^{\mathscr {X}_i})^{\frac{1}{q}}+i (-(1-{\prod }_{i=1}^k(1-|\mathscr {A}_{\mathcal {H}_i}^{-}|^{q})^{\mathscr {X}_i})^{\frac{1}{q}}),\\ -({\prod }_{i=1}^k|\mathscr {N}_{\mathcal {H}_i}^{-}|_i^{\mathscr {X}_i})+i(-({\prod }_{i=1}^k|\mathscr {B}_{\mathcal {H}_i}^{-}|_i^{\mathscr {X}_i}))\end{array}\right\rangle \end{aligned}$$

### Proof

1. To confirm the validity of the result using mathematical induction, we begin with the base case where $k=2$. At this stage, the given formula simplifies, enabling us to verify its correctness for the smallest instance. This step lays the groundwork for the induction process, which will subsequently be extended to greater values of *k*. The formula reduces to the following form:
  $$\begin{aligned} \operatorname {BCq-ROFWA}\left( \mathcal {H}_1, \mathcal {H}_2\right) = \mathscr {X}_1 \mathcal {H}_1 \oplus \mathscr {X}_2 \mathcal {H}_2= \end{aligned}$$
  $$\begin{gathered} \left\langle {\begin{array}{*{20}c} {(1 - (1 - ({\mathcal{M}}_{{{\mathcal{H}}_{1} }}^{ + } )^{q} )^{{{\mathcal{X}}_{1} }} )^{{\frac{1}{q}}} + i(1 - (1 - ({\mathcal{A}}_{{{\mathcal{H}}_{1} }}^{ + } )^{q} )^{{{\mathcal{X}}_{1} }} )^{{\frac{1}{q}}} ,} \\ {({\mathcal{N}}_{{{\mathcal{H}}_{1} }}^{ + } )^{{{\mathcal{X}}_{1} }} + i({\mathcal{B}}_{{{\mathcal{H}}_{1} }}^{ + } )^{{{\mathcal{X}}_{1} }} , - |{\mathcal{M}}_{{{\mathcal{H}}_{1} }}^{ - } |^{{{\mathcal{X}}_{1} }} + i( - |{\mathcal{A}}_{{{\mathcal{H}}_{1} }}^{ - } |^{{{\mathcal{X}}_{1} }} ),} \\ { - (1 - (1 - |{\mathcal{N}}_{{{\mathcal{H}}_{1} }}^{ - } |^{q} )^{{{\mathcal{X}}_{1} }} )^{{\frac{1}{q}}} + i( - (1 - (1 - |{\mathcal{B}}_{{{\mathcal{H}}_{1} }}^{ - } |^{q} )^{{{\mathcal{X}}_{1} }} )^{{\frac{1}{q}}} )} \\ \end{array} } \right\rangle \hfill \\ \,\,\,\,\,\,\,\,\,\,\,\,\,\,\,\,\,\,\,\,\,\,\,\,\,\,\,\,\,\,\,\,\,\,\,\,\,\,\,\,\,\,\,\,\,\,\,\,\,\,\,\,\,\,\,\,\,\,\,\, \oplus \hfill \\ \end{gathered}$$
  $$\left\langle \begin{array}{c}(1-(1-(\mathscr {M}_{\mathcal {H}_{2}}^{+})^{q})^{\mathscr {X}_{2}})^{\frac{1}{q}}+i(1-(1-(\mathscr {A}_{\mathcal {H}_{2}}^{+})^{q})^{\mathscr {X}_{2}})^{\frac{1}{q}},\\ (\mathscr {N}_{\mathcal {H}_{2}}^{+})^{\mathscr {X}_{2}}+i(\mathscr {B}_{\mathcal {H}_{2}}^{+})^{\mathscr {X}_{2}},-|\mathscr {M}_{\mathcal {H}_{2}}^{-}|^{\mathscr {X}_{2}}+i(-|\mathscr {A}_{\mathcal {H}_{2}}^{-}|^{\mathscr {X}_{2}}), \\ -(1-(1-|\mathscr {N}_{\mathcal {H}_{2}}^{-}|^{q})^{\mathscr {X}_{2}})^{\frac{1}{q}}+i(-(1-(1-|\mathscr {B}_{\mathcal {H}_{2}}^{-}|^{q})^{\mathscr {X}_{2}})^{\frac{1}{q}})\end{array}\right\rangle$$
  $$=\left\langle \begin{array}{c}((1-(1-(\mathscr {M}_{\mathcal {H}_{1}}^{+})^{q})^{\mathscr {X}_{1}})+(1-(1-(\mathscr {M}_{\mathcal {H}_{2}}^{+})^{q})^{\mathscr {X}_{2}})\\ -(1-(1-(\mathscr {M}_{\mathcal {H}_{1}}^{+})^{q})^{\mathscr {X}_{1}})(1-(1-(\mathscr {M}_{\mathcal {H}_{2}}^{+})^{q})^{\mathscr {X}_{2}}))^{\frac{1}{q}}\\ +i ((1-(1-(\mathscr {A}_{\mathcal {H}_{1}}^{+})^{q})^{\mathscr {X}_{1}})+(1-(1-(\mathscr {A}_{\mathcal {H}_{2}}^{+})^{q})^{\mathscr {X}_{2}})\\ -(1-(1-(\mathscr {A}_{\mathcal {H}_{1}}^{+})^{q})^{\mathscr {X}_{1}})(1-(1-(\mathscr {A}_{\mathcal {H}_{2}}^{+})^{q})^{\mathscr {X}_{2}}))^{\frac{1}{q}},\\ (\mathscr {N}_{\mathcal {H}_{1}}^{+})^{\mathscr {X}_{1}}(\mathscr {N}_{\mathcal {H}_{2}}^{+})^{\mathscr {X}_{2}}\\ +i(\mathscr {B}_{\mathcal {H}_{1}}^{+})^{\mathscr {X}_{1}}(\mathscr {B}_{\mathcal {H}_{2}}^{+})^{\mathscr {X}_{2}},\\ -(|\mathscr {M}_{\mathcal {H}_{1}}^{-}|^{\mathscr {X}_{1}}|\mathscr {M}_{\mathcal {H}_{2}}^{-}|^{\mathscr {X}_{2}}) \\ +i(-(|\mathscr {A}_{\mathcal {H}_{1}}^{-}|^{\mathscr {X}_{1}}|\mathscr {A}_{\mathcal {H}_{2}}^{-}|^{\mathscr {X}_{2}})),\\ -((1-(1-|\mathscr {N}_{\mathcal {H}_{1}}^{-}|^{q})^{\mathscr {X}_{1}})+(1-(1-|\mathscr {N}_{\mathcal {H}_{2}}^{-}|^{q})^{\mathscr {X}_{2}})\\ -(1-(1-|\mathscr {N}_{\mathcal {H}_{1}}^{-}|^{q})^{\mathscr {X}_{1}})(1-(1-|\mathscr {N}_{\mathcal {H}_{2}}^{-}|^{q})^{\mathscr {X}_{2}}))^{\frac{1}{q}}\\ +i(-((1-(1-|\mathscr {B}_{\mathcal {H}_{1}}^{-}|^{q})^{\mathscr {X}_{1}})+(1-(1-|\mathscr {B}_{\mathcal {H}_{2}}^{-}|^{q})^{\mathscr {X}_{2}})\\ -(1-(1-|\mathscr {B}_{\mathcal {H}_{1}}^{-}|^{q})^{\mathscr {X}_{1}})(1-(1-|\mathscr {B}_{\mathcal {H}_{2}}^{-}|^{q})^{\mathscr {X}_{2}}))^{\frac{1}{q}})\end{array}\right\rangle$$
  $$=\left\langle \begin{array}{c}(1-(1-(\mathscr {M}_{\mathcal {H}_1}^{+})^{q})^{\mathscr {X}_1} (1-(\mathscr {M}_{\mathcal {H}_2}^{+})^{q})^{\mathscr {X}_2})^{\frac{1}{q}}\\ +i(1-(1-(\mathscr {A}_{\mathcal {H}_1}^{+})^{q})^{\mathscr {X}_1} (1-(\mathscr {A}_{\mathcal {H}_2}^{+})^{q})^{\mathscr {X}_2})^{\frac{1}{q}}, \\ (\mathscr {N}_{\mathcal {H}_1}^{+})^{\mathscr {X}_1} (\mathscr {N}_{\mathcal {H}_2}^{+})^{\mathscr {X}_2}\\ +i((\mathscr {B}_{\mathcal {H}_1}^{+})^{\mathscr {X}_1}(\mathscr {B}_{\mathcal {H}_2}^{+})^{\mathscr {X}_2}),-(( |\mathscr {M}_{\mathcal {H}_1}^{-}|^{\mathscr {X}_1})(|\mathscr {M}_{\mathcal {H}_2}^{-}|^{\mathscr {X}_2}))\\ +i(-(( |\mathscr {A}_{\mathcal {H}_1}^{-}|^{\mathscr {X}_1})(|\mathscr {A}_{\mathcal {H}_2}^{-}|^{\mathscr {X}_2}))),\\ -(1-(1- |\mathscr {N}_{\mathcal {H}_1}^{-}|^{q})^{\mathscr {X}_1} (1-|\mathscr {N}_{\mathcal {H}_2}^{-}|^{q})^{\mathscr {X}_2})^{\frac{1}{q}}\\ +i(-(1-(1-|\mathscr {B}_{\mathcal {H}_1}^{-}|^{q})^{\mathscr {X}_1} (1-|\mathscr {B}_{\mathcal {H}_2}^{-}|^{q})^{\mathscr {X}_2})^{\frac{1}{q}})\end{array}\right\rangle$$
  $$\,\,\,\,\,\,\,=\left\langle \begin{array}{c}( 1-{\prod }_{i=1}^2(1-(\mathscr {M}_{\mathcal {H}_i}^{+})^{q})^{\mathscr {X}_i})^{\frac{1}{q}}+i( 1-{\prod }_{i=1}^2(1- (\mathscr {A}_{\mathcal {H}_i}^{+})^{q})^{\mathscr {X}_i})^{\frac{1}{q}},\\ {\prod }_{i=1}^2(\mathscr {N}_{\mathcal {H}_i}^{+})^{\mathscr {X}_i}+i{\prod }_{i=1}^2(\mathscr {B}_{\mathcal {H}_i}^{+})^{\mathscr {X}_i},\\ -({\prod }_{i=1}^2|\mathscr {M}_{\mathcal {H}_i}^{-}|^{\mathscr {X}_i})+i(-({\prod }_{i=1}^2|\mathscr {A}_{\mathcal {H}_i}^{-}|^{\mathscr {X}_i})),\\ -(1- {\prod }_{i=1}^2(1-|\mathscr {N}_{\mathcal {H}_i}^{-}|^{q})^{\mathscr {X}_i})^{\frac{1}{q}}\\ +i(-(1-{\prod }_{i=1}^2(1-|\mathscr {B}_{\mathcal {H}_i}^{-}|^{q})^{\mathscr {X}_i})^{\frac{1}{q}})\end{array}\right\rangle .$$
  Supposing the theorem is true for $k=l$, it follows that the statement holds for *l* elements. In particular, we assume:
  $$\operatorname {BCq-ROFWA}(\mathcal {H}_1, \mathcal {H}_2, \ldots , \mathcal {H}_l)= \mathscr {X}_1 \mathcal {H}_1 \oplus \mathscr {X}_2 \mathcal {H}_2 \oplus \cdots \oplus \mathscr {X}_l \mathcal {H}_l$$
  $$=\left\langle \begin{array}{c}( 1-{\prod }_{i=1}^l(1- (\mathscr {M}_{\mathcal {H}_i}^{+})^{q})^{\mathscr {X}_i})^{\frac{1}{q}}+i( 1-{\prod }_{i=1}^l(1- (\mathscr {A}_{\mathcal {H}_i}^{+})^{q})^{\mathscr {X}_i})^{\frac{1}{q}},\\ {\prod }_{i=1}^l(\mathscr {N}_{\mathcal {H}_i}^{+})^{\mathscr {X}_i}+i{\prod }_{i=1}^l(\mathscr {B}_{\mathcal {H}_i}^{+})^{\mathscr {X}_i},-({\prod }_{i=1}^l|\mathscr {M}_{\mathcal {H}_i}^{-}|^{\mathscr {X}_i})+i(-({\prod }_{i=1}^l|\mathscr {A}_{\mathcal {H}_i}^{-}|^{\mathscr {X}_i})),\\ -(1- {\prod }_{i=1}^l(1-|\mathscr {N}_{\mathcal {H}_i}^{-}|^{q})^{\mathscr {X}_i})^{\frac{1}{q}}+i(-(1- {\prod }_{i=1}^l(1-|\mathscr {B}_{\mathcal {H}_i}^{-}|^{q})^{\mathscr {X}_i})^{\frac{1}{q}})\end{array}\right\rangle .$$
  To establish the validity of the statement for $k=l+1$, we start by applying the inductive hypothesis. In this case, for $k=l+1$, the expression transforms into:
  $$\operatorname {BCq-ROFWA}(\mathcal {H}_1, \mathcal {H}_2, \ldots , \mathcal {H}_{l+1})=\mathscr {X}_1 \mathcal {H}_1 \oplus \mathscr {X}_2 \mathcal {H}_2 \oplus \cdots \oplus \mathscr {X}_{l+1} \mathcal {H}_{l+1}$$
  $$=\left\langle \begin{array}{c}( 1-{\prod }_{i=1}^l(1- (\mathscr {M}_{\mathcal {H}_i}^{+})^{q})^{\mathscr {X}_i})^{\frac{1}{q}}+i( 1-{\prod }_{i=1}^l(1- (\mathscr {A}_{\mathcal {H}_i}^{+})^{q})^{\mathscr {X}_i})^{\frac{1}{q}},\\ {\prod }_{i=1}^l(\mathscr {N}_{\mathcal {H}_i}^{+})^{\mathscr {X}_i}+i{\prod }_{i=1}^l(\mathscr {B}_{\mathcal {H}_i}^{+})^{\mathscr {X}_i},-({\prod }_{i=1}^l|\mathscr {M}_{\mathcal {H}_i}^{-}|^{\mathscr {X}_i})+i(-({\prod }_{i=1}^l|\mathscr {A}_{\mathcal {H}_i}^{-}|^{\mathscr {X}_i})),\\ -(1- {\prod }_{i=1}^l(1-|\mathscr {N}_{\mathcal {H}_i}^{-}|^{q})^{\mathscr {X}_i})^{\frac{1}{q}}+i(-(1- {\prod }_{i=1}^l(1-|\mathscr {B}_{\mathcal {H}_i}^{-}|^{q})^{\mathscr {X}_i})^{\frac{1}{q}})\end{array}\right\rangle$$
  $$\oplus$$
  $$\left\langle \begin{array}{c}(1-(1-(\mathscr {M}_{\mathcal {H}_{l+1}}^{+})^{q})^{\mathscr {X}_{l+1}})^{\frac{1}{q}}\\ +i(1-(1-(\mathscr {A}_{\mathcal {H}_{l+1}}^{+})^{q})^{\mathscr {X}_{l+1}})^{\frac{1}{q}},\\ (\mathscr {N}_{\mathcal {H}_{l+1}}^{+})^{\mathscr {X}_{l+1}}+i(\mathscr {B}_{\mathcal {H}_{l+1}}^{+})^{\mathscr {X}_{l+1}},\\ -|\mathscr {M}_{\mathcal {H}_{l+1}}^{-}|^{\mathscr {X}_{l+1}}+i(-|\mathscr {A}_{\mathcal {H}_{l+1}}^{-}|^{\mathscr {X}_{l+1}}), \\ -(1-(1-|\mathscr {N}_{\mathcal {H}_{l+1}}^{-}|^{q})^{\mathscr {X}_{l+1}})^{\frac{1}{q}}\\ +i(-(1-(1-|\mathscr {B}_{\mathcal {H}_{l+1}}^{-}|^{q})^{\mathscr {X}_{l+1}})^{\frac{1}{q}})\end{array}\right\rangle$$
  $$=\left\langle \begin{array}{c}(( 1-{\prod }_{i=1}^l(1- (\mathscr {M}_{\mathcal {H}_i}^{+})^{q})^{\mathscr {X}_i})+(1-(1-(\mathscr {M}_{\mathcal {H}_{l+1}}^{+})^{q})^{\mathscr {X}_{l+1}})-\\ ( 1-{\prod }_{i=1}^l(1- (\mathscr {M}_{\mathcal {H}_i}^{+})^{q})^{\mathscr {X}_i})(1-(1-(\mathscr {M}_{\mathcal {H}_{l+1}}^{+})^{q})^{\mathscr {X}_{l+1}}))^\frac{1}{q}+\\ i(( 1-{\prod }_{i=1}^l(1- (\mathscr {A}_{\mathcal {H}_i}^{+})^{q})^{\mathscr {X}_i})+(1-(1-(\mathscr {A}_{\mathcal {H}_{l+1}}^{+})^{q})^{\mathscr {X}_{l+1}})-\\ ( 1-{\prod }_{i=1}^l(1- (\mathscr {A}_{\mathcal {H}_i}^{+})^{q})^{\mathscr {X}_i})(1-(1-(\mathscr {A}_{\mathcal {H}_{l+1}}^{+})^{q})^{\mathscr {X}_{l+1}}))^\frac{1}{q},\\ ({\prod }_{i=1}^l(\mathscr {N}_{\mathcal {H}_i}^{+})^{\mathscr {X}_i}(\mathscr {N}_{\mathcal {H}_{l+1}}^{+})^{\mathscr {X}_{l+1}})\\ +i({\prod }_{i=1}^l(\mathscr {B}_{\mathcal {H}_i}^{+})^{\mathscr {X}_i}(\mathscr {B}_{\mathcal {H}_{l+1}}^{+})^{\mathscr {X}_{l+1}}),\\ -((-({\prod }_{i=1}^l|\mathscr {M}_{\mathcal {H}_i}^{-}|^{\mathscr {X}_i}))(-|\mathscr {M}_{\mathcal {H}_{l+1}}^{-}|^{\mathscr {X}_{l+1}}))\\ +i(-((-({\prod }_{i=1}^l|\mathscr {A}_{\mathcal {H}_i}^{-}|^{\mathscr {X}_i}))(-|\mathscr {A}_{\mathcal {H}_{l+1}}^{-}|^{\mathscr {X}_{l+1}}))),\\ -(|-(1- {\prod }_{i=1}^l(1-|\mathscr {N}_{\mathcal {H}_i}^{-}|^{q})^{\mathscr {X}_i})|+(| -(1-(1-|\mathscr {N}_{\mathcal {H}_{l+1}}^{-}|^{q})^{\mathscr {X}_{l+1}})|)-\\ (|-(1- {\prod }_{i=1}^l(1-|\mathscr {N}_{\mathcal {H}_i}^{-}|^{q})^{\mathscr {X}_i})|)(| -(1-(1-|\mathscr {N}_{\mathcal {H}_{l+1}}^{-}|^{q})^{\mathscr {X}_{l+1}})|))^{\frac{1}{q}}+\\ i(-(|-(1- {\prod }_{i=1}^l(1-|\mathscr {B}_{\mathcal {H}_i}^{-}|^{q})^{\mathscr {X}_i})|+(| -(1-(1-|\mathscr {B}_{\mathcal {H}_{l+1}}^{-}|^{q})^{\mathscr {X}_{l+1}})|)-\\ (|-(1- {\prod }_{i=1}^l(1-|\mathscr {B}_{\mathcal {H}_i}^{-}|^{q})^{\mathscr {X}_i})|)(| -(1-(1-|\mathscr {B}_{\mathcal {H}_{l+1}}^{-}|^{q})^{\mathscr {X}_{l+1}})|))^{\frac{1}{q}})\end{array}\right\rangle$$
  $$= \left\langle \begin{array}{c}( 1-{\prod }_{i=1}^{l+1}(1- (\mathscr {M}_{\mathcal {H}_i}^{+})^{q})^{\mathscr {X}_i})^{\frac{1}{q}}\\ +i( 1-{\prod }_{i=1}^{l+1}(1- (\mathscr {A}_{\mathcal {H}_i}^{+})^{q})^{\mathscr {X}_i})^{\frac{1}{q}},\\ {\prod }_{i=1}^{l+1}(\mathscr {N}_{\mathcal {H}_i}^{+})^{\mathscr {X}_i}+i{\prod }_{i=1}^{l+1}(\mathscr {B}_{\mathcal {H}_i}^{+})^{\mathscr {X}_i},\\ -({\prod }_{i=1}^{l+1}|\mathscr {M}_{\mathcal {H}_i}^{-}|^{\mathscr {X}_i})+i(-({\prod }_{i=1}^{l+1}|\mathscr {A}_{\mathcal {H}_i}^{-}|^{\mathscr {X}_i})),\\ -(1- {\prod }_{i=1}^{l+1}(1-|\mathscr {N}_{\mathcal {H}_i}^{-}|^{q})^{\mathscr {X}_i})^{\frac{1}{q}}\\ +i(-(1- {\prod }_{i=1}^{l+1}(1-|\mathscr {B}_{\mathcal {H}_i}^{-}|^{q})^{\mathscr {X}_i})^{\frac{1}{q}})\end{array}\right\rangle .$$
  This is consistent with the formula for $k = l + 1$, thus concluding the proof by induction.
2. The proof is conducted following the approach used in proving (1).

### Example 4.3

Consider the following BCq-ROFNs:

$$\mathcal {H}_1=\langle .22 +i(.86),.75 +i (.12), -.91 +i (-.54), -.03 +i (-.32)\rangle$$

$$\mathcal {H}_2=\langle .81 +i(.75),.14 +i (.13), -.46 +i (-.62), -.51 +i (-.33)\rangle$$

and

$$\mathcal {H}_3=\langle .54 +i(.23),.29 +i (.35), -.81 +i (-.26), -.14 +i (-.73)\rangle$$,

with the associated weight vector $\mathscr {X}=(.375,.291,.334)^T$ respectively. Then, we proceed as follows:

1. $$\mathrm{BCq} - ROFWA({\mathcal{H}}_{1} ,{\mathcal{H}}_{2} ,{\mathcal{H}}_{3} ) = \left\langle {\begin{array}{*{20}l} {(1 - \prod _{{i = 1}}^{3} (1 - ({\mathcal{M}}_{{{\mathcal{H}}_{i} }}^{ + } )^{q} )^{{{\mathcal{X}}_{i} }} )^{{\frac{1}{q}}} + i(1 - \prod _{{i = 1}}^{3} (1 - ({\mathcal{A}}_{{{\mathcal{H}}_{i} }}^{ + } )^{q} )^{{{\mathcal{X}}_{i} }} )^{{\frac{1}{q}}} ,} \hfill \\ {\prod _{{i = 1}}^{3} ({\mathcal{N}}_{{{\mathcal{H}}_{i} }}^{ + } )^{{{\mathcal{X}}_{i} }} + i\prod _{{i = 1}}^{3} ({\mathcal{B}}_{{{\mathcal{H}}_{i} }}^{ + } )^{{{\mathcal{X}}_{i} }} ,} \hfill \\ { - (\prod _{{i = 1}}^{3} |{\mathcal{M}}_{{{\mathcal{H}}_{i} }}^{ - } |^{{{\mathcal{X}}_{i} }} ) + i( - (\prod _{{i = 1}}^{3} |{\mathcal{A}}_{{{\mathcal{H}}_{i} }}^{ - } |^{{{\mathcal{X}}_{i} }} )),} \hfill \\ { - (1 - \prod _{{i = 1}}^{3} (1 - |{\mathcal{N}}_{{{\mathcal{H}}_{i} }}^{ - } |^{q} )^{{{\mathcal{X}}_{i} }} )^{{\frac{1}{q}}} + i( - (1 - \prod _{{i = 1}}^{3} (1 - |{\mathcal{B}}_{{{\mathcal{H}}_{i} }}^{ - } |^{q} )^{{{\mathcal{X}}_{i} }} )^{{\frac{1}{q}}} )} \hfill \\ \end{array} } \right\rangle$$
  $$\approx \left\{ \begin{array}{llllllllll} \langle .5665 +.7071 i,.3351 +.1756 i, -.7177 +(-.4404)i, -.2361 +(-.5027)i\rangle & \\ \text{ for } q= 1,\\ \langle .5990 +.7307 i,.3351 +.1756 i, -.7177 +(-.4404)i, -.3005 +(-.5290)i\rangle & \\ \text{ for } q= 2,\\ \langle .6263 +.7481 i,.3351 +.1756 i, -.7177 +(-.4404)i, -.3461 +(-.5552)i\rangle & \\ \text{ for } q= 3,\\ \langle .6483 +.7610 i,.3351 +.1756 i, -.7177 +(-.4404)i, -.3775 +(-.5787)i\rangle & \\ \text{ for } q= 4,\\ \langle .6662 +.7707 i,.3351 +.1756 i, -.7177 +(-.4404)i, -.3996 +(-.5986)i\rangle & \\ \text{ for } q= 5. \end{array} \right.$$
2. $$\operatorname {BCq-ROFWG}\left( \mathcal {H}_1, \mathcal {H}_2, \mathcal {H}_3\right) = \left\langle \begin{array}{c}{\prod }_{i=1}^3(\mathscr {M}_{\mathcal {H}_i}^{+})^{\mathscr {X}_i}+i({\prod }_{i=1}^3\mathscr {A}_{\mathcal {H}_i}^{+})^{\mathscr {X}_{i}},\\ (1-{\prod }_{i=1}^3(1-(\mathscr {N}_{\mathcal {H}_i}^{+})^{q})^{\mathscr {X}_i})^{\frac{1}{q}}+i(1-{\prod }_{i=1}^3(1-(\mathscr {B}_{\mathcal {H}_i}^{+})^{q})^{\mathscr {X}_i})^{\frac{1}{q}}, \\ -(1-{\prod }_{i=1}^3(1-|\mathscr {M}_{\mathcal {H}_i}^{-}|^{q})^{\mathscr {X}_i})^{\frac{1}{q}}+i (-(1-{\prod }_{i=1}^3(1-|\mathscr {A}_{\mathcal {H}_i}^{-}|^{q})^{\mathscr {X}_i})^{\frac{1}{q}}),\\ -({\prod }_{i=1}^3|\mathscr {N}_{\mathcal {H}_i}^{-}|_i^{\mathscr {X}_i})+i(-({\prod }_{i=1}^3|\mathscr {B}_{\mathcal {H}_i}^{-}|_i^{\mathscr {X}_i}))\end{array}\right\rangle$$
  $$\approx \left\{ \begin{array}{llllllllll} \langle .4339 +.5320 i,.4924 +.2073 i, -.8054 +(-.4900)i, -.1145 +(-.4253)i\rangle & \\ \text{ for } \,q= 1,\\ \langle .4339 +.5320 i,.5402 +.2294 i, -.8139 +(-.5046)i, -.1145 +(-.4253)i\rangle & \\ \text{ for } \,q= 2,\\ \langle .4339 +.5320 i,.5780 +.2509 i, -.8215 +(-.5177)i, -.1145 +(-.4253)i\rangle & \\ \text{ for }\, q= 3,\\ \langle .4339 +.5320 i,.6063 +.2685 i, -.8281 +(-.5287)i, -.1145 +(-.4253)i\rangle & \\ \text{ for }\, q= 4,\\ \langle .4339 +.5320 i,.6274 +.2818 i, -.8339 +(-.5377)i, -.1145 +(-.4253)i\rangle & \\ \text{ for }\, q= 5. \end{array} \right.$$

### Theorem 4.4

The results obtained by applying the operators $\operatorname {BCq-ROFWA}\left( \mathcal {H}_1, \mathcal {H}_2, \ldots , \mathcal {H}_k\right)$   and   $\operatorname {BCq-ROFWG}\left( \mathcal {H}_1, \mathcal {H}_2, \ldots , \mathcal {H}_k\right)$ are both classified as BCq-ROFNs.

### Proof

The conclusion that applying the $\operatorname {BCq-ROFWA}$ and $\operatorname {BCq-ROFWG}$ operators yields BCq-ROFNs is directly supported by Theorems 3.10 and 3.11. $\square$

### Theorem 4.5

(Idempotency) A set of BCq-ROFNs is defined as:

$\mathcal {H}_{i}=\left\{ \left\langle \mathscr {M}_{\mathcal {H}_{i}}^{+}+i \mathscr {A}_{\mathcal {H}_{i}}^{+}, \mathscr {N}_{\mathcal {H}_{i}}^{+} +i \mathscr {B}_{\mathcal {H}_{i}}^{+},\mathscr {M}_{\mathcal {H}_{i}}^{-} +i \mathscr {A}_{\mathcal {H}_{i}}^{-}, \mathscr {N}_{\mathcal {H}_{i}}^{-} +i \mathscr {B}_{\mathcal {H}_{i}}^{-}\right\rangle \right\}$. Let $\mathscr {X}=\left( \mathscr {X}_1, \mathscr {X}_2, \dots , \mathscr {X}_k\right) ^T$ represent the weight vector associated with $\mathcal {H}_i$, satisfying the conditions where $\mathscr {X}_i > 0$ and $\sum _{i=1}^{k} \mathscr {X}_i = 1$. If all elements $\mathcal {H}_{i}$ are identical to a common BCq-ROFN $\mathcal {H}$, expressed as:

$$\mathcal {H}=\left\langle \mathscr {M}_{\mathcal {H}}^{+}+i \mathscr {A}_{\mathcal {H}}^{+}, \mathscr {N}_{\mathcal {H}}^{+} +i \mathscr {B}_{\mathcal {H}}^{+},\mathscr {M}_{\mathcal {H}}^{-} +i \mathscr {A}_{\mathcal {H}}^{-}, \mathscr {N}_{\mathcal {H}}^{-} +i \mathscr {B}_{\mathcal {H}}^{-}\right\rangle$$, then

1. BCq-ROFWA$$(\mathcal {H}_1, \mathcal {H}_2, \ldots , \mathcal {H}_{k})=\mathcal {H}$$.
2. BCq-ROFWG$$(\mathcal {H}_1, \mathcal {H}_2, \ldots , \mathcal {H}_{k})=\mathcal {H}$$.

### Proof

Verifying the first case is sufficient, as the remaining case follow analogously. Given that $\mathcal {H}_i = \mathcal {H}$, $\forall i = 1,2, \dots , k$, we derive the following expression:

$$\begin{aligned} \operatorname {BCq-ROFWA}\left( \mathcal {H}_1, \mathcal {H}_2, \ldots , \mathcal {H}_{k}\right) \end{aligned}$$

$$\begin{aligned} &= \left\langle {\begin{array}{*{20}l} {(1 - \prod _{{i = 1}}^{k} (1 - ({\mathcal{M}}_{{{\mathcal{H}}_{i} }}^{ + } )^{q} )^{{{\mathcal{X}}_{i} }} )^{{\frac{1}{q}}} + i(1 - \prod _{{i = 1}}^{k} (1 - ({\mathcal{A}}_{{{\mathcal{H}}_{i} }}^{ + } )^{q} )^{{{\mathcal{X}}_{i} }} )^{{\frac{1}{q}}} ,} \hfill \\ {\prod _{{i = 1}}^{k} ({\mathcal{N}}_{{{\mathcal{H}}_{i} }}^{ + } )^{{{\mathcal{X}}_{i} }} + i\prod _{{i = 1}}^{k} ({\mathcal{B}}_{{{\mathcal{H}}_{i} }}^{ + } )^{{{\mathcal{X}}_{i} }} , - (\prod _{{i = 1}}^{k} |{\mathcal{M}}_{{{\mathcal{H}}_{i} }}^{ - } |^{{{\mathcal{X}}_{i} }} ) + i( - (\prod _{{i = 1}}^{k} |{\mathcal{A}}_{{{\mathcal{H}}_{i} }}^{ - } |^{{{\mathcal{X}}_{i} }} )),} \hfill \\ { - (1 - \prod _{{i = 1}}^{k} (1 - |{\mathcal{N}}_{{{\mathcal{H}}_{i} }}^{ - } |^{q} )^{{{\mathcal{X}}_{i} }} )^{{\frac{1}{q}}} + i( - (1 - \prod _{{i = 1}}^{k} (1 - |{\mathcal{B}}_{{{\mathcal{H}}_{i} }}^{ - } |^{q} )^{{{\mathcal{X}}_{i} }} )^{{\frac{1}{q}}} )} \hfill \\ \end{array} } \right\rangle \\ & = \left\langle {\begin{array}{*{20}l} {(1 - \prod _{{i = 1}}^{k} (1 - ({\mathcal{M}}_{{\mathcal{H}}}^{ + } )^{q} )^{{{\mathcal{X}}_{i} }} )^{{\frac{1}{q}}} + i(1 - \prod _{{i = 1}}^{k} (1 - ({\mathcal{A}}_{{\mathcal{H}}}^{ + } )^{q} )^{{{\mathcal{X}}_{i} }} )^{{\frac{1}{q}}} ,} \hfill \\ {\prod _{{i = 1}}^{k} ({\mathcal{N}}_{{\mathcal{H}}}^{ + } )^{{{\mathcal{X}}_{i} }} + i\prod _{{i = 1}}^{k} ({\mathcal{B}}_{{\mathcal{H}}}^{ + } )^{{{\mathcal{X}}_{i} }} , - (\prod _{{i = 1}}^{k} |{\mathcal{M}}_{{\mathcal{H}}}^{ - } |^{{{\mathcal{X}}_{i} }} ) + i( - (\prod _{{i = 1}}^{k} |{\mathcal{A}}_{{\mathcal{H}}}^{ - } |^{{{\mathcal{X}}_{i} }} )),} \hfill \\ { - (1 - \prod _{{i = 1}}^{k} (1 - |{\mathcal{N}}_{{\mathcal{H}}}^{ - } |^{q} )^{{{\mathcal{X}}_{i} }} )^{{\frac{1}{q}}} + i( - (1 - \prod _{{i = 1}}^{k} (1 - |{\mathcal{B}}_{{\mathcal{H}}}^{ - } |^{q} )^{{{\mathcal{X}}_{i} }} )^{{\frac{1}{q}}} )} \hfill \\ \end{array} } \right\rangle\\ & = \left\langle {\begin{array}{*{20}l} {(1 - (1 - ({\mathcal{M}}_{{\mathcal{H}}}^{ + } )^{q} )^{{\sum\limits_{{i = 1}}^{k} {{\mathcal{X}}_{i} } }} )^{{\frac{1}{q}}} + i(1 - (1 - ({\mathcal{A}}_{{\mathcal{H}}}^{ + } )^{q} )^{{\sum\limits_{{i = 1}}^{k} {{\mathcal{X}}_{i} } }} )^{{\frac{1}{q}}} ,} \hfill \\ {({\mathcal{N}}_{{\mathcal{H}}}^{ + } )^{{\sum\limits_{{i = 1}}^{k} {{\mathcal{X}}_{i} } }} + i({\mathcal{B}}_{{\mathcal{H}}}^{ + } )^{{\sum\limits_{{i = 1}}^{k} {{\mathcal{X}}_{i} } }} , - (|{\mathcal{M}}_{{\mathcal{H}}}^{ - } |^{{\sum\limits_{{i = 1}}^{k} {{\mathcal{X}}_{i} } }} ) + i( - (|{\mathcal{A}}_{{\mathcal{H}}}^{ - } |^{{\sum\limits_{{i = 1}}^{k} {{\mathcal{X}}_{i} } }} )),} \hfill \\ { - (1 - (1 - |{\mathcal{N}}_{{\mathcal{H}}}^{ - } |^{q} )^{{\sum\limits_{{i = 1}}^{k} {{\mathcal{X}}_{i} } }} )^{{\frac{1}{q}}} + i( - (1 - (1 - |{\mathcal{B}}_{{\mathcal{H}}}^{ - } |^{q} )^{{\sum\limits_{{i = 1}}^{k} {{\mathcal{X}}_{i} } }} )^{{\frac{1}{q}}} )} \hfill \\ \end{array} } \right\rangle\\ & = \left\langle {\begin{array}{*{20}l} {(1 - (1 - ({\mathcal{M}}_{{\mathcal{H}}}^{ + } )^{q} ))^{{\frac{1}{q}}} + i(1 - (1 - ({\mathcal{A}}_{{\mathcal{H}}}^{ + } )^{q} ))^{{\frac{1}{q}}} ,({\mathcal{N}}_{{\mathcal{H}}}^{ + } ) + i({\mathcal{B}}_{{\mathcal{H}}}^{ + } ),} \hfill \\ { - (|{\mathcal{M}}_{{\mathcal{H}}}^{ - } |) + i( - (|{\mathcal{A}}_{{\mathcal{H}}}^{ - } |)), - (1 - (1 - |{\mathcal{N}}_{{\mathcal{H}}}^{ - } |^{q} ))^{{\frac{1}{q}}} + i( - (1 - (1 - |{\mathcal{B}}_{{\mathcal{H}}}^{ - } |^{q} ))^{{\frac{1}{q}}} )} \hfill \\ \end{array} } \right\rangle \end{aligned}$$

$$=\left\langle \begin{array}{c}\mathscr {M}_{\mathcal {H}}^{+}+i \mathscr {A}_{\mathcal {H}}^{+}, \mathscr {N}_{\mathcal {H}}^{+} +i \mathscr {B}_{\mathcal {H}}^{+},\mathscr {M}_{\mathcal {H}}^{-} +i \mathscr {A}_{\mathcal {H}}^{-}, \mathscr {N}_{\mathcal {H}}^{-} +i \mathscr {B}_{\mathcal {H}}^{-}\end{array}\right\rangle ,$$

where $$-|\mathscr {M}_{\mathcal {H}}^{-}|=\mathscr {M}_{\mathcal {H}}^{-}$$, $$-|\mathscr {N}_{\mathcal {H}}^{-}|=\mathscr {N}_{\mathcal {H}}^{-}$$, $$-|\mathscr {A}_{\mathcal {H}}^{-}|=\mathscr {A}_{\mathcal {H}}^{-}$$ and $$-|\mathscr {B}_{\mathcal {H}}^{-}|=\mathscr {B}_{\mathcal {H}}^{-}$$. $$\square$$

### Theorem 4.6

(Boundedness) Consider a set of BCq-ROFNs defined as:

$\mathcal {H}_{i}=\left\{ \left\langle \mathscr {M}_{\mathcal {H}_{i}}^{+}+i \mathscr {A}_{\mathcal {H}_{i}}^{+}, \mathscr {N}_{\mathcal {H}_{i}}^{+} +i \mathscr {B}_{\mathcal {H}_{i}}^{+},\mathscr {M}_{\mathcal {H}_{i}}^{-} +i \mathscr {A}_{\mathcal {H}_{i}}^{-}, \mathscr {N}_{\mathcal {H}_{i}}^{-} +i \mathscr {B}_{\mathcal {H}_{i}}^{-}\right\rangle \right\}$, $\forall i = 1,2, \dots , k.$ Let $\mathscr {X}=\left( \mathscr {X}_1, \mathscr {X}_2, \ldots , \mathscr {X}_k\right) ^T$ be the vector of weights associated with each $\mathcal {H}_i$, where each weight satisfies $\mathscr {X}_i > 0$ and the sum of all weights is normalized as $\sum _{i=1}^{k} \mathscr {X}_i=1$. Now, suppose two BCq-ROFNs, denoted as $\overline{\mathcal {H}}$ and $\underline{\mathcal {H}}$, are defined such that:

$$\overline{\mathcal {H}}= \left\langle {\mathscr {M}_{\overline{\mathcal {H}}}^{+}}^{*}+i {\mathscr {A}_{\overline{\mathcal {H}}}^{+}}^{*}, {\mathscr {N}_{\overline{\mathcal {H}}}^{+}}^{\cdot } +i {\mathscr {B}_{\overline{\mathcal {H}}}^{+}}^{\cdot },{\mathscr {M}_{\overline{\mathcal {H}}}^{-}}^{\cdot } +i {\mathscr {A}_{\overline{\mathcal {H}}}^{-}}^{\cdot }, {\mathscr {N}_{\overline{\mathcal {H}}}^{-}}^{*} +i {\mathscr {B}_{\overline{\mathcal {H}}}^{-}}^{*}\right\rangle,$$

which may further be rewritten in terms of the maximum and minimum values of the given BCq-ROFNs as:

$$\overline{\mathcal {H}}= \left\langle \max (\mathscr {M}_{\overline{\mathcal {H}_{i}}}^{+}) +i \max (\mathscr {A}_{\overline{\mathcal {H}_{i}}}^{+}), \min (\mathscr {N}_{\overline{\mathcal {H}_{i}}}^{+}) +i \min (\mathscr {B}_{\overline{\mathcal {H}_{i}}}^{+}), \min (\mathscr {M}_{\overline{\mathcal {H}_{i}}}^{-}) +i \min (\mathscr {A}_{\overline{\mathcal {H}_{i}}}^{-}), \max (\mathscr {N}_{\overline{\mathcal {H}_{i}}}^{-}) +i \max (\mathscr {B}_{\overline{\mathcal {H}_{i}}}^{-})\right\rangle$$, where $$1 \le i \le k$$.

Similarly, another BCq-ROFN $\underline{\mathcal {H}}$ is defined as:

$$\underline{\mathcal {H}}= \left\langle {\mathscr {M}_{\underline{\mathcal {H}}}^{+}}^{\cdot }+i {\mathscr {A}_{\underline{\mathcal {H}}}^{+}}^{\cdot }, {\mathscr {N}_{\underline{\mathcal {H}}}^{+}}^{*} +i {\mathscr {B}_{\underline{\mathcal {H}}}^{+}}^{*},{\mathscr {M}_{\underline{\mathcal {H}}}^{-}}^{*} +i {\mathscr {A}_{\underline{\mathcal {H}}}^{-}}^{*}, {\mathscr {N}_{\underline{\mathcal {H}}}^{-}}^{\cdot } +i {\mathscr {B}_{\underline{\mathcal {H}}}^{-}}^{\cdot }\right\rangle,$$

which can also be rewritten in terms of the minimum and maximum values as:

$$\underline{\mathcal {H}}= \left\langle \min (\mathscr {M}_{\underline{\mathcal {H}}_{i}}^{+}) +i \min (\mathscr {A}_{\underline{\mathcal {H}}_{i}}^{+}), \max (\mathscr {N}_{\underline{\mathcal {H}}_{i}}^{+}) +i \max (\mathscr {B}_{\underline{\mathcal {H}}_{i}}^{+}), \max (\mathscr {M}_{\underline{\mathcal {H}}_{i}}^{-}) +i \max (\mathscr {A}_{\underline{\mathcal {H}}_{i}}^{-}), \min (\mathscr {N}_{\underline{\mathcal {H}}_{i}}^{-}) +i \min (\mathscr {B}_{\underline{\mathcal {H}}_{i}}^{-})\right\rangle$$, where $$1 \le i \le k$$. Then,

1. $$\underline{\mathcal {H}} \le \operatorname {BCq-ROFWA}(\mathcal {H}_{1},\mathcal {H}_{2},\ldots ,\mathcal {H}_{k})\le \overline{\mathcal {H}}$$.
2. $$\underline{\mathcal {H}} \le \operatorname {BCq-ROFWG}(\mathcal {H}_{1},\mathcal {H}_{2},\ldots ,\mathcal {H}_{k})\le \overline{\mathcal {H}}$$.

### Proof

We will demonstrate the first result, noting that the others follow by analogous reasoning. To establish the first part, it is crucial to confirm that:

$$\begin{aligned} &{\mathscr {M}_{\underline{\mathcal {H}}}^{+}}^{\cdot }\le (1-{\prod }_{i=1}^k(1-(\mathscr {M}_{\mathcal {H}_i}^{+})^{q})^{\mathscr {X}_i})^{\frac{1}{q}} \le {\mathscr {M}_{\underline{\mathcal {H}}}^{+}}^{*}, \\& {\mathscr {A}_{\underline{\mathcal {H}}}^{+}}^{\cdot }\le (1-{\prod }_{i=1}^k(1-(\mathscr {A}_{\mathcal {H}_i}^{+})^{q})^{\mathscr {X}_i})^{\frac{1}{q}} \le {\mathscr {A}_{\underline{\mathcal {H}}}^{+}}^{*}, \\&{\mathscr {N}_{\underline{\mathcal {H}}}^{+}}^{*}\ge {\prod }_{i=1}^k(\mathscr {N}_{\mathcal {H}_i}^{+})^{\mathscr {X}_i} \ge {\mathscr {N}_{\underline{\mathcal {H}}}^{+}}^{\cdot }, \\&{\mathscr {B}_{\underline{\mathcal {H}}}^{+}}^{*}\ge {\prod }_{i=1}^k(\mathscr {B}_{\mathcal {H}_i}^{+})^{\mathscr {X}_i} \ge {\mathscr {B}_{\underline{\mathcal {H}}}^{+}}^{\cdot }, \\& {\mathscr {M}_{\underline{\mathcal {H}}}^{-}}^{*} \ge -({\prod }_{i=1}^k|\mathscr {M}_{\mathcal {H}_i}^{-}|^{\mathscr {X}_i}) \ge {\mathscr {M}_{\underline{\mathcal {H}}}^{-}}^{\cdot }, \\&{\mathscr {A}_{\underline{\mathcal {H}}}^{-}}^{*}\ge -({\prod }_{i=1}^k|\mathscr {A}_{\mathcal {H}_i}^{-}|^{\mathscr {X}_i}) \ge {\mathscr {A}_{\underline{\mathcal {H}}}^{-}}^{\cdot }, \\&{\mathscr {N}_{\underline{\mathcal {H}}}^{-}}^{\cdot }\le -(1-{\prod }_{i=1}^k(1-|\mathscr {N}_{\mathcal {H}_i}^{-}|^{q})^{\mathscr {X}_i})^{\frac{1}{q}} \le {\mathscr {N}_{\underline{\mathcal {H}}}^{-}}^{*}, \\& {\mathrm{and}}\\&{\mathscr {B}_{\underline{\mathcal {H}}}^{-}}^{\cdot }\le -(1-{\prod }_{i=1}^k(1-|\mathscr {B}_{\mathcal {H}_i}^{-}|^{q})^{\mathscr {X}_i})^{\frac{1}{q}} \le {\mathscr {B}_{\underline{\mathcal {H}}}^{-}}^{*}\end{aligned}$$

Given the following inequalities:

$${\mathscr {M}_{\underline{\mathcal {H}}}^{+}}^{\cdot } \le \mathscr {M}_{{\mathcal {H}_{i}}}^{+} \le {\mathscr {M}_{\overline{\mathcal {H}}}^{+}}^{*}, \quad {\mathscr {M}_{\underline{\mathcal {H}}}^{-}}^{\cdot } \le \mathscr {M}_{{\mathcal {H}_{i}}}^{-} \le {\mathscr {M}_{\overline{\mathcal {H}}}^{-}}^{*},$$

$${\mathscr {N}_{\underline{\mathcal {H}}}^{+}}^{\cdot } \le \mathscr {N}_{{\mathcal {H}_{i}}}^{+} \le {\mathscr {N}_{\overline{\mathcal {H}}}^{+}}^{*}, \quad {\mathscr {N}_{\underline{\mathcal {H}}}^{-}}^{\cdot } \le \mathscr {N}_{{\mathcal {H}_{i}}}^{-} \le {\mathscr {N}_{\overline{\mathcal {H}}}^{-}}^{*},$$

we can similarly assert the following for the additional terms:

$${\mathscr {A}_{\underline{\mathcal {H}}}^{+}}^{\cdot } \le \mathscr {A}_{\mathcal {H}_{i}}^{+} \le {\mathscr {A}_{\overline{\mathcal {H}}}^{+}}^{*}, \quad {\mathscr {A}_{\underline{\mathcal {H}}}^{-}}^{\cdot } \le \mathscr {A}_{{\mathcal {H}_{i}}}^{-} \le {\mathscr {A}_{\overline{\mathcal {H}}}^{-}}^{*},$$

$${\mathscr {B}_{\underline{\mathcal {H}}}^{+}}^{\cdot } \le \mathscr {B}_{{\mathcal {H}_{i}}}^{+} \le {\mathscr {B}_{\overline{\mathcal {H}}}^{+}}^{*}, \quad {\mathscr {B}_{\underline{\mathcal {H}}}^{-}}^{\cdot } \le \mathscr {B}_{{\mathcal {H}_{i}}}^{-} \le {\mathscr {B}_{\overline{\mathcal {H}}}^{-}}^{*}.$$

From these inequalities, we can conclude that:

$$\begin{aligned} \mathscr {M}_{\mathcal {H}}^{+^{\cdot }} &=(1-(1-{(\mathscr {M}_{\mathcal {H}}^{+^{\cdot }})^{q})^{\sum _{i=1}^k}}{\,}^{\mathscr {X}_i})^{\frac{1}{q}}\\ & =(1-{\prod }_{i=1}^k(1-(\mathscr {M}_{\mathcal {H}}^{+^{\cdot }})^{q})^{\mathscr {X}_i})^{\frac{1}{q}}\\ &\le (1-{\prod }_{i=1}^k(1-(\mathscr {M}_{\mathcal {H}_i}^{+})^{q})^{\mathscr {X}_i})^{\frac{1}{q}}\\ &\le (1-{\prod }_{i=1}^k(1-(\mathscr {M}_{\mathcal {H}}^{+^*})^{q})^{\mathscr {X}_i})^{\frac{1}{q}}\\ & =(1-(1-(\mathscr {M}_{\mathcal {H}}^{+^*})^{q})^{\sum _{i=1}^k {\mathscr {X}_i}})^{\frac{1}{q}}=\mathscr {M}_{\mathcal {H}}^{+^*}, \end{aligned}$$

$$\begin{aligned} \mathscr {N}_{\mathcal {H}}^{+^{\cdot }}=&(\mathscr {N}_{\mathcal {H}}^{+^{\cdot }})^{\sum _{i=1}^k \mathscr {X}_i}\\ = & {\prod }_{i=1}^k(\mathscr {N}_{\mathcal {H}}^{+^{\cdot }})^{\mathscr {X}_i}\\ \le & {\prod }_{i=1}^k(\mathscr {N}_{\mathcal {H}_i}^{+})^{\mathscr {X}_i}\\ \le & {\prod }_{i=1}^k(\mathscr {N}_{\mathcal {H}}^{+^*})^{\mathscr {X}_i}= (\mathscr {N}_{\mathcal {H}}^{+^*})^{\sum _{i=1}^k \mathscr {X}_i}=\mathscr {N}_{\mathcal {H}}^{+^*},\\ \mathscr {M}_{\mathcal {H}}^{-^{\cdot }}=& -(|\mathscr {M}_{\mathcal {H}}^{-^{\cdot }}|^{\sum _{i=1}^k \mathscr {X}_i})\\ =&-({\prod }_{i=1}^k(|\mathscr {M}_{\mathcal {H}}^{-^{\cdot }}|)^{\mathscr {X}_i})\\ \le& -({\prod }_{i=1}^k(|\mathscr {M}_{\mathcal {H}_i}^{-}|)^{\mathscr {X}_i})\\ \le& -({\prod }_{i=1}^k(|\mathscr {M}_{\mathcal {H}}^{-^*}|)^{\mathscr {X}_i})= -(|\mathscr {M}_{\mathcal {H}}^{-^*}|^{\sum _{i=1}^k \mathscr {X}_i})=-|\mathscr {M}_{\mathcal {H}}^{-^*}|=\mathscr {M}_{\mathcal {H}}^{-^*}, \end{aligned}$$

and

$$\begin{aligned} \mathscr {N}_{\mathcal {H}}^{-^{\cdot }}&= -(1-(1-|\mathscr {N}_{\mathcal {H}}^{-^{\cdot }}|^{q})^{\sum _{i=1}^k}{\,}^{\mathscr {X}_i})^{\frac{1}{q}}\\ & =-(1-{\prod }_{i=1}^k(1- |\mathscr {N}_{\mathcal {H}}^{-^{\cdot }}|^{q})^{\mathscr {X}_i})^{\frac{1}{q}}\\ & \le -(1-{\prod }_{i=1}^k(1-|\mathscr {N}_{\mathcal {H}_i}^{-}|^{q})^{\mathscr {X}_i})^{\frac{1}{q}}\\ & \le -(1-{\prod }_{i=1}^k(1-|\mathscr {N}_{\mathcal {H}}^{-^*}|^{q})^{\mathscr {X}_i})^{\frac{1}{q}}=-(1-(1-(\mathscr {N}_{\mathcal {H}}^{-^*})^{q})^{\sum _{i=1}^k {\mathscr {X}_i}})^{\frac{1}{q}}\\ & =-|\mathscr {N}_{\mathcal {H}}^{-^*}|=\mathscr {N}_{\mathcal {H}}^{-^*}. \end{aligned}$$

Following the same approach, we can also show that:

$$\begin{aligned}&{\mathscr {A}_{\underline{\mathcal {H}}}^{+}}^{\cdot }\le (1-{\prod }_{i=1}^k(1-(\mathscr {A}_{\mathcal {H}_i}^{+})^{q})^{\mathscr {X}_i})^{\frac{1}{q}} \le {\mathscr {A}_{\underline{\mathcal {H}}}^{+}}^{*}, \\&{\mathscr {B}_{\underline{\mathcal {H}}}^{+}}^{*}\ge {\prod }_{i=1}^k(\mathscr {B}_{\mathcal {H}_i}^{+})^{\mathscr {X}_i} \ge {\mathscr {B}_{\underline{\mathcal {H}}}^{+}}^{\cdot },\\& {\mathscr {A}_{\underline{\mathcal {H}}}^{-}}^{*}\ge -({\prod }_{i=1}^k|\mathscr {A}_{\mathcal {H}_i}^{-}|^{\mathscr {X}_i}) \ge {\mathscr {A}_{\underline{\mathcal {H}}}^{-}}^{\cdot },\\& {\mathrm{and}} \\& {\mathscr {B}_{\underline{\mathcal {H}}}^{-}}^{\cdot }\le -(1-{\prod }_{i=1}^k(1-|\mathscr {B}_{\mathcal {H}_i}^{-}|^{q})^{\mathscr {X}_i})^{\frac{1}{q}} \le {\mathscr {B}_{\underline{\mathcal {H}}}^{-}}^{*} \end{aligned}$$

$$\square$$

### Theorem 4.7

(Monotonicity) Let

$$\mathcal {H}_{i}=\left\{ \langle \mathscr {M}_{\mathcal {H}_{i}}^{+}+i \mathscr {A}_{\mathcal {H}_{i}}^{+}, \mathscr {N}_{\mathcal {H}_{i}}^{+} +i \mathscr {B}_{\mathcal {H}_{i}}^{+},\mathscr {M}_{\mathcal {H}_{i}}^{-} +i \mathscr {A}_{\mathcal {H}_{i}}^{-}, \mathscr {N}_{\mathcal {H}_{i}}^{-} +i \mathscr {B}_{\mathcal {H}_{i}}^{-}\rangle \right\}$$ and

$\tilde{\mathcal {H}_{i}}=\left\{ \langle \mathscr {M}_{\tilde{\mathcal {H}_{i}}}^{+}+i \mathscr {A}_{\tilde{\mathcal {H}_{i}}}^{+}, \mathscr {N}_{\tilde{\mathcal {H}_{i}}}^{+} +i \mathscr {B}_{\tilde{\mathcal {H}_{i}}}^{+},\mathscr {M}_{\tilde{\mathcal {H}_{i}}}^{-} +i \mathscr {A}_{\tilde{\mathcal {H}_{i}}}^{-}, \mathscr {N}_{\tilde{\mathcal {H}_{i}}}^{-} +i \mathscr {B}_{\tilde{\mathcal {H}_{i}}}^{-}\rangle \right\}$ represent two sets of BCq-ROFNs, indexed by $i = 1, 2, \dots , k$. If $\mathcal {H}_{i} \subset \tilde{\mathcal {H}_{i}}$ for every *i*, then:

1. $$\operatorname {BCq-ROFWA}(\mathcal {H}_{1},\mathcal {H}_{2},\ldots ,\mathcal {H}_{k})\le \operatorname {BCq-ROFWA}(\tilde{\mathcal {H}_{1}},\tilde{\mathcal {H}_{2}},\ldots ,\tilde{\mathcal {H}_{k}})$$.
2. $$\operatorname {BCq-ROFWG}(\mathcal {H}_{1},\mathcal {H}_{2},\ldots ,\mathcal {H}_{k})\le \operatorname {BCq-ROFWG}(\tilde{\mathcal {H}_{1}},\tilde{\mathcal {H}_{2}},\ldots ,\tilde{\mathcal {H}_{k}})$$.

### Proof

It is enough to prove the first part, as the second follows in a similar manner. The following relations hold for each $i$:

$$\mathscr {M}_{\mathcal {H}_i}^{+} \le \mathscr {M}_{\tilde{\mathcal {H}_{i}}}^{+}, \mathscr {M}_{\mathcal {H}_i}^{-} \ge \mathscr {M}_{\tilde{\mathcal {H}_{i}}}^{-}, \mathscr {N}_{\mathcal {H}_i}^{+} \ge \mathscr {N}_{\tilde{\mathcal {H}_{i}}}^{+}, \mathscr {N}_{\mathcal {H}_i}^{-} \le \mathscr {N}_{\tilde{\mathcal {H}_{i}}}^{-}$$,

$$\mathscr {A}_{\mathcal {H}_i}^{+} \le \mathscr {A}_{\tilde{\mathcal {H}_{i}}}^{+}, \mathscr {A}_{\mathcal {H}_i}^{-} \ge \mathscr {A}_{\tilde{\mathcal {H}_{i}}}^{-}, \mathscr {B}_{\mathcal {H}_i}^{+} \ge \mathscr {B}_{\tilde{\mathcal {H}_{i}}}^{+},$$ and $$\mathscr {B}_{\mathcal {H}_i}^{-} \le \mathscr {B}_{\tilde{\mathcal {H}_{i}}}^{-}$$.

From this, we can obtain the following inequalities:

$$\begin{aligned} &( 1-{\prod }_{i=1}^k(1- (\mathscr {M}_{\mathcal {H}_i}^{+})^{q})^{\mathscr {X}_i})^{\frac{1}{q}} \le ( 1-{\prod }_{i=1}^k(1- (\mathscr {M}_{\tilde{\mathcal {H}_{i}}}^{+})^{q})^{\mathscr {X}_i})^{\frac{1}{q}},\\ &( 1-{\prod }_{i=1}^k(1- (\mathscr {A}_{\mathcal {H}_i}^{+})^{q})^{\mathscr {X}_i})^{\frac{1}{q}} \le ( 1-{\prod }_{i=1}^k(1- (\mathscr {A}_{\tilde{\mathcal {H}_{i}}}^{+})^{q})^{\mathscr {X}_i})^{\frac{1}{q}},\\& {\prod }_{i=1}^k(\mathscr {N}_{\mathcal {H}_i}^{+})^{\mathscr {X}_i} \ge {\prod }_{i=1}^k(\mathscr {N}_{\tilde{\mathcal {H}_i}}^{+})^{\mathscr {X}_i},\\& {\prod }_{i=1}^k(\mathscr {B}_{\mathcal {H}_i}^{+})^{\mathscr {X}_i} \ge {\prod }_{i=1}^k(\mathscr {B}_{\tilde{\mathcal {H}_i}}^{+})^{\mathscr {X}_i}, \end{aligned}$$

$$\begin{aligned} &-({\prod }_{i=1}^k|\mathscr {M}_{\mathcal {H}_i}^{-}|^{\mathscr {X}_i})\ge -({\prod }_{i=1}^k|\mathscr {M}_{\tilde{\mathcal {H}_i}}^{-}|^{\mathscr {X}_i}),\\ & -({\prod }_{i=1}^k|\mathscr {A}_{\mathcal {H}_i}^{-}|^{\mathscr {X}_i})\ge -({\prod }_{i=1}^k|\mathscr {A}_{\tilde{\mathcal {H}_i}}^{-}|^{\mathscr {X}_i}),\\& -(1-{\prod }_{i=1}^k(1-|\mathscr {N}_{\mathcal {H}_i}^{-}|^{q})^{\mathscr {X}_i})^{\frac{1}{q}}\le -(1-{\prod }_{i=1}^k(1-|\mathscr {N}_{\tilde{\mathcal {H}_i}}^{-}|^{q})^{\mathscr {X}_i})^{\frac{1}{q}}, \end{aligned}$$

and

$$\begin{aligned} -(1-{\prod }_{i=1}^k(1-|\mathscr {B}_{\mathcal {H}_i}^{-}|^{q})^{\mathscr {X}_i})^{\frac{1}{q}}\le -(1-{\prod }_{i=1}^k(1-|\mathscr {B}_{\tilde{\mathcal {H}_i}}^{-}|^{q})^{\mathscr {X}_i})^{\frac{1}{q}}. \end{aligned}$$

Therefore, it follows that:

$$\begin{aligned} \operatorname {BCq-ROFWA}(\mathcal {H}_{1},\mathcal {H}_{2},\ldots ,\mathcal {H}_{k}) \end{aligned}$$

$$= \left\langle {\begin{array}{*{20}l} {(1 - \prod _{{i = 1}}^{k} (1 - ({\mathcal{M}}_{{{\mathcal{H}}_{i} }}^{ + } )^{q} )^{{{\mathcal{X}}_{i} }} )^{{\frac{1}{q}}} + i(1 - \prod _{{i = 1}}^{k} (1 - ({\mathcal{A}}_{{{\mathcal{H}}_{i} }}^{ + } )^{q} )^{{{\mathcal{X}}_{i} }} )^{{\frac{1}{q}}} ,} \hfill \\ {\prod _{{i = 1}}^{k} ({\mathcal{N}}_{{{\mathcal{H}}_{i} }}^{ + } )^{{{\mathcal{X}}_{i} }} + i\prod _{{i = 1}}^{k} ({\mathcal{B}}_{{{\mathcal{H}}_{i} }}^{ + } )^{{{\mathcal{X}}_{i} }} , - (\prod _{{i = 1}}^{k} |{\mathcal{M}}_{{{\mathcal{H}}_{i} }}^{ - } |^{{{\mathcal{X}}_{i} }} ) + i( - (\prod _{{i = 1}}^{k} |{\mathcal{A}}_{{{\mathcal{H}}_{i} }}^{ - } |^{{{\mathcal{X}}_{i} }} )),} \hfill \\ { - (1 - \prod _{{i = 1}}^{k} (1 - |{\mathcal{N}}_{{{\mathcal{H}}_{i} }}^{ - } |^{q} )^{{{\mathcal{X}}_{i} }} )^{{\frac{1}{q}}} + i( - (1 - \prod _{{i = 1}}^{k} (1 - |{\mathcal{B}}_{{{\mathcal{H}}_{i} }}^{ - } |^{q} )^{{{\mathcal{X}}_{i} }} )^{{\frac{1}{q}}} )} \hfill \\ \end{array} } \right\rangle$$

$$\le \left\langle {\begin{array}{*{20}l} {(1 - \prod _{{i = 1}}^{k} (1 - ({\mathcal{M}}_{{\widetilde{{{\mathcal{H}}_{i} }}}}^{ + } )^{q} )^{{{\mathcal{X}}_{i} }} )^{{\frac{1}{q}}} + i(1 - \prod _{{i = 1}}^{k} (1 - ({\mathcal{A}}_{{\widetilde{{{\mathcal{H}}_{i} }}}}^{ + } )^{q} )^{{{\mathcal{X}}_{i} }} )^{{\frac{1}{q}}} ,} \hfill \\ {\prod _{{i = 1}}^{k} ({\mathcal{N}}_{{\widetilde{{{\mathcal{H}}_{i} }}}}^{ + } )^{{{\mathcal{X}}_{i} }} + i\prod _{{i = 1}}^{k} ({\mathcal{B}}_{{\widetilde{{{\mathcal{H}}_{i} }}}}^{ + } )^{{{\mathcal{X}}_{i} }} , - (\prod _{{i = 1}}^{k} |{\mathcal{M}}_{{\widetilde{{{\mathcal{H}}_{i} }}}}^{ - } |^{{{\mathcal{X}}_{i} }} ) + i( - (\prod _{{i = 1}}^{k} |{\mathcal{A}}_{{\widetilde{{{\mathcal{H}}_{i} }}}}^{ - } |^{{{\mathcal{X}}_{i} }} )),} \hfill \\ { - (1 - \prod _{{i = 1}}^{k} (1 - |{\mathcal{N}}_{{\widetilde{{{\mathcal{H}}_{i} }}}}^{ - } |^{q} )^{{{\mathcal{X}}_{i} }} )^{{\frac{1}{q}}} + i( - (1 - \prod _{{i = 1}}^{k} (1 - |{\mathcal{B}}_{{\widetilde{{{\mathcal{H}}_{i} }}}}^{ - } |^{q} )^{{{\mathcal{X}}_{i} }} )^{{\frac{1}{q}}} )} \hfill \\ \end{array} } \right\rangle$$

$$\begin{aligned} =\operatorname {BCq-ROFWA}(\tilde{\mathcal {H}_{1}},\tilde{\mathcal {H}_{2}},\ldots ,\tilde{\mathcal {H}_{k}}). \end{aligned}$$

$$\square$$

### Theorem 4.8

Let $\mathcal {H}_{i} = \left\{ \left\langle \mathscr {M}_{\mathcal {H}_{i}}^{+} + i \mathscr {A}_{\mathcal {H}_{i}}^{+}, \mathscr {N}_{\mathcal {H}_{i}}^{+} + i \mathscr {B}_{\mathcal {H}_{i}}^{+}, \mathscr {M}_{\mathcal {H}_{i}}^{-} + i \mathscr {A}_{\mathcal {H}_{i}}^{-}, \mathscr {N}_{\mathcal {H}_{i}}^{-} + i \mathscr {B}_{\mathcal {H}_{i}}^{-} \right\rangle \right\}$, for $i = 1,2, \dots , k$, represent a collection of BCq-ROFNs. Additionally, consider

$\mathscr {X} = \left( \mathscr {X}_1, \mathscr {X}_2, \dots , \mathscr {X}_k\right) ^T$ is a weight vector associated with $\mathcal {H}_i$, where each weight satisfies $\mathscr {X}_i > 0$ and the sum of all weights is normalized as $\sum _{i=1}^{k} \mathscr {X}_i = 1$. Then,

1. $$\operatorname {BCq-ROFWA}(\mathcal {H}_{1}^{c},\mathcal {H}_{2}^{c},\ldots ,\mathcal {H}_{k}^{c})=(\operatorname {BCq-ROFWG}(\mathcal {H}_{1},\mathcal {H}_{2},\ldots ,\mathcal {H}_{k}))^{c}$$.
2. $$\operatorname {BCq-ROFWG}(\mathcal {H}_{1}^{c},\mathcal {H}_{2}^{c},\ldots ,\mathcal {H}_{k}^{c})=(\operatorname {BCq-ROFWA}(\mathcal {H}_{1},\mathcal {H}_{2},\ldots ,\mathcal {H}_{k}))^{c}$$.

### Proof

By Theorem 3.13, we derive the following results:

1. $$\begin{aligned}\operatorname {BCq-ROFWA}(\mathcal {H}_{1}^{c},\mathcal {H}_{2}^{c},\ldots ,\mathcal {H}_{k}^{c})=&\mathscr {X}_{1}\mathcal {H}_{1}^{c}\oplus \mathscr {X}_{2}\mathcal {H}_{2}^{c}\oplus \ldots \oplus \mathscr {X}_{k}\mathcal {H}_{k}^{c} \\=&(\mathcal {H}_{1}^{\mathscr {X}_{1}})^{c}\oplus (\mathcal {H}_{2}^{\mathscr {X}_{2}})^{c}\oplus \ldots \oplus (\mathcal {H}_{k}^{\mathscr {X}_{k}})^{c}\\ =&((\mathcal {H}_{1}^{\mathscr {X}_{1}})\otimes (\mathcal {H}_{2}^{\mathscr {X}_{2}})\otimes \ldots \otimes (\mathcal {H}_{k}^{\mathscr {X}_{k}}))^{c}\\ =&(\operatorname {BCq-ROFWG}(\mathcal {H}_{1},\mathcal {H}_{2},\ldots ,\mathcal {H}_{k}))^{c}.\end{aligned}$$
2. $$\begin{aligned} \operatorname {BCq-ROFWG}(\mathcal {H}_{1}^{c},\mathcal {H}_{2}^{c},\ldots ,\mathcal {H}_{k}^{c})=&(\mathcal {H}_{1}^{c})^{\mathscr {X}_{1}}\otimes (\mathcal {H}_{2}^{c})^{\mathscr {X}_{2}}\otimes \ldots \otimes (\mathcal {H}_{k}^{c})^{\mathscr {X}_{k}}\\=&(\mathscr {X}_{1}\mathcal {H}_{1})^{c}\otimes (\mathscr {X}_{2}\mathcal {H}_{2})^{c}\otimes \ldots \otimes (\mathscr {X}_{k}\mathcal {H}_{k})^{c}\\ =&(\mathscr {X}_{1}\mathcal {H}_{1}\oplus \mathscr {X}_{2}\mathcal {H}_{2}\oplus \ldots \oplus \mathscr {X}_{k}\mathcal {H}_{k})^{c}\\ =&(\operatorname {BCq-ROFWA}(\mathcal {H}_{1},\mathcal {H}_{2},\ldots ,\mathcal {H}_{k}))^{c}. \end{aligned}$$

$$\square$$

We introduce two essential functions that are pivotal for ranking BCq-ROFNs.

### Definition 4.9

Let $\mathcal {H}= \langle \mathscr {M}_{\mathcal {H}}^{+}+i \mathscr {A}_{\mathcal {H}}^{+}, \mathscr {N}_{\mathcal {H}}^{+} +i \mathscr {B}_{\mathcal {H}}^{+},\mathscr {M}_{\mathcal {H}}^{-} +i \mathscr {A}_{\mathcal {H}}^{-}, \mathscr {N}_{\mathcal {H}}^{-} +i \mathscr {B}_{\mathcal {H}}^{-}\rangle$ be an arbitrary BCq-ROFN. Then, the following functions are defined:

1. The score function of $\mathcal {H}$ is expressed as:
  12 $$\begin{aligned}{\gamma }(\mathcal {H}) =&\frac{1}{4} ( \left[ (\mathscr {M}_{\mathcal {H}}^{+})^{q} - (\mathscr {N}_{\mathcal {H}}^{+})^{q} \right] + \left[ (\mathscr {A}_{\mathcal {H}}^{+})^{q} - (\mathscr {B}_{\mathcal {H}}^{+})^{q} \right] \nonumber \\&\quad + \left[ |\mathscr {M}_{\mathcal {H}}^{-}|^{q} - |\mathscr {N}_{\mathcal {H}}^{-}|^{q} \right] + \left[ |\mathscr {A}_{\mathcal {H}}^{-}|^{q} - |\mathscr {B}_{\mathcal {H}}^{-}|^{q} \right] ) \end{aligned}$$
2. The accuracy function of $\mathcal {H}$ is expressed as:
  13 $$\begin{aligned}{\lambda }(\mathcal {H}) &= \frac{1}{4} ( \left[ (\mathscr {M}_{\mathcal {H}}^{+})^{q} + (\mathscr {N}_{\mathcal {H}}^{+})^{q} \right] + \left[ (\mathscr {A}_{\mathcal {H}}^{+})^{q} + (\mathscr {B}_{\mathcal {H}}^{+})^{q} \right] \nonumber \\ &\quad + \left[ |\mathscr {M}_{\mathcal {H}}^{-}|^{q} + |\mathscr {N}_{\mathcal {H}}^{-}|^{q} \right] + \left[ |\mathscr {A}_{\mathcal {H}}^{-}|^{q} + |\mathscr {B}_{\mathcal {H}}^{-}|^{q} \right] ) \end{aligned}$$

### Example 4.10

Consider Example 4.3. The following results are observed:

1. The score function for $\operatorname {BCq-ROFWA}(\mathcal {H}_1, \mathcal {H}_2, \mathcal {H}_3)$ is approximately:
  $$\begin{array}{c|c} \textbf{q} & \gamma \big (\operatorname {BCq-ROFWA}(\mathcal {H}_1, \mathcal {H}_2, \mathcal {H}_3)\big ) \\ \hline 1 & .2955 \\ 2 & .2721 \\ 3 & .2160 \\ 4 & .1672 \\ 5 & .1297 \\ \end{array}$$
  Additionally, the score function for $\operatorname {BCq-ROFWG}(\mathcal {H}_1, \mathcal {H}_2, \mathcal {H}_3)$ is approximately:
  $$\begin{array}{c|c} \textbf{q} & \gamma \big (\operatorname {BCq-ROFWG}(\mathcal {H}_1, \mathcal {H}_2, \mathcal {H}_3)\big ) \\ \hline 1 & .2555 \\ 2 & .2125 \\ 3 & .1595 \\ 4 & .1227 \\ 5 & .0983 \\ \end{array}$$
2. The accuracy function for $\operatorname {BCq-ROFWA}(\mathcal {H}_1, \mathcal {H}_2, \mathcal {H}_3)$ is approximately:
  $$\begin{array}{c|c} \textbf{q} & \lambda \big (\operatorname {BCq-ROFWA}(\mathcal {H}_1, \mathcal {H}_2, \mathcal {H}_3)\big ) \\ \hline 1 & .9203 \\ 2 & .5287 \\ 3 & .3438 \\ 4 & .2403 \\ 5 & .1754 \\ \end{array}$$
  Lastly, the accuracy function for $\operatorname {BCq-ROFWG}(\mathcal {H}_1, \mathcal {H}_2, \mathcal {H}_3)$ is approximately:
  $$\begin{array}{c|c} \textbf{q} & \lambda \big (\operatorname {BCq-ROFWG}(\mathcal {H}_1, \mathcal {H}_2, \mathcal {H}_3)\big ) \\ \hline 1 & .8752 \\ 2 & .4817 \\ 3 & .3032 \\ 4 & .2093 \\ 5 & .1548 \\ \end{array}$$

### Remark 4.11

For any BCq-ROFN

$\mathcal {H}= \langle \mathscr {M}_{\mathcal {H}}^{+}+i \mathscr {A}_{\mathcal {H}}^{+}, \mathscr {N}_{\mathcal {H}}^{+} +i \mathscr {B}_{\mathcal {H}}^{+},\mathscr {M}_{\mathcal {H}}^{-} +i \mathscr {A}_{\mathcal {H}}^{-}, \mathscr {N}_{\mathcal {H}}^{-} +i \mathscr {B}_{\mathcal {H}}^{-}\rangle$, the following results hold:

1. The score function satisfies: ${\gamma }(\mathcal {H}) \in [-1,1]$.
2. The accuracy function satisfies: ${\lambda }(\mathcal {H}) \in [0,1]$.

### Definition 4.12

Consider two BCq-ROFNs,

$$\mathcal {H}_{1} = \left\langle \mathscr {M}_{\mathcal {H}_{1}}^{+} + i \mathscr {A}_{\mathcal {H}_{1}}^{+}, \mathscr {N}_{\mathcal {H}_{1}}^{+} + i \mathscr {B}_{\mathcal {H}_{1}}^{+}, \mathscr {M}_{\mathcal {H}_{1}}^{-} + i \mathscr {A}_{\mathcal {H}_{1}}^{-}, \mathscr {N}_{\mathcal {H}_{1}}^{-} + i \mathscr {B}_{\mathcal {H}_{1}}^{-} \right\rangle$$

and

$$\mathcal {H}_{2} = \left\langle \mathscr {M}_{\mathcal {H}_{2}}^{+} + i \mathscr {A}_{\mathcal {H}_{2}}^{+}, \mathscr {N}_{\mathcal {H}_{2}}^{+} + i \mathscr {B}_{\mathcal {H}_{2}}^{+}, \mathscr {M}_{\mathcal {H}_{2}}^{-} + i \mathscr {A}_{\mathcal {H}_{2}}^{-}, \mathscr {N}_{\mathcal {H}_{2}}^{-} + i \mathscr {B}_{\mathcal {H}_{2}}^{-} \right\rangle$$.

The comparison method is described as follows:

1. In the case where ${\gamma }(\mathcal {H}_1) < {\gamma }(\mathcal {H}_2)$, it follows that $\mathcal {H}_1 \prec \mathcal {H}_2$.
2. When ${\gamma }(\mathcal {H}_1) > {\gamma }(\mathcal {H}_2)$, the relationship $\mathcal {H}_1 \succ \mathcal {H}_2$ holds.
3. In instances where ${\gamma }(\mathcal {H}_1) = {\gamma }(\mathcal {H}_2)$, the following comparisons apply:
  - When $${\lambda }(\mathcal {H}_1) < {\lambda }(\mathcal {H}_2)$$, then $$\mathcal {H}_1 \prec \mathcal {H}_2$$.
  - When $${\lambda }(\mathcal {H}_1) > {\lambda }(\mathcal {H}_2)$$, then $$\mathcal {H}_1 \succ \mathcal {H}_2$$.
  - When ${\lambda }(\mathcal {H}_1) = {\lambda }(\mathcal {H}_2)$, it is concluded that $\mathcal {H}_1 \approx \mathcal {H}_2$.

## Assessment of the proposed MADM approaches in the BCq-ROFNs framework

This section presents a MADM methodology tailored to the BCq-ROFN framework. The proposed method enhances decision-making efficiency by incorporating complex and uncertain information. To validate its effectiveness and practical relevance, real-world case studies are provided, illustrating its application in diverse decision-making contexts.

In numerous MADM problems, a finite number of alternatives, expressed as follows, must be examined by the decision-makers:

$$\{\mathscr{A}\mathscr{L}_1, \mathscr{A}\mathscr{L}_2, \dots , \mathscr{A}\mathscr{L}_{p}\},$$

and a finite set of decision attributes given by:

$$\{\mathscr{A}\mathscr{T}_1, \mathscr{A}\mathscr{T}_2, \dots , \mathscr{A}\mathscr{T}_{k}\}.$$

To facilitate an effective decision-making process, every alternative is evaluated according to the attributes, and a weight vector is assigned to indicate the relative importance of each attribute:

$$\mathscr {X} = (\mathscr {X}_1, \mathscr {X}_2, \dots , \mathscr {X}_{k})^{T},$$

where $\mathscr {X}_i > 0$ and $\sum _{i=1}^{{k}} \mathscr {X}_i = 1$. The decision matrix, capturing evaluations under the BCq-ROFS framework, is constructed as:

$$\mathscr{D}\mathscr{M} = \big [\mathcal {H}_{ji}\big ]_{{p} \times k} = \left[ \langle \mathscr {M}_{\mathcal {H}_{ji}}^{+}+i \mathscr {A}_{\mathcal {H}_{ji}}^{+}, \mathscr {N}_{\mathcal {H}_{ji}}^{+} +i \mathscr {B}_{\mathcal {H}_{ji}}^{+},\mathscr {M}_{\mathcal {H}_{ji}}^{-} +i \mathscr {A}_{\mathcal {H}_{ji}}^{-}, \mathscr {N}_{\mathcal {H}_{ji}}^{-} +i \mathscr {B}_{\mathcal {H}_{ji}}^{-}\rangle \right] _{{p} \times k}$$

14 $$\begin{aligned} =\left[ \begin{array}{cccc} \mathcal {H}_{11} & \mathcal {H}_{12} & \cdots & \mathcal {H}_{1 {k}} \\ \mathcal {H}_{21} & \mathcal {H}_{22} & \cdots & \mathcal {H}_{2 {k}} \\ \vdots & \vdots & & \ddots \\ \mathcal {H}_{p 1} & \mathcal {H}_{p 2} & \cdots & \mathcal {H}_{p {k}} \end{array}\right] , \end{aligned}$$

where $\mathcal {H}_{ji}$ represents the evaluation of alternative $\mathscr{A}\mathscr{L}_j$ ($j = 1, 2, \ldots , p$) relative to the attribute $\mathscr{A}\mathscr{T}_i$ ($i = 1, 2, \ldots , {k}$). Here:

- $\mathscr {M}_{\mathcal {H}_{ji}}^{+}$ signifies the real component linked to positive membership.
- $\mathscr {A}_{\mathcal {H}_{ji}}^{+}$ represents the imaginary component corresponding to positive membership.
- $\mathscr {N}_{\mathcal {H}_{ji}}^{+}$ denotes the real component related to positive non-membership.
- $\mathscr {B}_{\mathcal {H}_{ji}}^{+}$ signifies the imaginary component associated with positive non-membership.
- $\mathscr {M}_{\mathcal {H}_{ji}}^{-}$ refers to the real component linked to negative membership.
- $\mathscr {A}_{\mathcal {H}_{ji}}^{-}$ represents the imaginary component corresponding to negative membership.
- $\mathscr {N}_{\mathcal {H}_{ji}}^{-}$ denotes the real component associated with negative non-membership.
- $\mathscr {B}_{\mathcal {H}_{ji}}^{-}$ signifies the imaginary component related to negative non-membership.

**Algorithm: Decision analysis within the BCq-ROF paradigm**

The decision-making process follows a structured algorithm to systematically assess and rank alternatives. The procedural steps are outlined below:

- Step 1: Problem definition and criteria identification
  - Identify the decision problem, define objectives, and establish the set of alternatives and attributes.
  - Assign importance weights to each attribute based on expert judgment or predefined criteria.

- Step 2: Formulation the bipolar complex decision matrix
  - Populate the decision matrix $\mathscr{D}\mathscr{M}$ with evaluations of each alternative under each attribute in the BCq-ROFN environment.

- Step 3: Standardize the decision matrix
  - Normalize the values to ensure comparability across attributes and maintain consistency in the evaluations.

- Step 4: Apply evaluation operators
  - Calculate the decision values for each alternative using the BCq-ROFWA and BCq-ROFWG operators as follows:

1. $${\text{BCq - WA}}_{{\mathrm{j}}} = \mathrm{BCq} - ROFWA\left( {{\mathcal{H}}_{{j1}} ,{\mathcal{H}}_{{j2}} , \ldots ,{\mathcal{H}}_{{jk}} } \right) = \left\langle {\begin{array}{*{20}l} {\left( {1 - \prod _{{i = 1}}^{k} \left( {1 - \left( {{\mathcal{M}}_{{{\mathcal{H}}_{{jk}} }}^{ + } } \right)^{q} } \right)^{{{\mathcal{X}}_{i} }} } \right)^{{\frac{1}{q}}} + i\left( {1 - \prod _{{i = 1}}^{k} \left( {1 - ({\mathcal{A}}_{{{\mathcal{H}}_{i} }}^{ + } )^{q} } \right)^{{{\mathcal{X}}_{i} }} } \right)^{{\frac{1}{q}}} ,} \hfill \\ {\prod _{{i = 1}}^{k} ({\mathcal{N}}_{{{\mathcal{H}}_{i} }}^{ + } )^{{{\mathcal{X}}_{i} }} + i\prod _{{i = 1}}^{k} ({\mathcal{B}}_{{{\mathcal{H}}_{i} }}^{ + } )^{{{\mathcal{X}}_{i} }} ,} \hfill \\ { - \left( {\prod _{{i = 1}}^{k} |{\mathcal{M}}_{{{\mathcal{H}}_{i} }}^{ - } |^{{{\mathcal{X}}_{i} }} } \right) + i\left( { - \left( {\prod _{{i = 1}}^{k} |{\mathcal{A}}_{{{\mathcal{H}}_{i} }}^{ - } |^{{{\mathcal{X}}_{i} }} } \right)} \right),} \hfill \\ { - \left( {1 - \prod _{{i = 1}}^{k} \left( {1 - |{\mathcal{N}}_{{{\mathcal{H}}_{i} }}^{ - } |^{q} } \right)^{{{\mathcal{X}}_{i} }} } \right)^{{\frac{1}{q}}} + i( - (1 - \prod _{{i = 1}}^{k} (1 - |{\mathcal{B}}_{{{\mathcal{H}}_{i} }}^{ - } |^{q} )^{{{\mathcal{X}}_{i} }} )^{{\frac{1}{q}}} )} \hfill \\ \end{array} } \right\rangle .$$
2. $${\text{BCq - WG}}_{{\mathrm{j}}} = \mathrm{BCq} - ROFWG\left( {{\mathcal{H}}_{{j1}} ,{\mathcal{H}}_{{j2}} , \ldots ,{\mathcal{H}}_{{jk}} } \right) = \left\langle {\begin{array}{*{20}l} {\prod _{{i = 1}}^{k} ({\mathcal{M}}_{{{\mathcal{H}}_{i} }}^{ + } )^{{{\mathcal{X}}_{i} }} + i(\prod _{{i = 1}}^{k} {\mathcal{A}}_{{{\mathcal{H}}_{i} }}^{ + } )^{{{\mathcal{X}}_{i} }} ,} \hfill \\ {(1 - \prod _{{i = 1}}^{k} (1 - ({\mathcal{N}}_{{{\mathcal{H}}_{i} }}^{ + } )^{q} )^{{{\mathcal{X}}_{i} }} )^{{\frac{1}{q}}} + i(1 - \prod _{{i = 1}}^{k} (1 - ({\mathcal{B}}_{{{\mathcal{H}}_{i} }}^{ + } )^{q} )^{{{\mathcal{X}}_{i} }} )^{{\frac{1}{q}}} ,} \hfill \\ { - (1 - \prod _{{i = 1}}^{k} (1 - |{\mathcal{M}}_{{{\mathcal{H}}_{i} }}^{ - } |^{q} )^{{{\mathcal{X}}_{i} }} )^{{\frac{1}{q}}} + i( - (1 - \prod _{{i = 1}}^{k} (1 - |{\mathcal{A}}_{{{\mathcal{H}}_{i} }}^{ - } |^{q} )^{{{\mathcal{X}}_{i} }} )^{{\frac{1}{q}}} ),} \hfill \\ { - (\prod _{{i = 1}}^{k} |{\mathcal{N}}_{{{\mathcal{H}}_{i} }}^{ - } |_{i}^{{{\mathcal{X}}_{i} }} ) + i( - (\prod _{{i = 1}}^{k} |{\mathcal{B}}_{{{\mathcal{H}}_{i} }}^{ - } |_{i}^{{{\mathcal{X}}_{i} }} ))} \hfill \\ \end{array} } \right\rangle .$$

- Step 5: Score computation
  - Derive the final scores for the alternatives according to the ${\mathrm{BCq-WA}_\textrm{j}}$ and ${\mathrm{BCq-WG}_\textrm{j}}$. This step involves synthesizing the scores generated by the computational techniques applied in Step 4 to finalize the overall rating of every alternative. This process involves aggregating the individual scores derived from the evaluation methods used previously, enabling a comprehensive comparison across all options. The resulting final scores represent the overall evaluation of every alternative, allowing for a more rational selection process..

- Step 6: Order the alternatives based on final scores
  - The alternatives should be ordered in decreasing order of their computed scores to determine the most optimal choice. In the final step, the alternative options are ranked according to the evaluation scores calculated in Step 5. This ranking highlights the most favorable options, providing a clear overview of their relative performance based on the established attributes and evaluation process. It serves as a helpful guide to identify the top alternatives.

- Step 7: Output interpretation
  - The final scores obtained for each alternative represent the aggregated evaluation considering all attributes and decision-makers. By ordering these scores in descending order, the most favorable alternatives are identified. The top-ranked alternative indicates the optimal choice, while the full ranking provides a comprehensive view of all alternatives. This information guides decision-makers in selecting the best option and supports transparent, data-driven decision-making by highlighting relative performance across all considered criteria.

This structured methodology provides a comprehensive and rigorous framework for handling multi-attribute decision-making problems within the BCq-ROFN environment, ensuring precise and reliable decision outcomes.

**Fig. 2:**
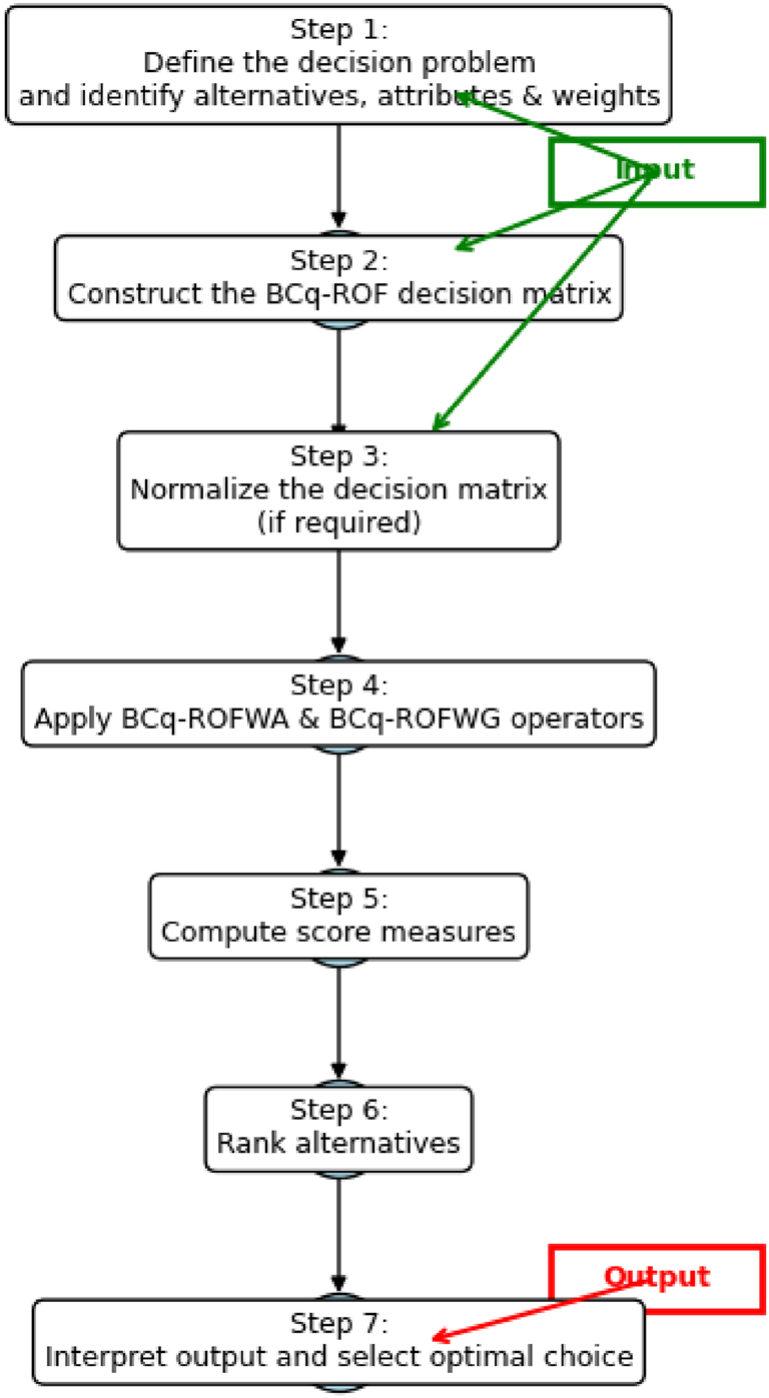
Flowchart of the proposed 7-step BCq-ROFN-based decision-making process.

To facilitate a clear understanding of the BCq-ROFN-based decision-making process, Fig. 2 presents a flowchart illustrating the seven procedural steps. The chart provides a visual overview from problem definition and attribute weighting (Step 1), through decision matrix construction and normalization (Steps 2–3), application of aggregation operators (Step 4), computation of scores (Step 5), ranking of alternatives (Step 6), to the interpretation of the final results (Step 7). This representation aids readers in grasping the systematic workflow and the logical progression of the proposed MADM approach within the BCq-ROF framework.

## Pseudocode representation of the proposed approach

**Algorithm 1:**
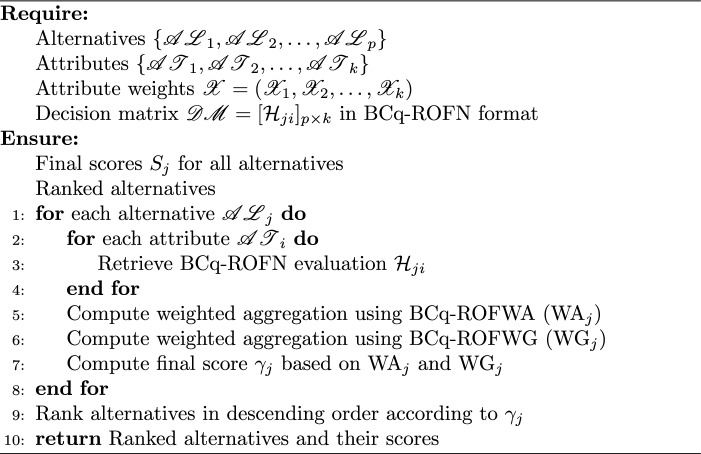
Pseudocode for MADM using BCq-ROFWA and BCq-ROFWG Operators

###  Application of BCq-ROFS to sustainable livestock farming practices

Livestock farming plays a vital role in Saudi Arabia’s agricultural sector, contributing significantly to the country’s food security, rural employment, and economic stability. Despite its importance, the sector faces multiple challenges such as water scarcity, limited feed resources, disease management, and the pressure to increase productivity in a sustainable manner. Given Saudi Arabia’s arid climate, where rainfall is scarce and temperatures are high, the livestock sector heavily depends on irrigation systems and imported feed. This highlights the importance of improving farming methods to support food security and sustainable development over the long term.

The Saudi government has acknowledged the critical need for a sustainable and resilient agricultural sector, as outlined in its Vision 2030 framework. This initiative focuses on enhancing food production systems, including LF, to ensure long-term food security. Vision 2030 aims to reduce the country’s reliance on imported animal products while increasing local production of meat, dairy, and eggs. Furthermore, addressing environmental concerns such as efficient water usage and waste management is crucial, especially given that agriculture accounts for a significant portion of the country’s total water consumption, with estimates indicating that it makes up $67\%$ of total water use^68^.

In this case study, the choice of livestock farming is justified by the inherent two-dimensional nature of the decision problem, where each alternative can be evaluated in terms of positive (supporting) and negative (opposing) aspects for each attribute. The BCq-ROFS framework, with its bipolar complex structure, is particularly suitable for capturing both favorable and unfavorable evaluations simultaneously. This allows for a nuanced and multidimensional assessment of LF practices under uncertainty, aligning perfectly with the theoretical requirements of the proposed aggregation operators.

Different LF alternatives are evaluated using MADM techniques and the BCq-ROFWA and BCq-ROFWG operators to determine the most sustainable and economically feasible practices for LF in Saudi Arabia. The evaluation considers various environmental, economic, and socio-economic factors that impact the sector.

The following alternatives are considered for sustainable LF practices:

### $\mathscr{L}\mathscr{F}_1$: Intensive Livestock Production Systems

Intensive livestock production systems are designed to maximize the efficiency of food production by raising large numbers of animals in confined spaces. While these systems can increase productivity per unit of land, they often face challenges related to resource consumption, such as high water usage and large feed requirements. Additionally, intensive systems may have environmental impacts, such as waste management issues and pollution. In the context of Saudi Arabia, where water scarcity is a significant concern, intensive systems may not be the most sustainable option without improvements in resource management.

### $\mathscr{L}\mathscr{F}_2$: free-range grazing and rotational grazing

Free-range and rotational grazing systems are well-suited for Saudi Arabia’s vast, open landscapes. These systems involve rotating livestock across different grazing areas, which helps maintain soil health and reduces the need for supplemental feed. Free-range grazing can also contribute to better animal welfare, as animals are allowed to roam and express natural behaviors. In a country like Saudi Arabia, where water and feed scarcity are major challenges, rotational grazing systems can help reduce dependency on water-intensive feed crops and improve the sustainability of LF.

### $\mathscr{L}\mathscr{F}_3$: integrated livestock-crop systems

Integrated livestock-crop systems involve the combination of crop and LF, where each component complements the other. For example, crop residues can be used as animal feed, and animal manure can be used as fertilizer for crops. This system promotes efficient resource use, reduces waste, and increases productivity across both sectors. In Saudi Arabia, where land and water resources are limited, integrated systems offer an opportunity to maximize the use of available resources while improving food security and reducing environmental impacts.

### $\mathscr{L}\mathscr{F}_4$: hydroponic feed production

Hydroponic feed production is a soilless method of growing crops, typically using water-based solutions that provide essential nutrients to plants. This method is highly water-efficient, making it ideal for arid regions like Saudi Arabia. Hydroponic systems can produce large quantities of high-quality feed in a relatively small space, reducing dependency on traditional feed crops that require significant water resources. By integrating hydroponic feed production into LF, Saudi Arabia could address water scarcity issues while improving feed availability for livestock.

### $\mathscr{L}\mathscr{F}_5$: vertical farming for livestock feed

Vertical farming refers to cultivating crops on vertically stacked layers or inclined surfaces, offering high efficiency in land utilization. This method is particularly beneficial in urban environments or areas where land is scarce. Vertical farming for livestock feed could be an innovative way to produce animal feed in Saudi Arabia’s cities or arid regions where traditional farming methods are not feasible. By using less water and space, vertical farming could help ensure a local, sustainable supply of feed, reducing the need for imported feed and promoting food security.

### $\mathscr{L}\mathscr{F}_6$: improved animal health and disease management systems

Animal health is a critical factor in LF, as diseases can significantly affect productivity and sustainability. Improved health management systems, which include regular veterinary care, disease prevention, and health monitoring technologies, can enhance the overall well-being of animals and improve the efficiency of LF. In Saudi Arabia, where livestock is an essential component of the agricultural sector, investing in health and disease management systems can reduce the risk of epidemics, improve productivity, and ensure better animal welfare.

### $\mathscr{L}\mathscr{F}_7$: smart farming technologies (IoT, Big Data, AI)

Smart farming technologies, including the Big Data, AI, and IoT, offer a transformative approach to LF by optimizing operations and improving efficiency. IoT devices can monitor animal health, water usage, and environmental conditions in real-time, while AI can analyze this data to make informed decisions on feed, health, and resource management. Big Data can help identify patterns and trends that allow for better decision-making and predictive analytics. In Saudi Arabia, where resource efficiency and sustainability are key priorities, smart farming technologies can help overcome the challenges of water scarcity, feed availability, and climate conditions, making LF more resilient and productive.

The alternatives are evaluated based on eight critical attributes:

$\mathscr {C}_1$: *Water Usage Efficiency* The ability of the farming system to reduce water consumption, which is particularly important in Saudi Arabia’s water-scarce environment. Alternatives such as hydroponic feed production or smart farming can significantly lower water consumption.

$\mathscr {C}_2$: *Feed and Nutrient Efficiency* The efficiency in utilizing feed, with a focus on locally sourced, high-quality feed. Hydroponic systems and integrated livestock-crop systems can reduce reliance on imported feed, thus making the systems more sustainable.

$\mathscr {C}_3$: *Environmental Impact (Waste and Pollution)* The environmental consequences of each farming system, including waste generation and pollution. Intensive farming systems may have a higher environmental footprint compared to free-range or integrated systems.

$\mathscr {C}_4$: *Economic Viability and Cost Efficiency* The economic feasibility of each alternative, considering the capital and operational costs. Intensive systems, while costly to set up, may lead to higher yields and productivity.

$\mathscr {C}_5$: *Technological Integration* The extent to which each system incorporates advanced technologies like IoT, AI, and big data to improve efficiency and animal health. Smart farming systems can optimize production and reduce costs.

$\mathscr {C}_6$: *Animal Welfare* The impact on animal well-being. Free-range systems and systems with better animal health management practices can ensure higher welfare standards.

$\mathscr {C}_7$: *Scalability and Adaptability* The ability of each alternative to be adapted to different scales and regions within Saudi Arabia. For example, systems that integrate crop and LF may be more adaptable to various local conditions.

$\mathscr {C}_8$: *Social and Community Impact* The impact on local communities, including job creation and the improvement of rural livelihoods. Systems that utilize local feed sources or combine agriculture with livestock can improve the economic well-being of rural populations.

For this illustrative case study, a panel of six domain experts was assumed, representing expertise in livestock management, environmental science, renewable energy, circular economy, and socio-economic impact. The panel included two municipal waste management engineers, one environmental scientist, one renewable energy systems engineer, one sustainability policy analyst, and one socio-economic expert. Each expert provided qualitative assessments of seven livestock farming alternatives across eight key attributes. These assessments were systematically translated into the BCq-ROFN framework, capturing both positive and negative evaluations along with uncertainty and hesitancy. Attribute weights were assigned based on expert judgment to reflect the relative importance of each criterion, ensuring a comprehensive and realistic decision-making model.

By applying the BCq-ROFWA and BCq-ROFWG operators to the decision-making process, the best alternatives can be identified and ranked according to their performance across these attributes. The findings of this case study will assist stakeholders in Saudi Arabia’s agricultural sector to make informed decisions regarding the most appropriate and sustainable farming practices. Through the adoption of optimized LF systems, Saudi Arabia can ensure increased food security, reduce dependency on imports, and address the environmental challenges associated with conventional farming methods.

The decision matrix is structured using bipolar complex q-ROFNs. Each entry in this matrix signifies the evaluation of a specific alternative, denoted as $\mathscr{L}\mathscr{F}_j$ ($j = 1, 2, \dots , p$), concerning a given attribute, represented by $\mathscr {C}_i$ ($i = 1, 2, \dots , k$). Mathematically, the decision matrix is formulated as follows:

$$\mathscr{D}\mathscr{M} = \left[ \langle \mathscr {M}_{\mathcal {H}_{ji}}^{+}+i \mathscr {A}_{\mathcal {H}_{ji}}^{+}, \mathscr {N}_{\mathcal {H}_{ji}}^{+} +i \mathscr {B}_{\mathcal {H}_{ji}}^{+},\mathscr {M}_{\mathcal {H}_{ji}}^{-} +i \mathscr {A}_{\mathcal {H}_{ji}}^{-}, \mathscr {N}_{\mathcal {H}_{ji}}^{-} +i \mathscr {B}_{\mathcal {H}_{ji}}^{-}\rangle \right] _{{p} \times k}.$$

The terms within this matrix are explained as follows:

- $\mathscr {M}_{\mathcal {H}_{ji}}^{+}$ quantifies the degree to which an alternative fulfills a specific attribute in a positive sense.
- $\mathscr {N}_{\mathcal {H}_{ji}}^{+}$ measures the extent of non-fulfillment associated with positive membership.
- $\mathscr {M}_{\mathcal {H}_{ji}}^{-}$ represents the level of satisfaction concerning an attribute but in a negative context.
- $\mathscr {N}_{\mathcal {H}_{ji}}^{-}$ indicates the degree of non-fulfillment in relation to negative membership.
- The imaginary-related components, namely $\mathscr {A}_{\mathcal {H}_{ji}}^{+}, \mathscr {B}_{\mathcal {H}_{ji}}^{+}, \mathscr {A}_{\mathcal {H}_{ji}}^{-}, \mathscr {B}_{\mathcal {H}_{ji}}^{-}$, serve to incorporate additional uncertainty factors and contextual preferences into the evaluation process.

In this illustrative case study, the imaginary components of the BCq-ROFN values serve to capture additional dimensions of expert judgment beyond the real-valued membership and non-membership degrees. Specifically, they reflect hesitation, conflicting opinions, or uncertainty that may arise when experts evaluate alternatives across different attributes. For instance, in assessing Water Usage Efficiency, a positive membership of 0.72 may indicate general satisfaction, while an accompanying imaginary term of (0.14)i quantifies subtle hesitation or variability among experts’ opinions. Although these phase terms are illustrative here, they provide a richer representation of the decision context and demonstrate the framework’s capability to handle complex or dynamic information in future, more data-driven applications.

To account for the varying significance of attributes, a weight vector is defined as:

$$\mathscr {X} = (\mathscr {X}_1, \mathscr {X}_2, \dots , \mathscr {X}_{k})^{T},$$

where each weight satisfies $\mathscr {X}_i > 0$ and the sum of all weights equals unity, that is, $\sum _{i=1}^{k} \mathscr {X}_i = 1$. The specific weight values, determined based on stakeholder preferences, are:

$$\mathscr {X} = (.12,.16,.13,.11,.14,.15,.10,.09)^T.$$

Among these, the attribute related to accessibility holds the highest priority ($.16$), followed by attributes concerning feed and nutrient efficiency.

The primary objective of this analysis is to assess various livestock farming practices based on their performance across multiple attributes and identify the most effective practice. The evaluation procedure consists of the following systematic steps:

- The decision matrix is constructed and normalized as shown in Table 1, corresponding to $Step~2$ and $Step~3$ of the algorithm.
- Applying the aggregation operators:
  $${\mathrm{BCq-WA}_\textrm{j}} =\operatorname {BCq-ROFWA}\left( \mathcal {H}_{j1}, \mathcal {H}_{j2}, \dots , \mathcal {H}_{j8}\right) ,$$
  and
  $${\mathrm{BCq-WG}_\textrm{j}} =\operatorname {BCq-ROFWG}\left( \mathcal {H}_{j1}, \mathcal {H}_{j2}, \dots , \mathcal {H}_{j8}\right) ,$$
  to obtain the overall scoring for every alternative. These calculations, carried out using the parameter $q=5$, are summarized in Tables 2 and 3 (corresponding to $Step~4$).
- Determining the final evaluation scores for all alternatives, as presented in Table 4 ($Step~5$).
- Listing the alternatives in order of their computed scores to identify the optimal choice, following Definition 4.12, with results displayed in Table 5 ($Step~6$).

The ordered list of alternatives produced through the $\operatorname {BCq-ROFWA}$ operator with $q=5$ is given by:

$$\mathscr{L}\mathscr{F}_2 \succ \mathscr{L}\mathscr{F}_3 \succ \mathscr{L}\mathscr{F}_4 \succ \mathscr{L}\mathscr{F}_7 \succ \mathscr{L}\mathscr{F}_1 \succ \mathscr{L}\mathscr{F}_6 \succ \mathscr{L}\mathscr{F}_5$$.

Similarly, the ranking obtained using the $\operatorname {BCq-ROFWG}$ operator for the same parameter is:

$$\mathscr{L}\mathscr{F}_2 \succ \mathscr{L}\mathscr{F}_3 \succ \mathscr{L}\mathscr{F}_4 \succ \mathscr{L}\mathscr{F}_7 \succ \mathscr{L}\mathscr{F}_1 \succ \mathscr{L}\mathscr{F}_6 \succ \mathscr{L}\mathscr{F}_5$$.

**Fig. 3:**
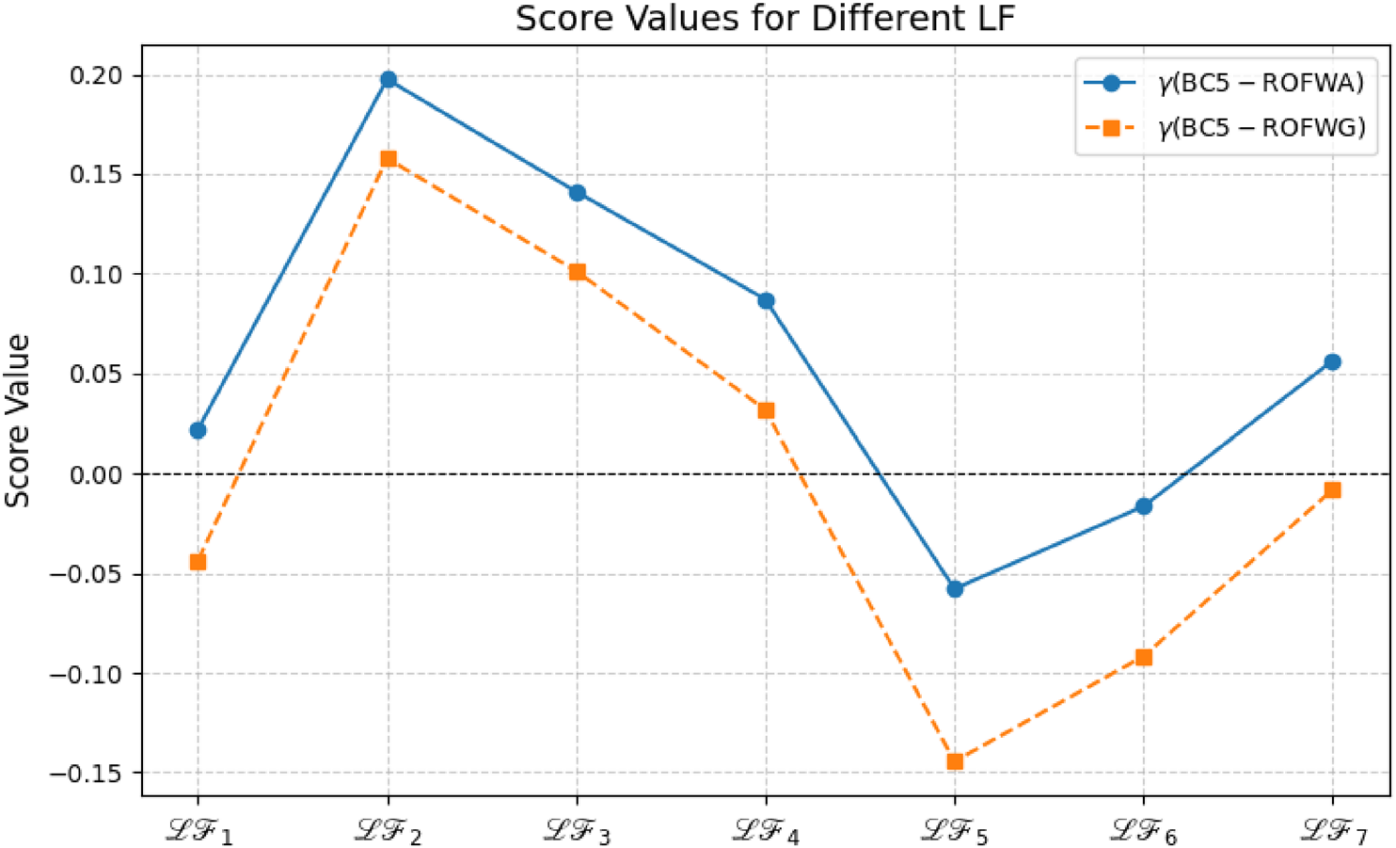
Score values obtained using the BC5-ROFWA and BC5-ROFWG operators.

Both ranking results consistently indicate that $\mathscr{L}\mathscr{F}_2$ is the most suitable livestock farming practice. Figure 3 presents a visual comparison of the ranking outcomes for the alternatives $\mathscr{L}\mathscr{F}_1$ to $\mathscr{L}\mathscr{F}_7$, as evaluated using the $\operatorname {BC5-ROFWA}$ and $\operatorname {BC5-ROFWG}$ operators. The plot displays the computed score values for each alternative, with circles representing the weighted averaging operator and squares representing the weighted geometric operator. The horizontal dashed line at zero indicates the neutral reference point, facilitating the identification of positive and negative preference scores.

**Table 1:** BC5-ROFS values.

LF	$\mathscr {C}_1$	$\mathscr {C}_2$
$\mathscr{L}\mathscr{F}_1$	$\langle .95 +.85 i,.15 +.25 i, -.60 +(-.58)i, -.42 +(-.59)i\rangle$	$\langle .67 +.33 i,.73 +.74 i, -.58 +(-.61)i, -.41 +(-.72)i\rangle$
$\mathscr{L}\mathscr{F}_2$	$\langle .99 +.89 i,.11 +.21 i, -.87 +(-.91)i, -.12 +(-.19)i\rangle$	$\langle .87 +.46 i,.45 +.61 i, -.79 +(-.82)i, -.25 +(-.51)i\rangle$
$\mathscr{L}\mathscr{F}_3$	$\langle .98 +.88 i,.12 +.22 i, -.83 +(-.89)i, -.15 +(-.22)i\rangle$	$\langle .81 +.42 i,.52 +.63 i, -.71 +(-.76)i, -.29 +(-.64)i\rangle$
$\mathscr{L}\mathscr{F}_4$	$\langle .97 +.87 i,.13 +.23 i, -.71 +(-.75)i, -.23 +(-.31)i\rangle$	$\langle .72 +.37 i,.58 +.70 i, -.65 +(-.73)i, -.31 +(-.68)i\rangle$
$\mathscr{L}\mathscr{F}_5$	$\langle .93 +.83 i,.17 +.27 i, -.49 +(-.31)i, -.69 +(-.78)i\rangle$	$\langle .12 +.19 i,.91 +.81 i, -.29 +(-.39)i, -.52 +(-.81)i\rangle$
$\mathscr{L}\mathscr{F}_6$	$\langle .94 +.84 i,.16 +.26 i, -.58 +(-.44)i, -.56 +(-.67)i\rangle$	$\langle .55 +.24 i,.81 +.78 i, -.51 +(-.50)i, -.44 +(-.77)i\rangle$
$\mathscr{L}\mathscr{F}_7$	$\langle .96 +.86 i,.14 +.24 i, -.65 +(-.61)i, -.35 +(-.42)i\rangle$	$\langle .71 +.34 i,.62 +.71 i, -.61 +(-.65)i, -.37 +(-.71)i\rangle$
LF	$\mathscr {C}_3$	$\mathscr {C}_4$
$\mathscr{L}\mathscr{F}_1$	$\langle .32 +.51 i,.79 +.65 i, -.12 +(-.61)i, -.69 +(-.38)i\rangle$	$\langle .24 +.53 i,.60 +.31 i, -.23 +(-.65)i, -.62 +(-.43)i\rangle$
$\mathscr{L}\mathscr{F}_2$	$\langle .49 +.70 i,.60 +.52 i, -.23 +(-.77)i, -.54 +(-.12)i\rangle$	$\langle .39 +.76 i,.29 +.12 i, -.39 +(-.82)i, -.41 +(-.09)i\rangle$
$\mathscr{L}\mathscr{F}_3$	$\langle .44 +.65 i,.63 +.60 i, -.21 +(-.75)i, -.56 +(-.19)i\rangle$	$\langle .36 +.72 i,.34 +.15 i, -.34 +(-.79)i, -.43 +(-.11)i\rangle$
$\mathscr{L}\mathscr{F}_4$	$\langle .41 +.61 i,.71 +.61 i, -.20 +(-.72)i, -.58 +(-.24)i\rangle$	$\langle .33 +.64 i,.41 +.19 i, -.27 +(-.71)i, -.58 +(-.16)i\rangle$
$\mathscr{L}\mathscr{F}_5$	$\langle .24 +.32 i,.82 +.70 i, -.04 +(-.42)i, -.74 +(-.57)i\rangle$	$\langle .15 +.31 i,.70 +.51 i, -.16 +(-.11)i, -.64 +(-.55)i\rangle$
$\mathscr{L}\mathscr{F}_6$	$\langle .30 +.49 i,.82 +.68 i, -.10 +(-.53)i, -.71 +(-.40)i\rangle$	$\langle .19 +.35 i,.63 +.40 i, -.19 +(-.14)i, -.63 +(-.50)i\rangle$
$\mathscr{L}\mathscr{F}_7$	$\langle .35 +.59 i,.78 +.62 i, -.15 +(-.68)i, -.61 +(-.32)i\rangle$	$\langle .30 +.63 i,.59 +.24 i, -.25 +(-.68)i, -.59 +(-.21)i\rangle$
LF	$\mathscr {C}_5$	$\mathscr {C}_6$
$\mathscr{L}\mathscr{F}_1$	$\langle .41 +.37 i,.53 +.50 i, -.61 +(-.32)i, -.19 +(-.53)i\rangle$	$\langle .08 +.65 i,.23 +.29 i, -.46 +(-.54)i, -.32 +(-.69)i\rangle$
$\mathscr{L}\mathscr{F}_2$	$\langle .74 +.48 i,.21 +.32 i, -.90 +(-.54)i, -.11 +(-.32)i\rangle$	$\langle .19 +.81 i,.09 +.03 i, -.57 +(-.71)i, -.01 +(-.43)i\rangle$
$\mathscr{L}\mathscr{F}_3$	$\langle .69 +.43 i,.34 +.37 i, -.81 +(-.49)i, -.13 +(-.43)i\rangle$	$\langle .17 +.80 i,.13 +.10 i, -.53 +(-.69)i, -.12 +(-.52)i\rangle$
$\mathscr{L}\mathscr{F}_4$	$\langle .51 +.41 i,.42 +.41 i, -.73 +(-.44)i, -.16 +(-.52)i\rangle$	$\langle .12 +.73 i,.16 +.14 i, -.51 +(-.63)i, -.17 +(-.53)i\rangle$
$\mathscr{L}\mathscr{F}_5$	$\langle .22 +.18 i,.67 +.55 i, -.57 +(-.23)i, -.22 +(-.63)i\rangle$	$\langle .01 +.45 i,.34 +.41 i, -.36 +(-.39)i, -.49 +(-.77)i\rangle$
$\mathscr{L}\mathscr{F}_6$	$\langle .27 +.24 i,.54 +.52 i, -.58 +(-.24)i, -.20 +(-.62)i\rangle$	$\langle .03 +.58 i,.28 +.33 i, -.41 +(-.47)i, -.46 +(-.75)i\rangle$
$\mathscr{L}\mathscr{F}_7$	$\langle .42 +.39 i,.50 +.49 i, -.64 +(-.37)i, -.18 +(-.53)i\rangle$	$\langle .10 +.70 i,.19 +.24 i, -.49 +(-.60)i, -.31 +(-.62)i\rangle$
LF	$\mathscr {C}_7$	$\mathscr {C}_8$
$\mathscr{L}\mathscr{F}_1$	$\langle .58 +.21 i,.81 +.67 i, -.08 +(-.15)i, -.73 +(-.41)i\rangle$	$\langle .38 +.32 i,.59 +.59 i, -.23 +(-.24)i, -.59 +(-.80)i\rangle$
$\mathscr{L}\mathscr{F}_2$	$\langle .81 +.38 i,.52 +.52 i, -.19 +(-.29)i, -.51 +(-.11)i\rangle$	$\langle .80 +.59 i,.31 +.42 i, -.42 +(-.67)i, -.38 +(-.65)i\rangle$
$\mathscr{L}\mathscr{F}_3$	$\langle .74 +.33 i,.53 +.56 i, -.13 +(-.25)i, -.55 +(-.19)i\rangle$	$\langle .63 +.55 i,.45 +.45 i, -.34 +(-.54)i, -.46 +(-.69)i\rangle$
$\mathscr{L}\mathscr{F}_4$	$\langle .63 +.29 i,.76 +.61 i, -.12 +(-.21)i, -.58 +(-.22)i\rangle$	$\langle .51 +.46 i,.52 +.53 i, -.28 +(-.39)i, -.55 +(-.74)i\rangle$
$\mathscr{L}\mathscr{F}_5$	$\langle .35 +.14 i,.83 +.75 i, -.01 +(-.03)i, -.78 +(-.51)i\rangle$	$\langle .11 +.20 i,.63 +.72 i, -.12 +(-.20)i, -.61 +(-.82)i\rangle$
$\mathscr{L}\mathscr{F}_6$	$\langle .46 +.17 i,.83 +.71 i, -.06 +(-.12)i, -.74 +(-.50)i\rangle$	$\langle .19 +.23 i,.62 +.69 i, -.14 +(-.23)i, -.60 +(-.81)i\rangle$
$\mathscr{L}\mathscr{F}_7$	$\langle .60 +.24 i,.77 +.63 i, -.10 +(-.17)i, -.62 +(-.32)i\rangle$	$\langle .38 +.42 i,.58 +.55 i, -.25 +(-.36)i, -.57 +(-.78)i\rangle$

**Table 2:** Aggregated BC5-ROFWA information matrix.

**LF**	BC5-ROFWA
$\mathscr{L}\mathscr{F}_1$	$\langle .7177 +.6248 i,.4758 +.4632 i, -.3147 +(-.4377)i, -.5778 +(-.6408)i\rangle$
$\mathscr{L}\mathscr{F}_2$	$\langle .8509 +.7303 i,.2604 +.2433 i, -.5004 +(-.6728)i, -.4109 +(-.4539)i\rangle$
$\mathscr{L}\mathscr{F}_3$	$\langle .8045 +.7097 i,.3230 +.3182 i, -.4384 +(-.6245)i, -.4387 +(-.5270)i\rangle$
$\mathscr{L}\mathscr{F}_4$	$\langle .7588 +.6712 i,.3840 +.3654 i, -.3926 +(-.5542)i, -.4888 +(-.5664)i\rangle$
$\mathscr{L}\mathscr{F}_5$	$\langle .6687 +.5730 i,.5650 +.5560 i, -.1675 +(-.2255)i, -.6447 +(-.7289)i\rangle$
$\mathscr{L}\mathscr{F}_6$	$\langle .6898 +.5983 i,.5122 +.5077 i, -.2662 +(-.3117)i, -.6020 +(-.6889)i\rangle$
$\mathscr{L}\mathscr{F}_7$	$\langle .7375 +.6548 i,.4386 +.4235 i, -.3484 +(-.4937)i, -.5153 +(-.6060)i\rangle$

**Table 3:** Aggregated BC5-ROFWG information matrix.

**LF**	BC5-ROFWG
$\mathscr{L}\mathscr{F}_1$	$\langle .3604 +.4419 i,.6738 +.6022 i, -.5130 +(-.5566)i, -.4361 +(-.5553)i\rangle$
$\mathscr{L}\mathscr{F}_2$	$\langle .5757 +.6106 i,.4466 +.4760 i, -.7535 +(-.7771)i, -.1639 +(-.2483)i\rangle$
$\mathscr{L}\mathscr{F}_3$	$\langle .5248 +.5697 i,.4839 +.5115 i, -.6818 +(-.7418)i, -.2652 +(-.3238)i\rangle$
$\mathscr{L}\mathscr{F}_4$	$\langle .4434 +.5195 i,.5876 +.5574 i, -.6011 +(-.6642)i, -.3246 +(-.3849)i\rangle$
$\mathscr{L}\mathscr{F}_5$	$\langle .1425 +.2819 i,.7708 +.6758 i, -.4214 +(-.3462)i, -.5349 +(-.6761)i\rangle$
$\mathscr{L}\mathscr{F}_6$	$\langle .2519 +.3505 i,.7144 +.6440 i, -.4772 +(-.4382)i, -.4896 +(-.6166)i\rangle$
$\mathscr{L}\mathscr{F}_7$	$\langle .3931 +.4886 i,.6361 +.5730 i, -.5451 +(-.6011)i, -.3979 +(-.4604)i\rangle$

**Table 4:** Score values.

**LF**	$\gamma (\operatorname {BC5-ROFWA})$	$\gamma (\operatorname {BC5-ROFWG})$
$\mathscr{L}\mathscr{F}_1$	.0217	$\operatorname {-.0437}$
$\mathscr{L}\mathscr{F}_2$	.1975	.1578
$\mathscr{L}\mathscr{F}_3$	.1411	.1013
$\mathscr{L}\mathscr{F}_4$	.0871	.0317
$\mathscr{L}\mathscr{F}_5$	− .0579	− .1445
$\mathscr{L}\mathscr{F}_6$	− .0165	− .0917
$\mathscr{L}\mathscr{F}_7$	.0563	− .0082

**Table 5:** Alternative Rankings for the Case Study.

Techniques	Order of ranking	Optimal choice
BC5-ROFWA	$\mathscr{L}\mathscr{F}_2 \succ \mathscr{L}\mathscr{F}_3 \succ \mathscr{L}\mathscr{F}_4 \succ \mathscr{L}\mathscr{F}_7 \succ \mathscr{L}\mathscr{F}_1 \succ \mathscr{L}\mathscr{F}_6 \succ \mathscr{L}\mathscr{F}_5$	$\mathscr{L}\mathscr{F}_2$
BC5-ROFWG	$\mathscr{L}\mathscr{F}_2 \succ \mathscr{L}\mathscr{F}_3 \succ \mathscr{L}\mathscr{F}_4 \succ \mathscr{L}\mathscr{F}_7 \succ \mathscr{L}\mathscr{F}_1 \succ \mathscr{L}\mathscr{F}_6 \succ \mathscr{L}\mathscr{F}_5$	$\mathscr{L}\mathscr{F}_2$

### Evaluation of parameter influence

In this subsection, the impact of the parameter *q* on the behavior and performance of the proposed bipolar complex q-rung orthopair fuzzy aggregation operators is systematically investigated. The parameter *q* plays a crucial role in regulating the level of flexibility and compensation within the aggregation process. Specifically, smaller values of *q* yield more restrictive (non-compensatory) aggregation behavior, while larger *q* values allow greater compensation between membership and nonmembership degrees.

To evaluate this effect, multiple instances of *q* were considered within the BCq-ROFWA and BCq-ROFWG operators. The outcomes of this analysis are presented in Tables 6 and 7. For each operator, nine distinct *q* values $q = 7, 9, 12, 15, 18, 20, 22, 24, 27$ were employed to observe how the computed scores and corresponding rankings of alternatives evolve as *q* varies. This wide range of *q* values allows for a comprehensive sensitivity assessment—ranging from conservative to highly compensatory decision-making behavior.

As shown in Table 6, the score values obtained from the BCq-ROFWA operator gradually decrease as *q* increases, reflecting a diminishing influence of higher membership degrees in the aggregation process. A similar pattern is observed in Table 7 for the BCq-ROFWG operator, where the scores consistently decrease in real with increasing *q*, approaching a stable and smoother distribution. These trends confirm that the operators maintain monotonic consistency with respect to *q*: while the absolute scores vary, the relative ranking of alternatives remains unchanged.

Importantly, in all tested scenarios, the best alternative ($\mathscr{L}\mathscr{F}_2$) remains invariant across all *q* values. This invariance demonstrates the isotonic stability of the proposed operators—indicating that although the relative reals of scores are sensitive to *q*, the final decision outcome remains robust. Consequently, decision-makers can select the most appropriate *q* value to match their desired level of caution or optimism, without the risk of altering the optimal choice.

**Fig. 4:**
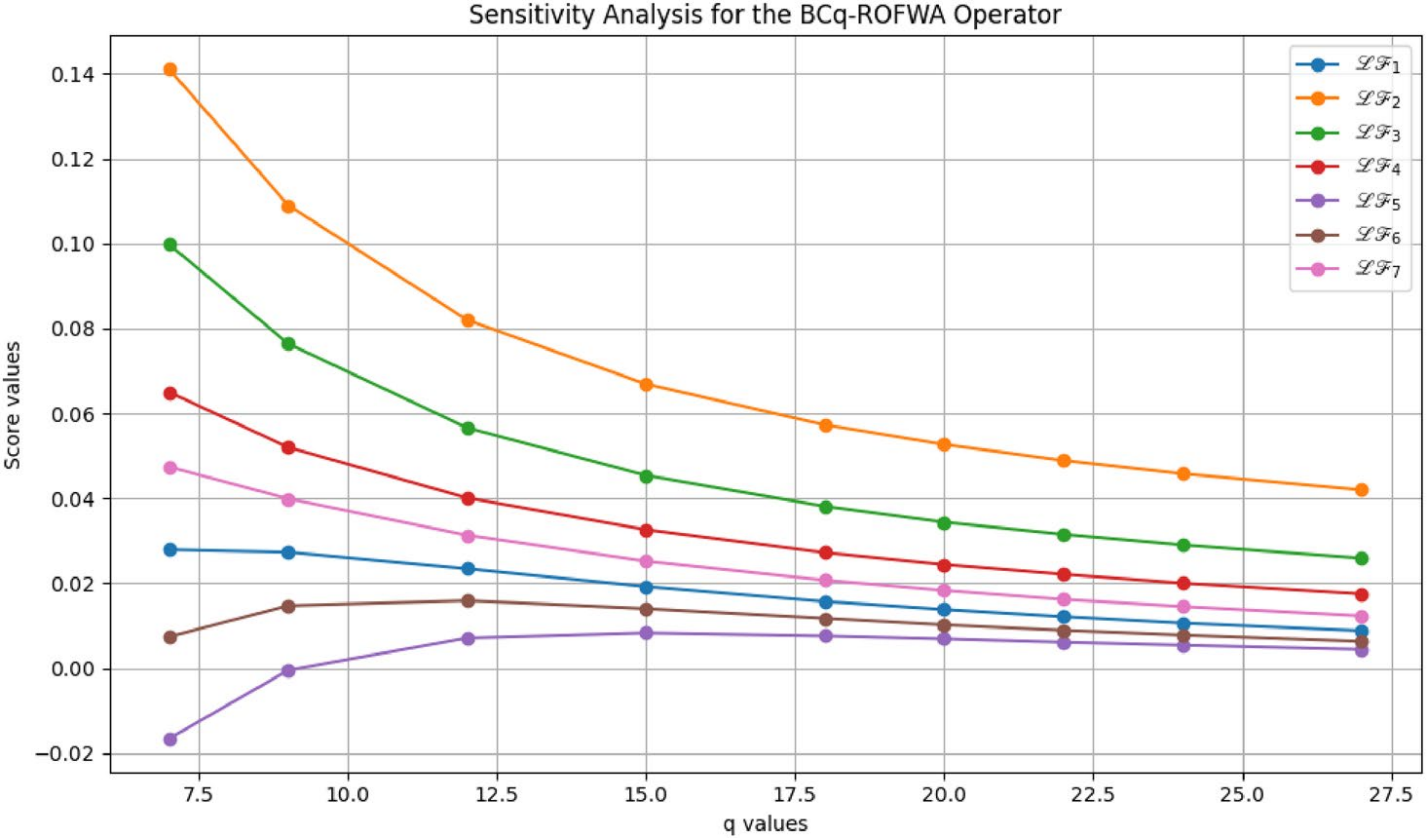
Stability of rankings under varying *q* for BCq-ROFWA.

**Fig. 5:**
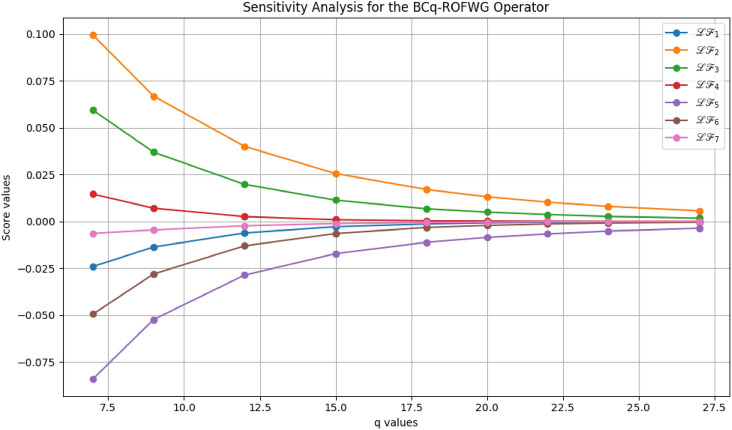
Stability of rankings under varying *q* for BCq-ROFWG.

The graphical representations in Figs. 4 and 5 further illustrate the influence of varying *q* on the aggregated results. These plots highlight how the ranking order is preserved while the numerical differences between alternatives become smoother as *q* increases. Such visual evidence complements the numerical findings, offering an intuitive understanding of the operators’ stability and adaptability.

**Table 6:** Sensitivity analysis results for the BCq-ROFWA operator.

*q*	Score values of							Option
	$\mathscr{L}\mathscr{F}_1$	$\mathscr{L}\mathscr{F}_2$	$\mathscr{L}\mathscr{F}_3$	$\mathscr{L}\mathscr{F}_4$	$\mathscr{L}\mathscr{F}_5$	$\mathscr{L}\mathscr{F}_6$	$\mathscr{L}\mathscr{F}_7$	
7	.0279	.1411	.0998	.0650	− .0166	.0074	.0474	$\mathscr{L}\mathscr{F}_2$
9	.0273	.1090	.0764	.0520	− .0005	.0147	.0399	$\mathscr{L}\mathscr{F}_2$
12	.0234	.0820	.0566	.0401	.0071	.0159	.0313	$\mathscr{L}\mathscr{F}_2$
15	.0192	.0669	.0454	.0326	.0083	.0140	.0252	$\mathscr{L}\mathscr{F}_2$
18	.0157	.0573	.0381	.0272	.0076	.0117	.0207	$\mathscr{L}\mathscr{F}_2$
20	.0138	.0527	.0344	.0244	.0069	.0102	.0183	$\mathscr{L}\mathscr{F}_2$
22	.0121	.0489	.0315	.0221	.0061	.0089	.0162	$\mathscr{L}\mathscr{F}_2$
24	.0106	.0458	.0290	.0200	.0054	.0078	.0145	$\mathscr{L}\mathscr{F}_2$
27	.0088	.0420	.0259	.0175	.0044	.0063	.0123	$\mathscr{L}\mathscr{F}_2$

**Table 7:** Sensitivity analysis results for the BCq-ROFWG operator.

*q*	Score values of							Option
	$\mathscr{L}\mathscr{F}_1$	$\mathscr{L}\mathscr{F}_2$	$\mathscr{L}\mathscr{F}_3$	$\mathscr{L}\mathscr{F}_4$	$\mathscr{L}\mathscr{F}_5$	$\mathscr{L}\mathscr{F}_6$	$\mathscr{L}\mathscr{F}_7$	
7	− 0.0241	0.0994	0.0593	0.0145	− 0.0841	− 0.0495	− 0.0064	$\mathscr{L}\mathscr{F}_2$
9	− 0.0137	0.0668	0.0369	0.0070	− 0.0524	− 0.0281	− 0.0046	$\mathscr{L}\mathscr{F}_2$
12	− 0.0062	0.0400	0.0197	0.0025	− 0.0287	− 0.0131	− 0.0024	$\mathscr{L}\mathscr{F}_2$
15	− 0.0029	0.0255	0.0113	0.0009	− 0.0173	− 0.0065	− 0.0012	$\mathscr{L}\mathscr{F}_2$
18	− 0.0014	0.0170	0.0067	0.0003	− 0.0112	− 0.0033	− 0.0006	$\mathscr{L}\mathscr{F}_2$
20	− 0.0008	0.0131	0.0049	0.0002	− 0.0086	− 0.0022	− 0.0004	$\mathscr{L}\mathscr{F}_2$
22	− 0.0005	0.0102	0.0036	0.0001	− 0.0067	− 0.0014	− 0.0002	$\mathscr{L}\mathscr{F}_2$
24	− 0.0003	0.0080	0.0026	0.0000	− 0.0052	− 0.0009	− 0.0001	$\mathscr{L}\mathscr{F}_2$
27	− 0.0002	0.0056	0.0017	0.0000	− 0.0037	− 0.0005	− 0.0001	$\mathscr{L}\mathscr{F}_2$

## Comparison of our models with existing operators

In this section, we present a thorough comparative analysis between the newly formulated bipolar complex q-rung orthopair fuzzy weighted averaging (BCq-ROFWA) and weighted geometric (BCq-ROFWG) operators and a variety of aggregation mechanisms already established in the literature. The objective of this comparison is to highlight the broader applicability and generality of the proposed models across multiple fuzzy set extensions. Among the conventional operators, the BCIFWA and BCIFWG operators were specifically tested in the bipolar complex domain. In our evaluations, both operators failed to generate valid outputs. The failure is due to the combined membership and nonmembership degrees in several alternatives exceeding the permissible field limit of 1. This limitation demonstrates that BCIFWA and BCIFWG are not always applicable in complex-valued bipolar fuzzy scenarios, restricting their practical usability. In contrast, the proposed BCq-ROFWA and BCq-ROFWG operators are designed to enforce the field condition, ensuring that all aggregated values remain valid across every tested scenario.

Our investigation considers several particular scenarios by constraining certain parameters of the general model. Specifically, cases are analyzed where $\mathscr {A}_{\mathcal {H}_{ji}}^{+}, \mathscr {B}_{\mathcal {H}_{ji}}^{+}, \mathscr {M}_{\mathcal {H}_{ji}}^{-}, \mathscr {A}_{\mathcal {H}_{ji}}^{-}, \mathscr {N}_{\mathcal {H}_{ji}}^{-},$ and $\mathscr {B}_{\mathcal {H}_{ji}}^{-}$ take zero values. Furthermore, bipolar extensions are considered when $\mathscr {A}_{\mathcal {H}_{ji}}^{+}, \mathscr {B}_{\mathcal {H}_{ji}}^{+}, \mathscr {A}_{\mathcal {H}_{ji}}^{-},$ and $\mathscr {B}_{\mathcal {H}_{ji}}^{-}$ are restricted to zero. These reductions confirm that the proposed BCq-ROFWA and BCq-ROFWG operators naturally embed and unify existing operators, thus providing an umbrella model that encompasses IFSs, PFSs, FFSs, BPFSs, Bq-ROFSs, Cq-ROFSs, and BCFSs. This unified framework ensures a seamless transition between classical and more sophisticated fuzzy environments.

## Main findings and benefits

**Fig. 6:**
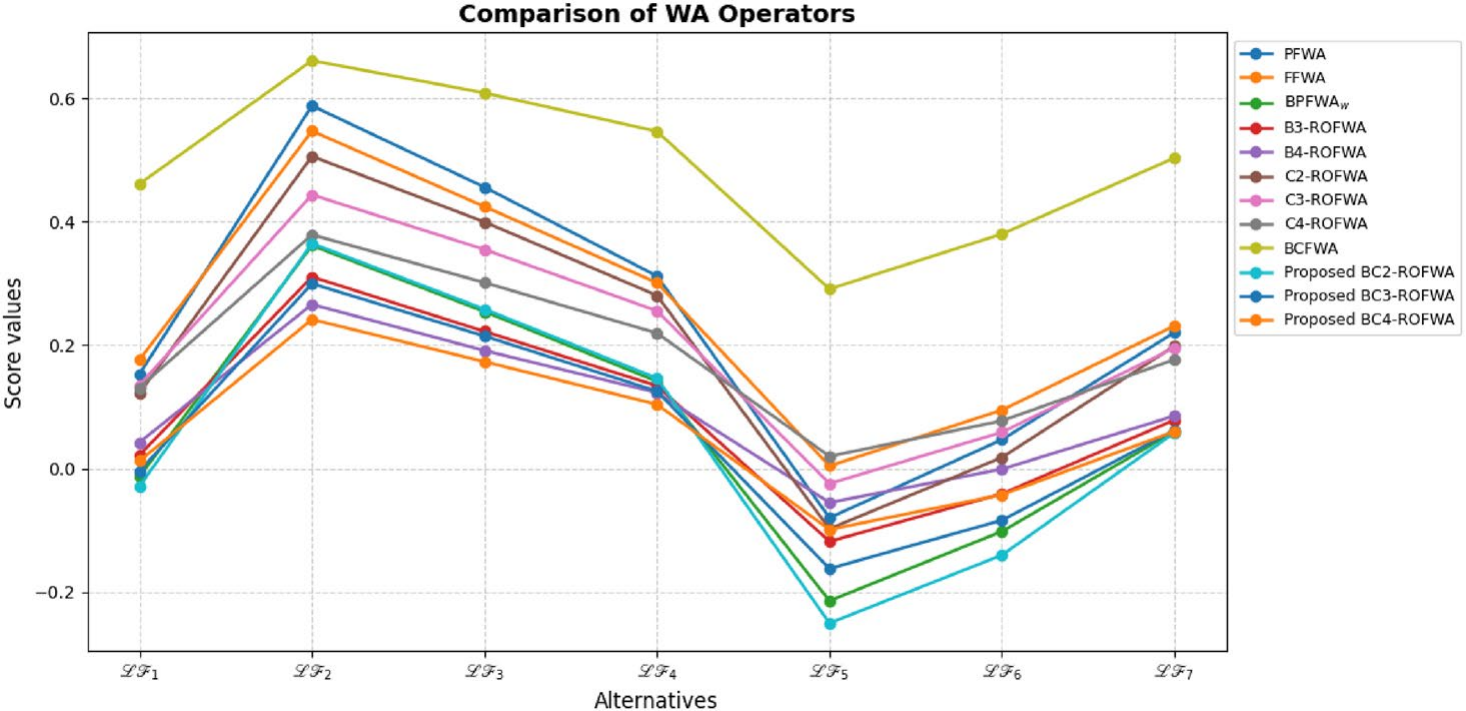
Visualization of the comparative evaluation results (WA).

**Fig. 7:**
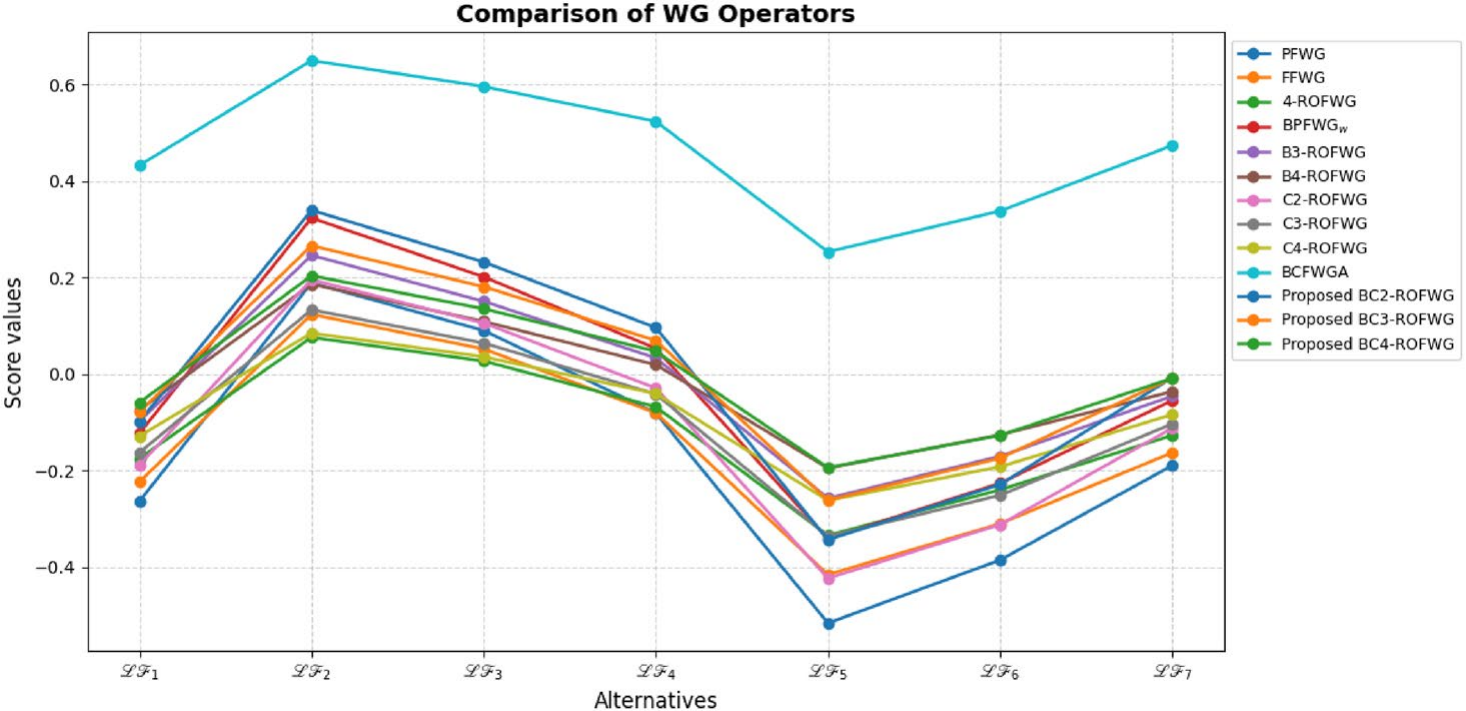
Visualization of the comparative evaluation results (WG).

The central novelty of this work is the design and application of BCq-ROFWA and BCq-ROFWG operators, which substantially extend the expressive power of aggregation techniques in the fuzzy decision-making literature. The performance of the suggested operators has been validated through comparative results: the calculated scores of alternatives are presented in Table 8, while Figs. 6 and 7 provides a visual illustration of the ranking differences and similarities. The integration of complex-valued and bipolar structures equips the proposed model with the capacity to handle multidimensional uncertainty, offering richer insights in MADM contexts.

The primary advantages of this study can be outlined as follows:

1. Generalization of existing operators. The proposed BCq-ROFWA and BCq-ROFWG models act as a superset of several existing fuzzy aggregation frameworks, each emerging as a special case under certain constraints:
  Classical intuitionistic, Pythagorean, Fermatean and q-rung orthopair fuzzy cases: When imaginary and negative parts are absent, the model reduces to IF weighted averaging (IFWA)^69^, IF weighted geometric (IFWG)^70^, Pythagorean fuzzy weighted averaging (PFWA), Pythagorean fuzzy weighted geometric (PFWG)^71^, Fermatean fuzzy weighted averaging (FFWA)^72^, Fermatean fuzzy weighted geometric (FFWG)^73^, and q-ROF weighted geometric (q-ROFWG)^74^ operators.
  Bipolar Pythagorean and bipolar q-rung orthopair fuzzy cases: When imaginary parts alone are omitted, the framework reduces to BPF weighted average (BPFWA$\phantom{0}_{w}$) and weighted geometric (BPFWG$\phantom{0}_{w}$) operators^42^, along with the bipolar q-ROFWA (Bq-ROFWA) and bipolar q-ROFWG (Bq-ROFWG) operators^15^, extend the advantages of bipolarity to the Pythagorean and q-ROF frameworks, respectively. For the BPFWA$\phantom{0}_{w}$ and BPFWG$\phantom{0}_{w}$ operators, we adopt the score function introduced in^14^:
    $${\gamma }(\mathcal {H}) = \frac{1}{2} \left( \left[ (\mathscr {M}_{\mathcal {H}}^{+})^{2} - (\mathscr {N}_{\mathcal {H}}^{+})^{2} \right] + \left[ |\mathscr {M}_{\mathcal {H}}^{-}|^{2} - |\mathscr {N}_{\mathcal {H}}^{-}|^{2} \right] \right) .$$
    Similarly, for the Bq-ROFWA and Bq-ROFWG operators, we employ the score function:
    $${\gamma }(\mathcal {H}) = \frac{1}{2} \left( \left[ (\mathscr {M}_{\mathcal {H}}^{+})^{q} - (\mathscr {N}_{\mathcal {H}}^{+})^{q} \right] + \left[ |\mathscr {M}_{\mathcal {H}}^{-}|^{q} - |\mathscr {N}_{\mathcal {H}}^{-}|^{q} \right] \right) .$$
  Complex q-rung orthopair fuzzy cases: When negative parts alone are omitted, the framework reduces to Cq-ROFWA and Cq-ROFWG operators^9^, which extend the model to handle complex-valued membership information. In this case, the real and imaginary components of the membership and non-membership degrees are interpreted as the amplitude and phase terms, respectively.
  Bipolar complex fuzzy cases: When the real and imaginary components of nonmembership functions vanish simultaneously in both positive and negative evaluations, the model specializes to the bipolar complex fuzzy weighted averaging (BCFWAA) and geometric (BCFWGA) operators introduced in^75^. For the BCFWAA and BCFWGA operators, we employ the score function:
    $${\gamma }(\mathcal {H}) = \frac{1}{4} ( \mathscr {M}_{\mathcal {H}}^{+} + \mathscr {A}_{\mathcal {H}}^{+} + |\mathscr {M}_{\mathcal {H}}^{-}| + |\mathscr {A}_{\mathcal {H}}^{-}|).$$
  This chain of reductions verifies that the proposed model is highly flexible and general, capturing a wide spectrum of fuzzy set environments.
2. Comprehensive representation of uncertainty. Unlike traditional models, the BCq-ROFWA and BCq-ROFWG operators incorporate multiple dimensions of uncertainty—positive and negative assessments, hesitation degrees, and complex-valued membership structures—within a unified decision-making framework. This allows for a more realistic and nuanced modeling of uncertain information, especially in contexts involving conflicting, bipolar, or imprecise evaluations.
3. Enhanced comparative insights. The experimental results (Table 8) and their visual comparison (Figs. 6 and 7) demonstrate that the suggested operators not only align with the outcomes of established methods but also provide distinct variations in rankings. This suggests that BCq-ROFWA and BCq-ROFWG introduce new interpretive dimensions for decision-makers while ensuring backward compatibility with classical intuitionistic and bipolar fuzzy operators.

Visualizations (Figs. 6 and 7) complement the numerical results, highlighting performance differences across aggregation operators and facilitating intuitive understanding of rankings. All tested operators, including the proposed BCq-ROFWA and BCq-ROFWG, consistently identify the same optimal alternative, confirming reliability and robustness.

## Key strengths

- Greater expressiveness: Integration of q-rung orthopair fuzzy sets, bipolarity, and complex numbers enhances uncertainty representation.
- Superior decision-making capability: Complex numbers capture periodic or oscillatory behaviors, beneficial for dynamic data contexts.
- Enhanced adaptability: The proposed operators accommodate a wider range of decision-making scenarios than conventional fuzzy aggregation operators.

**Table 8:** Comparison of existing aggregation operators with proposed BCq-ROF operators.

Operators	Score values of							Option
	$\mathscr{L}\mathscr{F}_1$	$\mathscr{L}\mathscr{F}_2$	$\mathscr{L}\mathscr{F}_3$	$\mathscr{L}\mathscr{F}_4$	$\mathscr{L}\mathscr{F}_5$	$\mathscr{L}\mathscr{F}_6$	$\mathscr{L}\mathscr{F}_7$	
IFWA^69^	–	–	–	–	–	–	–	Failed
PFWA^76^	.1530	.5876	.4558	.3119	− 0.0797	0.0469	.2204	$\mathscr{L}\mathscr{F}_2$
FFWA^72^	.1760	.5468	.4243	.3009	0.0043	0.0947	.2312	$\mathscr{L}\mathscr{F}_2$
BPFWA$\phantom{0}_{w}$^42^	− 0.0131	.3612	.2543	.1413	− .2142	− .1017	0.0601	$\mathscr{L}\mathscr{F}_2$
B3-ROFWA^15^	0.0219	.3105	.2221	.1341	− .1179	− 0.0408	0.0787	$\mathscr{L}\mathscr{F}_2$
B4-ROFWA^15^	0.0425	.2659	.1911	.1229	− 0.0550	− 0.0009	0.0854	$\mathscr{L}\mathscr{F}_2$
C2-ROFWA^9^	.1221	.5057	.3994	.2804	− 0.0975	0.0181	.1985	$\mathscr{L}\mathscr{F}_2$
C3-ROFWA^9^	.1353	.4434	.3548	.2555	− 0.0243	0.0589	.1956	$\mathscr{L}\mathscr{F}_2$
C4-ROFWA^9^	.1312	.3787	.3012	.2194	0.0199	0.0774	.1765	$\mathscr{L}\mathscr{F}_2$
BCFWAA^75^	.4611	.6608	.6084	.5462	.2908	.3799	.5031	$\mathscr{L}\mathscr{F}_2$
BCIFWA^20^	–	–	–	–	–	–	–	Failed
Proposed BC2-ROFWA	− 0.0288	.3645	.2582	.1464	− .2503	− .1401	0.0581	$\mathscr{L}\mathscr{F}_2$
Proposed BC3-ROFWA	− 0.0041	.2995	.2146	.1253	− .1620	− 0.0836	0.0621	$\mathscr{L}\mathscr{F}_2$
Proposed BC4-ROFWA	0.0124	.2415	.1731	.1039	− 0.0987	− 0.0423	0.0602	$\mathscr{L}\mathscr{F}_2$
IFWG^70^	–	–	–	–	–	–	–	Failed
PFWG^71^	− .2638	.1891	0.0905	− 0.0806	− .5160	− .3853	− .1894	$\mathscr{L}\mathscr{F}_2$
FFWG^73^	− .2236	.1240	0.0522	− 0.0803	− .4155	− .3098	− .1628	$\mathscr{L}\mathscr{F}_2$
4-ROFWG^74^	− .1748	0.0762	0.0272	− 0.0677	− .3340	− .2397	− .1268	$\mathscr{L}\mathscr{F}_2$
BPFWG$\phantom{0}_{w}$^42^	− .1229	.3237	.2017	0.0533	− .3413	− .2260	− 0.0547	$\mathscr{L}\mathscr{F}_2$
B3-ROFWG^15^	− 0.0976	.2464	.1509	0.0335	− .2573	− .1701	− 0.0455	$\mathscr{L}\mathscr{F}_2$
B4-ROFWG^15^	− 0.0744	.1865	.1092	0.0195	− .1948	− .1257	− 0.0361	$\mathscr{L}\mathscr{F}_2$
C2-ROFWG^9^	− .1885	.1958	.1058	− 0.0289	− .4231	− .3118	− .1120	$\mathscr{L}\mathscr{F}_2$
C3-ROFWG^9^	− .1629	.1333	0.0644	− 0.0412	− .3360	− .2511	− .1030	$\mathscr{L}\mathscr{F}_2$
C4-ROFWG^9^	− .1285	0.0851	0.0360	− 0.0405	− .2618	− .1919	− 0.0838	$\mathscr{L}\mathscr{F}_2$
BCFWGA^75^	.4327	.6501	.5961	.5240	.2535	.3382	.4740	$\mathscr{L}\mathscr{F}_2$
BCIFWG^20^	–	–	–	–	–	–	–	Failed
Proposed BC2-ROFWG	− 0.0985	.3397	.2325	0.0965	− .3434	− .2287	− 0.0073	$\mathscr{L}\mathscr{F}_2$
Proposed BC3-ROFWG	− 0.0785	.2663	.1813	0.0697	− .2614	− .1741	− 0.0083	$\mathscr{L}\mathscr{F}_2$
Proposed BC4-ROFWG	− 0.0590	.2040	.1355	0.0473	− .1938	− .1267	− 0.0086	$\mathscr{L}\mathscr{F}_2$

## **Sensitivity and limitations of aggregation operators**

This section presents a detailed sensitivity assessment of the proposed aggregation operators, evaluating their robustness under different parameter settings. In addition, we discuss the inherent limitations of these methods, highlighting areas that may require careful consideration or further enhancement in practical applications.

### **Sensitivity evaluation of aggregation parameters**

To examine the responsiveness of the BCq-ROFWA and BCq-ROFWG operators, we systematically varied the parameter *q* across a wide range of values. The parameter *q* directly influences the compensatory behavior of the aggregation process: smaller *q* values lead to more restrictive, non-compensatory aggregation, whereas larger *q* values introduce greater compensation between membership and nonmembership degrees.

Table 9 summarizes the resulting scores for a representative set of alternatives under different *q* values. The analysis reveals that, as *q* increases, the score values for all alternatives gradually decrease, indicating a convergence trend. Despite the reduction in absolute score reals, the ranking of alternatives remains unchanged, with $\mathscr{L}\mathscr{F}_2$ consistently identified as the top choice across all tested *q* values. This outcome underscores the isotonic stability of the proposed operators, suggesting that decision-makers can adjust *q* to modulate aggregation intensity without impacting the final ranking outcome.

**Fig. 8:**
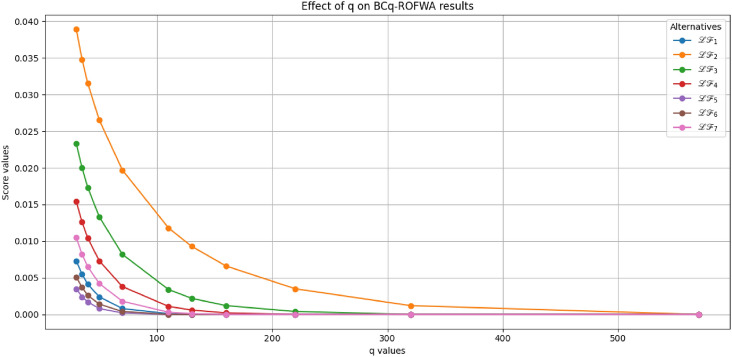
Score trends based on Table 9.

Moreover, the decreasing trend in score reals highlights the operators’ sensitivity to extreme *q* values. Very large *q* values yield scores that are nearly negligible for all alternatives, which may reduce the discriminative power between options. Conversely, moderate *q* values maintain sufficient variation among alternatives, ensuring reliable differentiation and interpretable decision outcomes. A graphical representation of this behavior in Fig. 8 clearly illustrates how the real of *q* influences the aggregated results.

### **Limitations of the proposed aggregation operators**

While the BCq-ROFWA and BCq-ROFWG operators provide a flexible and robust framework for MADM, several limitations are evident from the sensitivity analysis:

1. Score convergence at extreme *q* values: Very large *q* values lead to almost uniform, near-zero scores, which diminishes the ability to distinguish between alternatives effectively. Decision-makers must avoid extreme settings to preserve meaningful differentiation.
2. Optimal performance at moderate *q*: The operators deliver their best discriminative capability when *q* is selected within a reasonable range. Small to moderate values allow the model to reflect nuanced differences between alternatives, ensuring accurate rankings.
3. Computational and interpretational considerations: As *q* and the number of alternatives increase, the computational demand grows due to more extensive aggregation operations. Additionally, interpreting results for a large set of alternatives may require supplemental techniques, such as weighting schemes or dimensionality reduction, to maintain clarity.

In summary, the sensitivity assessment demonstrates that the proposed operators are robust and stable for a wide range of practical *q* values, with rankings remaining consistent even under parameter variation. Future work may focus on enhancing their discriminative capacity under extreme parameter settings and extending the framework to efficiently handle large-scale, high-dimensional MADM problems.

**Table 9:** Effect of *q* on aggregation results.

*q*	Score values of							Operator
	$\mathscr{L}\mathscr{F}_1$	$\mathscr{L}\mathscr{F}_2$	$\mathscr{L}\mathscr{F}_3$	$\mathscr{L}\mathscr{F}_4$	$\mathscr{L}\mathscr{F}_5$	$\mathscr{L}\mathscr{F}_6$	$\mathscr{L}\mathscr{F}_7$	
30	.0073	.0389	.0233	.0154	.0035	.0051	.0105	BCq-ROFWA
	− .0001	.0040	.0011	.0000	− .0027	− .0003	− .0000	BCq-ROFWG
35	.0055	.0348	.0200	.0126	.0024	.0037	.0082	BCq-ROFWA
	− .0000	.0023	.0006	.0000	− .0016	− .0001	− .0000	BCq-ROFWG
40	.0041	.0315	.0173	.0104	.0017	.0026	.0065	BCq-ROFWA
	− .0000	.0014	.0003	.0000	− .0010	− .0000	− .0000	BCq-ROFWG
50	.0024	.0265	.0133	.0073	.0008	.0014	.0042	BCq-ROFWA
	− .0000	.0004	.0000	.0000	− .0003	− .0000	− .0000	BCq-ROFWG
70	.0008	.0197	.0082	.0038	.0002	.0004	.0018	BCq-ROFWA
	− .0000	.0000	.0000	− .0000	− .0000	− .0000	− .0000	BCq-ROFWG
110	.0001	.0118	.0034	.0011	.0000	.0000	.0003	BCq-ROFWA
	− .0000	.0000	.0000	− .0000	− .0000	− .0000	− .0000	BCq-ROFWG
130	.0000	.0093	.0022	.0006	.0000	.0000	.0001	BCq-ROFWA
	− .0000	.0000	.0000	.0000	− .0000	− .0000	− .0000	BCq-ROFWG
160	.0000	.0066	.0012	.0002	.0000	.0000	.0000	BCq-ROFWA
	− .0000	.0000	.0000	.0000	− .0000	− .0000	.0000	BCq-ROFWG
220	.0000	.0035	.0004	.0000	.0000	.0000	.0000	BCq-ROFWA
	− .0000	.0000	.0000	.0000	− .0000	− .0000	.0000	BCq− ROFWG
320	.0000	.0012	.0000	.0000	.0000	.0000	.0000	BCq-ROFWA
	− .0000	.0000	.0000	.0000	− .0000	− .0000	.0000	BCq-ROFWG
570	.0000	.0000	.0000	− .0000	− .0000	.0000	.0000	BCq-ROFWA
	− .0000	.0000	.0000	.0000	− .0000	− .0000	.0000	BCq-ROFWG

## Conclusions and future work

This study introduced a comprehensive MADM framework based on BCq-ROFS, specifically designed to handle complex, uncertain, and dual (positive-negative) evaluations. The work began with the formulation of rigorous mathematical foundations for BCq-ROFS, establishing its theoretical consistency and supporting the development of two novel aggregation operators: the BCq-ROF weighted averaging (BCq-ROFWA) and weighted geometric (BCq-ROFWG) operators. These operators are structured to capture both bipolar evaluations and complex-valued uncertainties, while allowing flexible compensation behavior via the *q* parameter. The proposed framework was applied to a multi-attribute decision-making problem in sustainable livestock farming, evaluating several alternative practices across environmental, economic, and social dimensions. Results consistently identified the most balanced and sustainable alternative, demonstrating the operators’ ability to integrate positive and negative perspectives across multiple attributes. Comparative analyses showed that BCq-ROFWA and BCq-ROFWG provide improved ranking stability, discrimination power, and interpretability relative to traditional fuzzy, q-rung orthopair, and bipolar complex fuzzy aggregation approaches, confirming the framework’s superior capability in modeling nuanced, multidimensional uncertainties.

Key contributions of this framework include its unified and generalized modeling capability, allowing the proposed operators to reduce to existing fuzzy set-based operators—such as IFS, PFS, FFS, BPFS, Bq-ROFS, and BCFS—under specific parameter constraints. By incorporating bipolarity, complex-valued membership, and the flexible q-rung structure, the framework enhances uncertainty representation, providing a more comprehensive and realistic modeling of conflicting, oscillatory, and dual-imaginary information than existing approaches. Sensitivity analyses further confirmed that rankings remain robust under varying *q* values, while extreme *q* settings highlight practical considerations for operator discriminative capacity and computational efficiency. Despite these strengths, the study has limitations, including the focus on a single application domain, reliance on expert-provided weights and attribute evaluations, and sensitivity analyses that do not fully account for dynamic or time-varying conditions. Computational demands may increase with larger numbers of alternatives and attributes, requiring efficient implementation for large-scale problems.

Future research can extend this framework in several directions:

- Application of BCq-ROFS to diverse domains such as renewable energy planning, smart water management, healthcare, and climate-resilient infrastructure.
- Integration with intelligent decision-support systems, machine learning, or optimization algorithms to enable dynamic, real-time decision-making under uncertainty.
- Exploration of alternative fuzzy paradigms, such as spherical or higher-order q-rung orthopair fuzzy sets, to further enhance modeling flexibility and aggregation precision.
- Development of computational tools for efficient, scalable implementation in large-scale, high-dimensional MADM problems.
- Incorporation of IoT-enabled monitoring and AI-driven predictive analytics to facilitate adaptive, data-driven decision support in dynamic environments.

## Data Availability

All data generated or analysed during this study are included in this published article.
